# Crossing the Threshold: Using Photostationary States to Control Supramolecular Polymerisation

**DOI:** 10.1002/anie.5794634

**Published:** 2026-09-30

**Authors:** Deepu George, Jiarong Wu, Leo Greb, Vladimir Stepanenko, Aidan Izuagbe, Lutz Nuhn, Frank Würthner, Jake L. Greenfield

**Affiliations:** ^1^ EaStCHEM School of Chemistry University of St Andrews Fife UK; ^2^ Institut für Organische Chemie and Center for Nanosystems Chemistry (CNC) Universität Würzburg Würzburg Germany; ^3^ Institute of Functional Materials and Biofabrication Center of Polymers for Life Universität Würzburg Würzburg Germany

**Keywords:** arylazopyrazole, photostationary state, photoswitch, supramolecular polymerisation, threshold behaviour

## Abstract

The completeness of photoswitching is a key figure of merit for molecular photoswitches. In azo‐based switches, quantitative conversion between the *E* and *Z* isomers is typically sought to maximise change in properties, while intermediate photostationary states (PSSs) receive less attention. Here, we show that intermediate PSSs can be functionally useful when coupled to threshold‐regulated processes. We demonstrate this by using a photoswitchable monomer that undergoes cooperative supramolecular polymerisation. The irradiation wavelength controls the concentration of polymerisation‐active monomer; once this concentration crosses the nucleation threshold, small wavelength‐dependent changes in PSS composition produce large changes in aggregation state. The resulting supramolecular polymer assembles in aqueous media and undergoes light‐induced disassembly in the condensed phase. In the broader context, coupling photoswitching to threshold‐dependent assembly provides a novel route to convert subtle changes in PSS composition into an amplified, non‐linear supramolecular response.

## Graphical abstract

Intermediate photostationary states regulate the concentration of a polymerisation‐active monomer. Once the nucleation threshold is crossed, small wavelength‐dependent changes in isomer composition are amplified into a non‐linear supramolecular assembly response. This shows that intermediate PSSs are not merely incomplete switching states, but can be useful for controlling threshold‐dependent chemical systems.

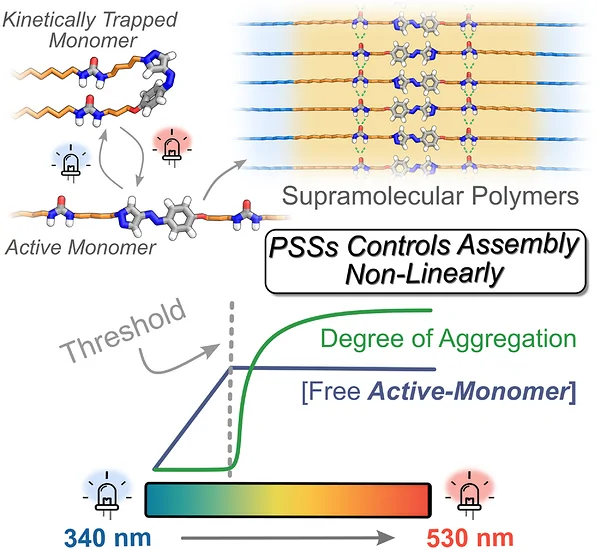

Light can be used to control the structure [1, 2, 3, 4], function [5, 6, 7, 8, 9, 10, 11], and composition [12, 13, 14] of closed chemical systems with high spatiotemporal precision [15, 16]. Among the various photochromic motifs, molecular photoswitches are attractive because they reversibly interconvert between two or more distinct states [1, 3]. For azo‐based switches (N═N), a key figure of merit is the completeness of *E*/*Z* photoswitching [1]. Consequently, many studies that apply these switches focus on using the *E*‐rich and *Z*‐rich states, where the largest changes in system properties are often expected [17, 18, 19, 20, 21]. By contrast, the properties and behaviour that arise from intermediate photostationary states (PSSs), which contain defined and reproducible mixtures of *E*‐ and *Z*‐isomers, are less commonly explored [22].

For P‐type photoswitches and T‐type photoswitches with long‐lived metastable states, intermediate PSSs provide a reliable way to generate defined isomer concentrations at a fixed temperature. These states can also give rise to behaviour that is not a simple average of the two limiting isomeric forms. For example, Giese and co‐workers recently reported a photoswitchable liquid‐crystalline system in which an intermediate PSS produced a distinct phase that was not accessed from either the *E*‐rich or *Z*‐rich states alone [22]. In their system, the isomer composition is coupled to a secondary process, namely liquid‐crystalline ordering [22], rather than simply affording the average properties of the *E‐* and *Z*‐isomers.

Inspired by this, we asked whether intermediate PSSs could be used to generate non‐linear responses in supramolecular systems. In particular, we reasoned that wavelength‐dependent changes in isomer composition could be used to drive the concentration of an assembly‐active monomer across a threshold, thereby converting small changes in PSS composition into a non‐linear assembly response (Figure 1d) [23]. Such threshold responses are common in biological systems [24]; for example, actin assembly is regulated by the concentration of active ATP‐actin monomers, with filament growth only occurring when the free monomer concentration exceeds a critical concentration [25].

**FIGURE 1:**
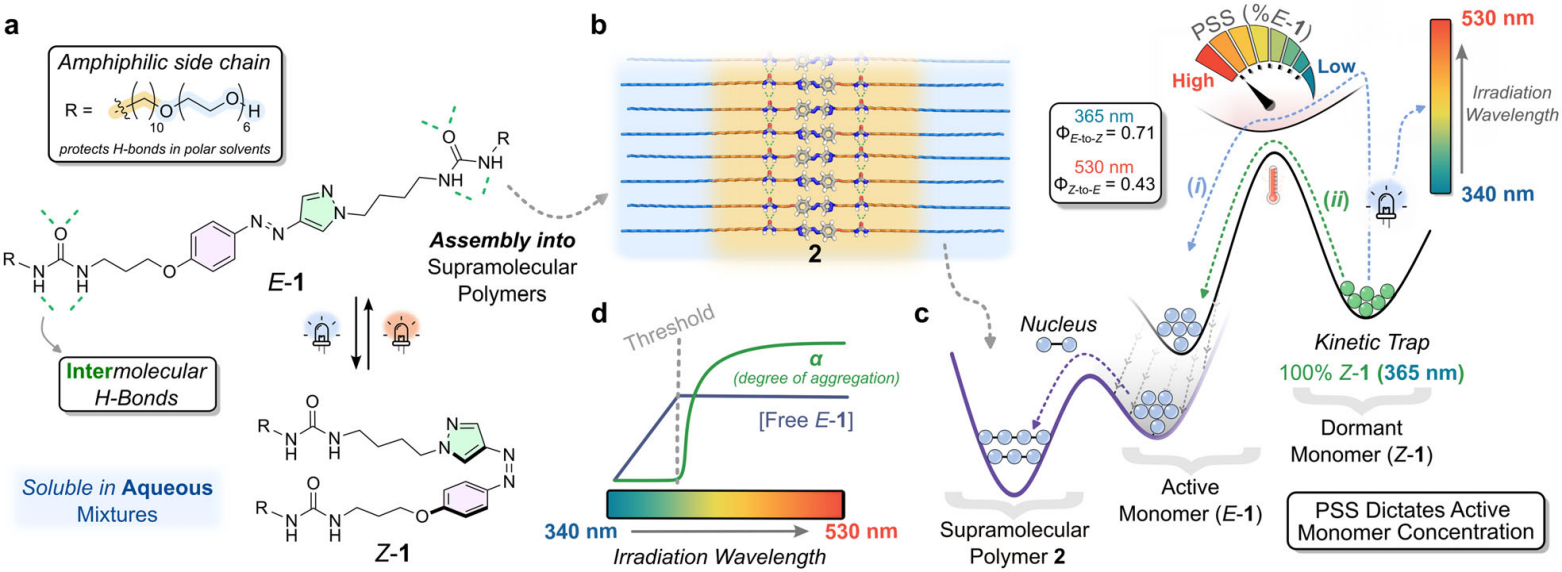
(a) Structures of monomer **1** in the *E*‐ and *Z*‐isomeric forms. The appended R‐groups correspond to the amphiphilic side chains, which shield the urea hydrogen bonds (*green dashed lines*). (b) *E*‐**1** forms intermolecular hydrogen bonds to assemble into polymer **2**. The shaded orange and blue regions represent the hydrophobic and hydrophilic parts of the amphiphilic side chain, respectively. (c) Schematic illustration of the supramolecular polymerisation of *E*‐**1** into **2** (route *i*). *Z*‐**1** is a kinetically trapped state with an activation barrier for the thermal *Z*‐to‐*E* conversion (*∆G* ^‡^ *_Z_*_‐to‐_ *_E_*, route (*ii*)) of 111.3 kJ mol^−1^. Irradiation at 365 nm quantitatively converts *E*‐**1** into *Z*‐**1**, suppressing supramolecular polymerisation, while irradiation at 530 nm regenerates *E*‐**1**. Varying the irradiation wavelength from 340 to 530 nm generates different PSS compositions of *E*‐**1** and *Z*‐**1**. When the wavelength used produces an *E*‐**1** concentration above the nucleation threshold (*free‐energy surface represented by the black line*), supramolecular polymers of **2** form (*free‐energy surface represented by the purple line*). (d) Schematic illustration showing how irradiation wavelength controls the concentration of free, non‐assembled *E*‐**1** and the degree of aggregation (⍺). Coupling PSS composition to nucleation–elongation gives a non‐linear response once the threshold concentration is crossed.

We therefore hypothesised that a light‐responsive system could be realised by coupling photoswitching to cooperative supramolecular polymerisation [26, 27]. In such a system, intermediate PSSs determine the concentration of the polymerisation‐active monomer, while the threshold behaviour of the assembly process converts modest wavelength‐dependent changes in isomer composition into pronounced changes in aggregation state [28]. Photoresponsive supramolecular polymers have been reported previously [26, 27, 29, 30, 31, 32, 33, 34, 35], including systems with excellent light‐controlled assembly and disassembly. However, the role of intermediate PSSs and the non‐linear response that can emerge around the threshold concentration has received comparatively little attention.

Here, we report a minimal light‐responsive self‐assembling system based on an amphiphilic arylazopyrazole bis‐urea monomer, **1**, that is soluble in aqueous solvent mixtures (Figure 1a,b). Using accessible LED wavelengths, we show that different PSSs can control the assembly of supramolecular polymer **2** through a cooperative nucleation–elongation mechanism (Figure 1c). Crossing the nucleation threshold gives a step‐change in the degree of aggregation (⍺), enabling wavelength‐dependent control over assembly at room temperature (Figure 1d). We also show that polymer **2** remains photoresponsive in the condensed phase. This approach provides a general strategy for designing reversible non‐linear, wavelength‐dependent responses in supramolecular and systems chemistry.

Monomer **1** contains a single photoswitchable unit, which simplifies the relationship between the overall *E*/*Z* composition at each PSS and the concentrations of *E*‐**1** and *Z*‐**1** [5]. The switchable unit is an arylazopyrazole motif, selected for its efficient bidirectional photoswitching and its long‐lived metastable *Z*‐state [17, 32, 36, 37]. This class of photoswitches also provides broad coverage of PSS compositions, from 0% to 100% *Z*‐**1**, using common LED wavelengths. To achieve assembly in aqueous media, the monomer contains an amphiphilic side chain [29, 38]. In this approach, hydrophobic aliphatic segments shield the hydrogen‐bonding motif from the polar medium, while oligo(ethylene glycol)‐type segments provide solubility. *E*‐**1** was synthesized through a modular route and purified by recycling GPC (Section S1) [39].

We first examined the intrinsic photoswitching properties of **1** in the absence of assembly. These studies were performed in DMSO, which solvates the amphiphilic side chains and, as a strong hydrogen‐bond acceptor, prevents supramolecular polymerisation. Under these conditions, **1** displayed quantitative bidirectional photoswitching between the *E*‐ and *Z*‐isomers [37, 40]. Irradiation of a solution of **1** at 365 nm afforded a PSS containing 100% *Z*‐**1**, as determined by UV/vis (Figure 2) and ^1^H NMR spectroscopy (Figure S8). Quantitative *Z*‐to‐*E* photoisomerisation was achieved under visible‐light irradiation at 530 nm (Figure 2). Irradiation at other wavelengths gave a series of intermediate PSSs (Figure 2), which were subsequently used to control assembly (vide infra). The photoisomerisation was efficient, with quantum yields of 0.71 for *E*‐to‐*Z* isomerisation at 365 nm and 0.43 for *Z*‐to‐*E* isomerisation at 530 nm, determined using the procedure reported by Crespi and co‐workers (Section S3.5) [41]. Finally, *Z*‐**1** showed high thermal stability towards relaxation back to *E*‐**1**, with a thermal half‐life (t_1/2_) of 90.5 days at 20°C (293 K) (Figure S7). This long t_1/2_ ensures that the PSS distributions remain stable over the timescale of the assembly experiments.

**FIGURE 2:**
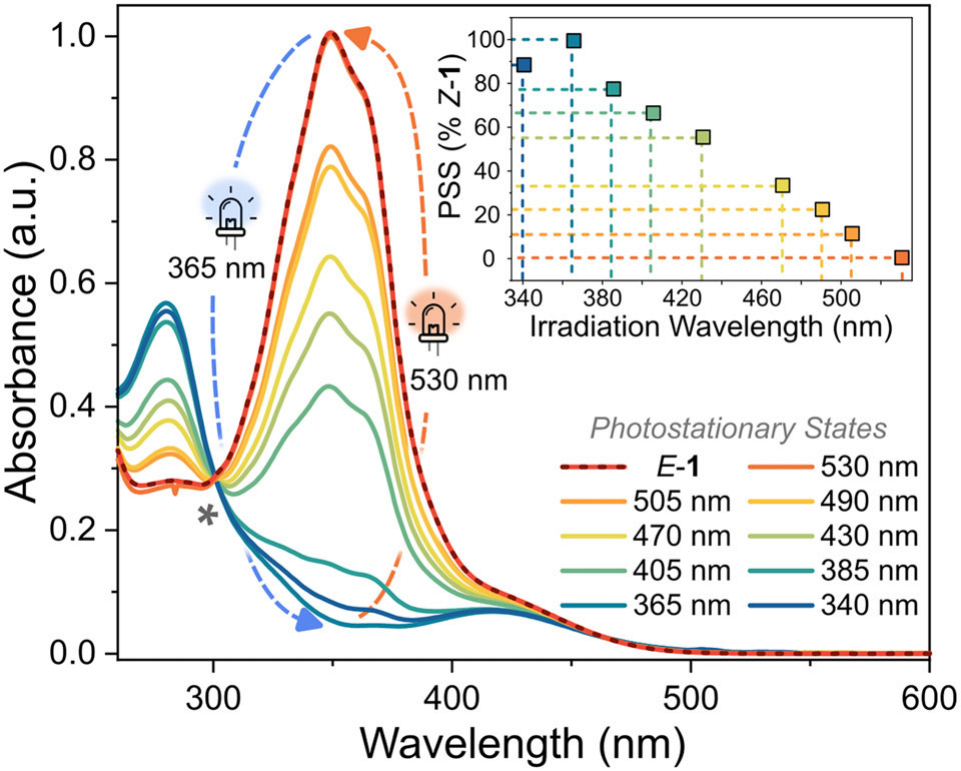
UV/vis absorption spectra of a 42 µM solution of *E*‐**1** in DMSO at 20°C (293 K) after irradiation at different wavelengths. (***Inset***) Irradiation wavelengths from 340 to 530 nm provide broad coverage of isomer compositions, from 0% *Z*‐**1** to 100% *Z*‐**1**. Irradiation at 365 and 530 nm affords quantitative *E*‐to‐*Z* and *Z*‐to‐*E* switching, respectively. The asterisk denotes an isosbestic point.

Next, a solvent screen was performed to identify conditions in which *E*‐**1** undergoes supramolecular polymerisation to form **2** (Section S4.1). When a stock solution of *E*‐**1** (53 µL, 2 mM, H_2_O/MeCN, 1:1 *v/v*) was dispersed into H_2_O/MeCN (3 mL, 4:1 *v/v*), the UV/vis absorption spectrum changed markedly (Figures S14 and S15). The π–π* band (ca. 350 nm) broadened and shifted hypsochromically, consistent with the formation of H‐type aggregates. The photochromic units are proposed to adopt a head‐to‐tail arrangement within these aggregates to minimize dipole moments, analogous to the arrangement observed in the liquid‐crystalline system reported by Giese and co‐workers [22].

The morphology of the aggregates was examined by AFM, which revealed rope‐like fibres with a visible helical twist along their length (Figure 3a). Both *P* and *M* helical fibres were observed, consistent with the achiral structure of monomer **1** (Figure S24). The measured heights of the fibres are consistent with the expected diameter of **2**, estimated from molecular mechanics modelling (Figure S18). Statistical analysis of the AFM data showed that fibre lengths reach up to a few microns (Section S5.2). To confirm that the fibres were not only simply formed during the solvent evaporation step while preparing the thin films, dynamic light scattering (DLS) measurements were also performed. Large aggregates were observed for an 80 µM sample of *E*‐**1**, consistent with the presence of larger polymeric species in solution (600–1500 nm, Figure S17). Together, these data support the formation of supramolecular polymers from this minimal monomer design in aqueous solvent mixtures.

**FIGURE 3:**
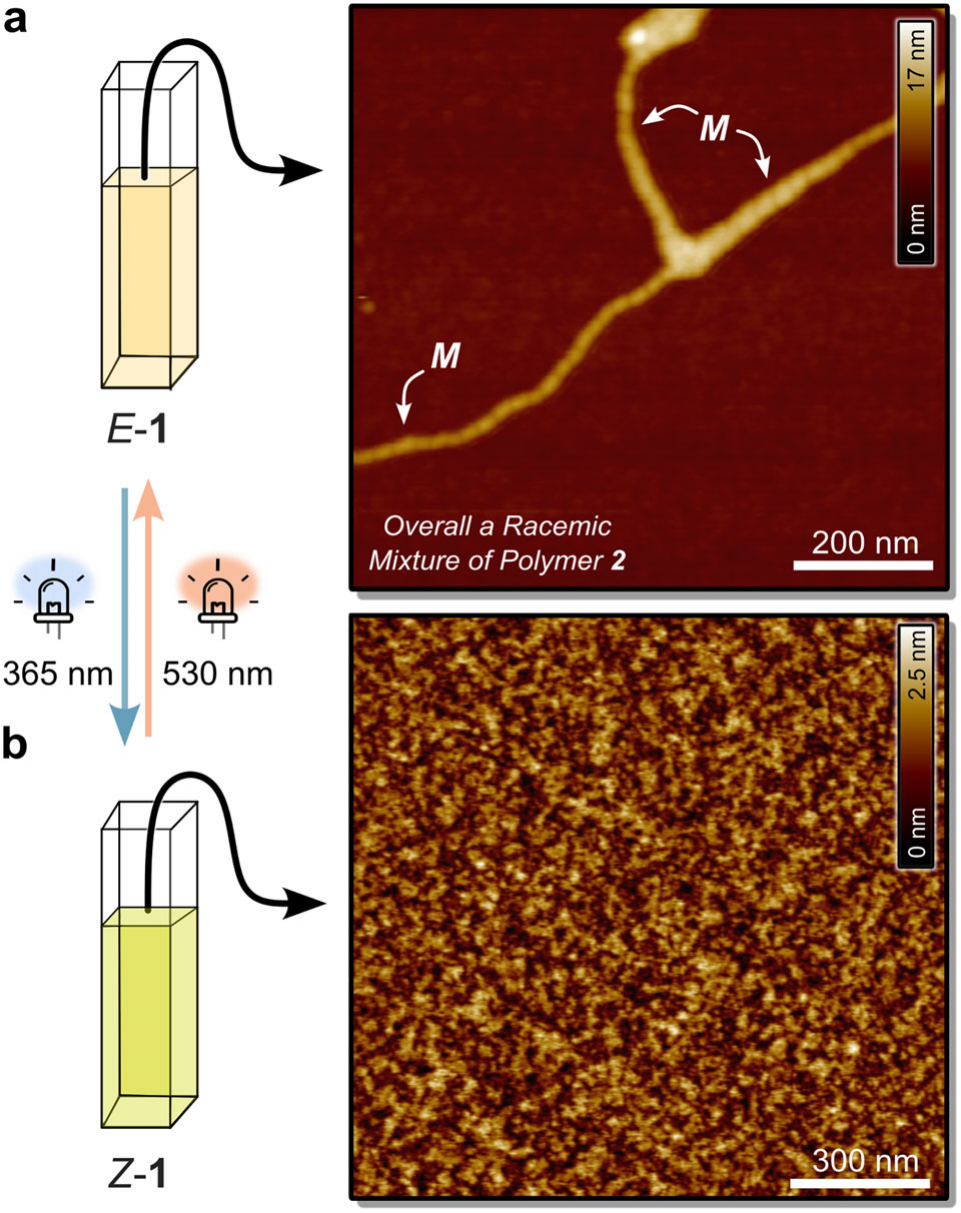
AFM images of drop‐cast films prepared from (a) *E*‐**1** (35 µM solution of **1** in H_2_O/MeCN (4:1, *v/v*) at 20°C (293 K), irradiated to 530 nm PSS before drop‐casting and drying), and (b) *Z*‐**1** (35 µM solution of **1** in H_2_O/MeCN (4:1, *v/v*) at 20°C (293 K), converted to the 365 nm PSS before drop‐casting and drying). In (a), helical fibres of **2** are observed as a racemic mixture of *M‐* and *P‐* helices (see Section S5.2). In (b), no fibre‐like structures are observed.

Having established that *E*‐**1** forms helical polymers of **2** in H_2_O/MeCN, we investigated the supramolecular polymerisation mechanism using temperature‐dependent UV/vis spectroscopy [42]. At 10°C (283 K), *E*‐**1** is fully assembled into chains of **2** (degree of aggregation, *α* = 1; Figure 4a). Upon heating at 0.4°C min^−1^, **2** progressively disassembles, with an isosbestic point at 330 nm implying a clean transition between assembled and disassembled states. At 60°C (333 K), the UV/vis spectrum resembles that of non‐aggregated *E*‐**1**, indicating complete disassembly (α = 0). The cooling curve (Figure 4a) shows an elongation temperature (*T*_E_) of ca. 48°C (321 K), below which assembly of *E*‐**1** into **2** begins. This behaviour is consistent with cooperative polymerisation. A comparison between cooperative and isodesmic fitting is provided in Section S4.3. Fitting the data to a nucleation–elongation model gave an elongation enthalpy of Δ*H*_E_ = −68.49 ± 1.13 kJ mol^−1^ and *T*_E_ = 48°C (321 K). The same study at two different concentrations, 25 and 45 µM, showed the expected concentration dependence of *T*_E_ and supported a cooperative supramolecular polymerisation mechanism in aqueous media (Section S4.3).

**FIGURE 4:**
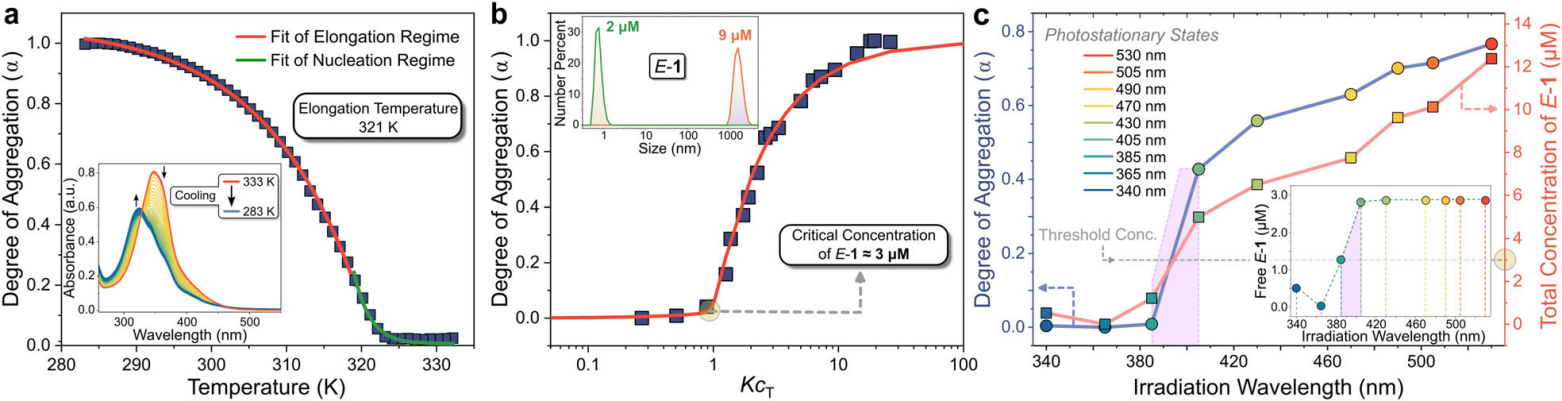
(a) Degree of aggregation (⍺) as a function of temperature during cooling of *E*‐**1** (35 µM in H_2_O/MeCN 4:1, *v*/*v*) from 60°C (333 K) to 10°C (283 K) at 0.4°C min^−1^. At 60°C (333 K), *E*‐**1** is monomeric, whereas at 10°C (283 K) it is fully assembled as **2**. The data were fitted to a nucleation–elongation model (*green* and *red* traces). (***Inset***) Corresponding temperature‐dependent UV/vis absorption spectra. (b) Degree of aggregation (⍺) as a function of concentration at 20°C (293 K), fitted to a nucleation–elongation model, where *K* is the equilibrium association constant for elongation, and c_T_ is the total concentration of *E*‐**1**. (***Inset***) DLS size distributions, shown as number percent, below and above the threshold concentration. (c) Degree of aggregation (⍺, *left axis*) and total *E*‐**1** concentration (*right axis*) for a sample of **1** (12.5 µM, 293 K) at different PSSs. The onset of polymerisation is highlighted in purple. This sharp onset between 385 and 405 nm shows the non‐linear response of the system. (***Inset***) Concentration of free, non‐assembled *E*‐**1** at different PSSs, showing buffering of the free monomer concentration by the nucleation–elongation mechanism. The threshold concentration of *E*‐**1** is indicated on the right axis by a yellow circle.

The threshold concentration for nucleation–elongation was then determined by measuring the degree of aggregation as a function of [*E*‐**1**] (Figure 4b). The UV/vis spectra of these samples were monitored over time to ensure equilibrium had been reached (Figure S22). Fitting these data to a nucleation–elongation model gave a threshold concentration of 3 µM for *E*‐**1** at 20°C (293 K) [43]. DLS measurements performed above and below this concentration revealed the presence and absence of large particles, respectively (Figure 4b, inset).

We then verified that only *E*‐**1** assembles into supramolecular polymer **2** under these conditions. Taking a sample of **2** in H_2_O/MeCN (35 µM; 4:1, *v/v*) and irradiating it with 365 nm light afforded a UV/vis absorption spectrum consistent with *Z*‐**1** (Figure S16). No changes in the *Z*‐**1** UV/vis spectrum were observed after the sample was left at 20°C (293 K) for 90 mins, confirming that the metastable isomer is sufficiently long‐lived under these conditions. Subsequent irradiation with 530 nm light converted *Z*‐**1** back to *E*‐**1** and regenerated the initial UV/vis absorption spectrum of **2**. AFM supported this reversible light‐triggered change in morphology: samples that contained fibres before irradiation no longer showed fibre‐like objects after irradiating a solution with 365 nm light and drop‐casting onto a silicon wafer (Figure 3b). This light‐induced disassembly process was further supported by DLS studies of the *Z*‐rich samples, revealing small species ranging from 8–15 nm in size (Figure S17).

We next examined whether intermediate PSSs could be used to control supramolecular polymerisation at a fixed temperature (Figure 4c). At a total monomer concentration of 12.5 µM, the accessible PSS compositions place the concentration of *E*‐**1** either below or above the threshold concentration for nucleation. Using the intrinsic PSS distributions measured in DMSO as a guide (Figure 2), we investigated the wavelength dependence of assembly. Control measurements were also performed to assess the PSS distribution of monomer **1** in H_2_O/MeCN (4:1, *v/v*) at 2.5 µM, a concentration below the critical concentration. These PSS compositions were in agreement with those measured in DMSO (Figure S10). Wavelengths that keep [*E*‐**1**] below the threshold, from 340 to 385 nm, do not result in supramolecular polymerisation, evidenced by α of ca. 0 (Figure 4c). In contrast, wavelengths that generate [*E*‐**1**] above the nucleation threshold, from 405 to 530 nm, induce supramolecular polymerisation, with the degree of aggregation increasing upon bathochromically shifting the irradiation wavelength (Figure 4c). This light‐induced assembly and disassembly can be repeated multiple times without observable fatigue of the photoswitch (Figure S31).

Below the threshold concentration, increasing the *E*‐isomer fraction increases the concentration of free *E*‐**1** in solution linearly (Figure 4c, inset). Once the total concentration of *E*‐**1** exceeds 3 µM, long supramolecular polymers begin to form, as supported by DLS measurements at different PSSs (Figure S30 and Table S8). Above this threshold, the nucleation–elongation mechanism buffers the concentration of free *E*‐**1** at ca. 3 µM (Figure 4c, inset). Additional *E*‐**1** generated at more *E*‐rich PSSs (405–530 nm) is instead incorporated into the growing polymer chains, leading to an increase in the degree of aggregation. The system therefore converts wavelength‐dependent changes in *E*/*Z* composition into a non‐linear change in assembly state: below the threshold, *E*‐**1** accumulates as free monomer; above it, additional *E*‐**1** contributes to polymer growth.

This behaviour highlights why PSS control is useful in this system. In principle, the *E*/*Z* ratio could also be tuned by varying the irradiation time at a wavelength, prior to reaching a PSS. However, this approach depends strongly on photon flux at the sample, making it challenging to reliably reproduce when the experimental setup is altered. By contrast, generating a PSS provides a reproducible isomer distribution. The approach therefore resembles a concentration‐dependent supramolecular polymerisation study, but with the active monomer concentration set by the irradiation wavelength rather than by preparing separate samples.

Having established the solution‐state behaviour, we examined whether photoisomerisation could also perturb the assembled state in the condensed phase. We postulated that this might be possible because the appended amphiphilic chains may provide sufficient local flexibility for isomerisation within the fibres. Recent high‐speed AFM studies by Feringa, Roos, and coworkers showed that, in solution, disassembly of related supramolecular polymers can be observed with AFM [31]. Thin films of **2** were prepared by drop‐casting onto quartz substrates. Upon irradiation with 365 nm light, the UV/vis spectrum changed in a manner consistent with *E*‐to‐*Z* photoisomerisation (Figure S23). Irradiation of the switched film with 530 nm returned the pre‐irradiated spectrum.

To investigate this behaviour, **2** was deposited onto a silicon wafer and analysed by AFM with in situ light irradiation (365 nm). Before irradiation, intact fibres were clearly observed. After 3 min of irradiation of the substrate in the AFM, the fibres had fragmented into shorter chains. Longer irradiation times led to progressively greater fragmentation, until fibre‐like structures were no longer observable (Figure S28). Control experiments showed no evidence of tip‐induced damage from repeated AFM imaging (Section S5.2).

In summary, we have developed a minimal light‐responsive system in which PSS composition is coupled to a concentration‐threshold‐dependent assembly process. This represents a mechanistically distinct, yet complementary strategy to recent light‐intensity‐based approaches to controlling supramolecular nucleation and structure [44, 45]. Specifically, this work demonstrates how a cooperative nucleation threshold can amplify even small changes in PSS composition into a non‐linear supramolecular response.

## Author Contributions

**Deepu George**: investigation, writing–original draft, methodology, writing–review and editing. **Jiarong Wu**: methodology, writing–original draft, writing–review and editing, supervision. **Leo Greb**: methodology, investigation, writing–original draft. **Vladimir Stepanenko**: investigation. **Aidan Izuagbe**: investigation. **Lutz Nuhn**: resources, writing–review and editing. **Frank Würthner**: resources, supervision, writing–review and editing. **Jake L. Greenfield**: funding acquisition, writing–original draft, writing–review and editing, conceptualisation, resources, project administration, supervision.

## Conflicts of Interest

The authors declare no conflicts of interest.

## Supporting information



Synthetic details and characterisation data (PDF). The data that support the findings of this study are openly available on Pure at https://doi.org/10.17630/351bc776‐e8d0‐4bb1‐8a88‐5889f47a6698. **Supporting File 1**: anie74970‐sup‐0001‐SuppMat.pdf.

## Data Availability

The data that support the findings of this study are openly available on Pure at https://doi.org/10.17630/351bc776‐e8d0‐4bb1‐8a88‐5889f47a6698.
