# Supplementary material for: Crossing the Threshold: Using Photostationary States to Control Supramolecular Polymerisation
Source: Angew Chem Int Ed Engl. 2026 Sep 30;65(46):e5794634. doi: 10.1002/anie.5794634 (PMC13749354; doi:10.1002/anie.5794634)
Supplement: Supplementary file 1 [file ANIE-65-e5794634-s001.pdf]

Supporting Information

Crossing the Threshold: Using Photostationary States to Control Supramolecular Polymerisation

Deepu George,[a],[b] Jiarong Wu,[a],[b] Leo Greb,[b] Vladimir Stepanenko,[b] Aidan Izuagbe,[c] Lutz Nuhn,[c] Frank Würthner,[b] and Jake L. Greenfield[a],[b]*

[a] Deepu George, Dr. Jiarong Wu and Dr. Jake L. Greenfield
EaStCHEM, School of Chemistry,
University of St Andrews, St Andrews,
Fife, KY16 9ST UK
jlg28@st-andrews.ac.uk

[b] Deepu George, Dr. Jiarong Wu, Leo Greb, Prof. Dr. Frank Würthner and Dr. Jake L. Greenfield
Institut für Organische Chemie and Center for Nanosystems Chemistry (CNC),
Universität Würzburg,
97074 Würzburg, Germany

[c] Dr. Aidan Izuagbe and Prof. Dr. Lutz Nuhn
Institute of Functional Materials and Biofabrication,
Center of Polymers for Life,
Universität Würzburg,
97074 Würzburg, Germany

Table of Contents

1. Materials and Methods 3
2. Synthesis and Characterization 7
  2.2. Synthesis of the Sidechain 12
3. UV/vis and Photoswitching Studies 22
  3.1. Extinction Coefficient 22
  3.2. Thermodynamic Properties 23
  3.3. Photostationary State (PSS) 25
  3.4. Fatigue Resistance 28
  3.5. Quantum Yields 29
4. Investigation of the Polymerization Process 32
  4.1. Solvent Screening 32
  4.2. DLS Studies 37
  4.3. Investigation of the Polymerization Mechanism 39
    4.3.1. Temperature-dependent UV/vis Measurements of *E*-**1** 40
    4.3.2. Concentration-dependent UV/vis Measurements of *E*-**1** 42
5. Condensed Phase Studies 44
  5.1. Condensed Phase Photoswitching 44
  5.2. AFM Measurements 45
    5.2.1. Morphology of *E*-**1** Assemblies 45
    5.2.2. Morphology of *Z*-**1** 47
    5.2.3. AFM with in-situ irradiation 48
    5.2.4. AFM Control Experiment 49
6. PSS-modulated Aggregation 50
  6.1 Crossing the Nucleation Threshold with Light 50
  6.2 Resistance to Fatigue During Assembly and Disassembly 52
7. References 53

1. Materials and Methods

All reagents and solvents were purchased from commercial suppliers and used without further purification, unless specified. Anhydrous solvents were obtained from the commercial solvent purification system PS-M6-6/7-En of Inert Technologies. All synthesized compounds were stored under N_2, kept in dark, and dried under high vacuum for at least 24 h prior to use.

Synthesis. Air or moisture sensitive reactions were carried out under a nitrogen atmosphere under standard Schlenk conditions.

Purification. Column chromatography was performed using standard glass columns of different sizes, packed with silica (particle size: 40-63 µm) purchased from Macherey-Nagel as stationary phase. Flash column chromatography was performed using an Interchim PuriFlash 420 with prepacked columns (PF-30SHIP-F0012, -F0025, -F0040). Gel permeation chromatography was carried out using a JAI chromatography system (LC-5060, LaboACE) equipped three preparative columns in series using chloroform (HPLC grade) as the eluent at a flow rate of 6.5 mL min^{-1}.

UV/vis Absorption. UV/vis spectra were measured using a Jasco V-770 spectrophotometer with a PAC-743R Peltier cooling system and the Light induced aggregation studies were performed using an Agilent Technologies Cary 3500 Series UV/vis spectrophotometer (detailed in Section 6). Measurements employed 10 mm quartz fluorescence cuvettes from Hellma, with a stirring bead included. All the measurements were performed at 20 °C (293 K) unless otherwise stated. All solutions were prepared from dry, degassed, spectroscopy-grade solvents. Prior to sample analysis, a background spectrum of the pure solvent and the solvent mixture were recorded and subtracted from the sample measurements using Origin software. Solid state UV/vis measurements were recorded using JASCO V770 spectrophotometer equipped with a 60 mm integrating sphere (ISN-923). Thin films for the measurements were prepared by drop-casting the sample onto quartz substrate from Hellma.

High-Resolution Mass Spectrometry (HR-MS). Electrospray ionisation (ESI) high-resolution mass spectrometry was recorded on a Bruker Daltonics microTOF focus instrument with a resolution of 18.000 FWHM. Samples were prepared in MeCN, and the instrument was run in positive mode.

NMR Spectroscopy. NMR spectra were recorded at 25 °C (298 K) using a Bruker Avance HD III 400 MHz spectrometer automatically tuned and matched to the correct operating frequencies. The ^{13}C NMR spectra are broad-band proton decoupled. TopSpin 4.2 and MestReNova 14.3.2 were used to apply phase and baseline corrections. ^{1}H and ^{13}C NMR spectra were referenced to the residual solvent peak, and the ^{19}F NMR spectra of organic molecules were referenced to hexafluorobenzene at –164.9 ppm. Signals are reported in terms of chemical shift (ppm) and coupling constants (Hz). Abbreviations for multiplicity are as follows: s, singlet; d, doublet; t, triplet; m, multiplet; br, broad; hept, heptet.

Quantum Yields of Photoswitching. A USB thermal power meter (ThorLabs PM16-401) was used to determine the photon fluxes from the LEDs employed in this work (as shown in Figure S1), following the procedure detailed by Crespi and co-workers.[1] The quantum yields of photoisomerization were determined following reported literature procedure, with calculations performed using the python scripts developed by Crespi and co-workers.

CCD Spectrometer Setup. The CCD spectrometer setup schematically shown in Figure S1 was employed for photostationary state (PSS) and quantum yield measurements. The setup consists of an Ocean Insight DH-2000-FHS-DUV-TTL light source (190-2500 nm), connected to a Quantum Northwest QPOD sample holder by premium fibre optics (Ocean Insight QP400-025-SR-BX). The temperature of the QPOD sample holder was controlled with a Quantum Northwest TC 125 temperature controller, which also controlled the stirring. An Ocean Insight Flame-S-XR1-ES CCD array spectrometer was used to measure the absorbance spectrum of the sample. Pin-hole slits were employed, as shown in the schematic (Figure S1), along with an optical filter at the light source to reduce the probe light intensity (Thermo Oriel 50550). LEDs of various wavelengths (see Table S1 below) are employed to induce photoisomerization. These LEDs were fitted with adjustable collimation adapters supplied by ThorLabs (either an SM1U with an LA4052-UV Fused Silica Plano Convex Lens with an anti-reflective coating 245-400 nm or an SM1U25-A with an anti-reflective coating 350-700 nm, depending on the wavelength of the LED used). The LEDs were operated at a constant current mode controlled by a ThorLabs DC2200 LED driver.

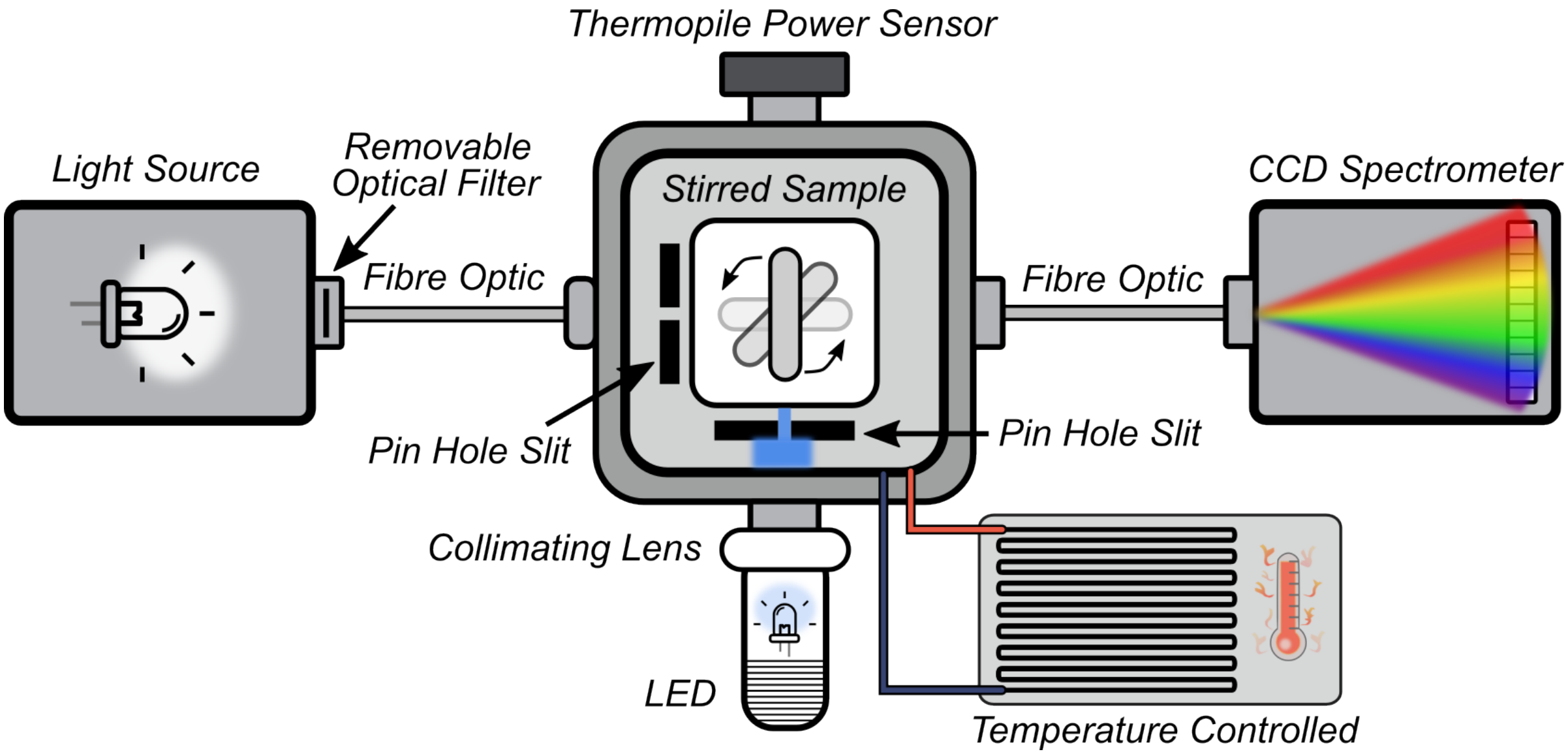

Figure S1. Schematic representation of the CCD spectrometer setup to measure the UV/vis absorption spectra and to perform photoswitching studies.

Table S1. Table displaying the models of ThorLabs mounted LEDs used in this study, the nominal emission wavelength (the wavelength in which emission appears brightest to the human eye), and the bandwidth (full width at half maximum, FWHM).

LED model number (ThorLabs)	Wavelength (nm)	Bandwidth/FWHM (nm)
M340L5	340	10
M365L3	365	9
M385L3	385	11
M405L4	405	13
M430L5	430	17
M470L5	470	28
M490L4	490	26
M505L4	505	37
M530L4	530	35

Melting points: Melting points were determined using an Olympus BX41 polarization microscope connected to a TP84 Linkam scientific temperature regulator.

Dynamic Light Scattering (DLS): Dynamic light scattering (DLS) measurements were conducted using a ZetaSizer Nano S and ZetaSizer Pro instrument (Malvern Instruments Ltd., Worcestershire, UK). The mean solvodynamic diameters reported are an average from 5 independent measurements. Measurements were performed at a temperature of 20 °C (293 K) and a detection angle of 173°.

Atomic Force Microscopy (AFM). AFM measurements were performed under ambient conditions using a Bruker Multimode 8 SPM system operating in tapping mode in air. Silicon cantilevers (OMCL-AC200TS, Olympus) with a resonance frequency of ~150 kHz and a spring constant of ~9 N m^{-1} were used. The AFM sample was prepared by drop-casting the samples in H_2O/MeCN (4:1, *v/v*) onto silicon wafer (SiO_x).

2. Synthesis and Characterization

A schematic overview of the synthetic approach towards the synthesis of **1** is shown in Figures S2 - S4. 2-(4-bromobutyl)isoindoline-1,3-dione (**S1**), 2-(3-bromopropyl)isoindoline-1,3-dione (**S2**), 2-(10-bromodecyl)isoindoline-1,3-dione (**S3**), 2-(1-hydroxy-3,6,9,12,15,18-hexaoxaoctacosan-28-yl)isoindoline-1,3-dione (**S4**) and 28-amino-3,6,9,12,15,18-hexaoxaoctacosan-1-ol (**S5**) were synthesized according to literature known procedures[2-4] and each synthetic scheme of compounds are shown on the corresponding sections.

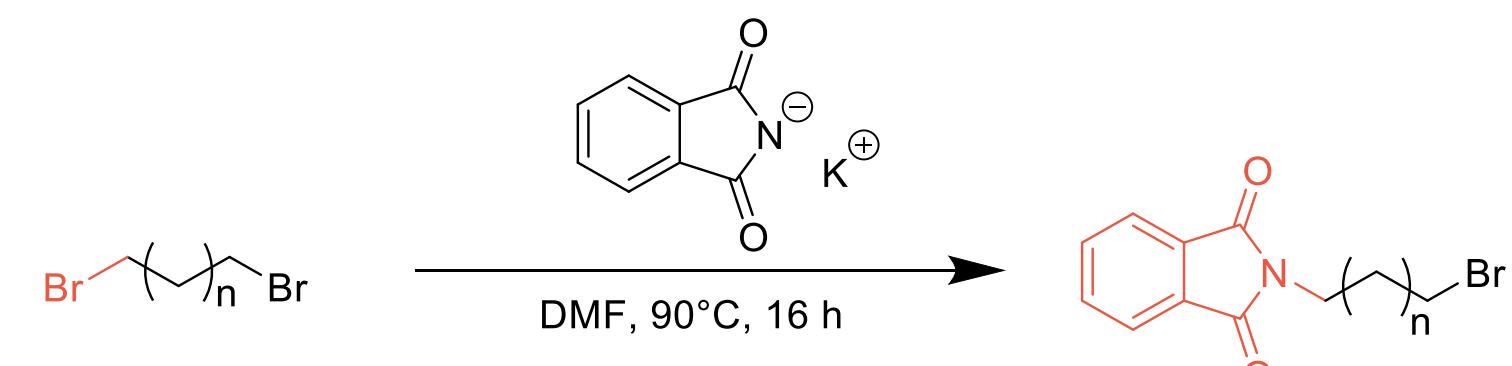

n = 1,2,8

S1 = n = 2
S2 = n = 1
S3 = n = 8

Figure S2. Synthetic scheme of hydrocarbon linkers.

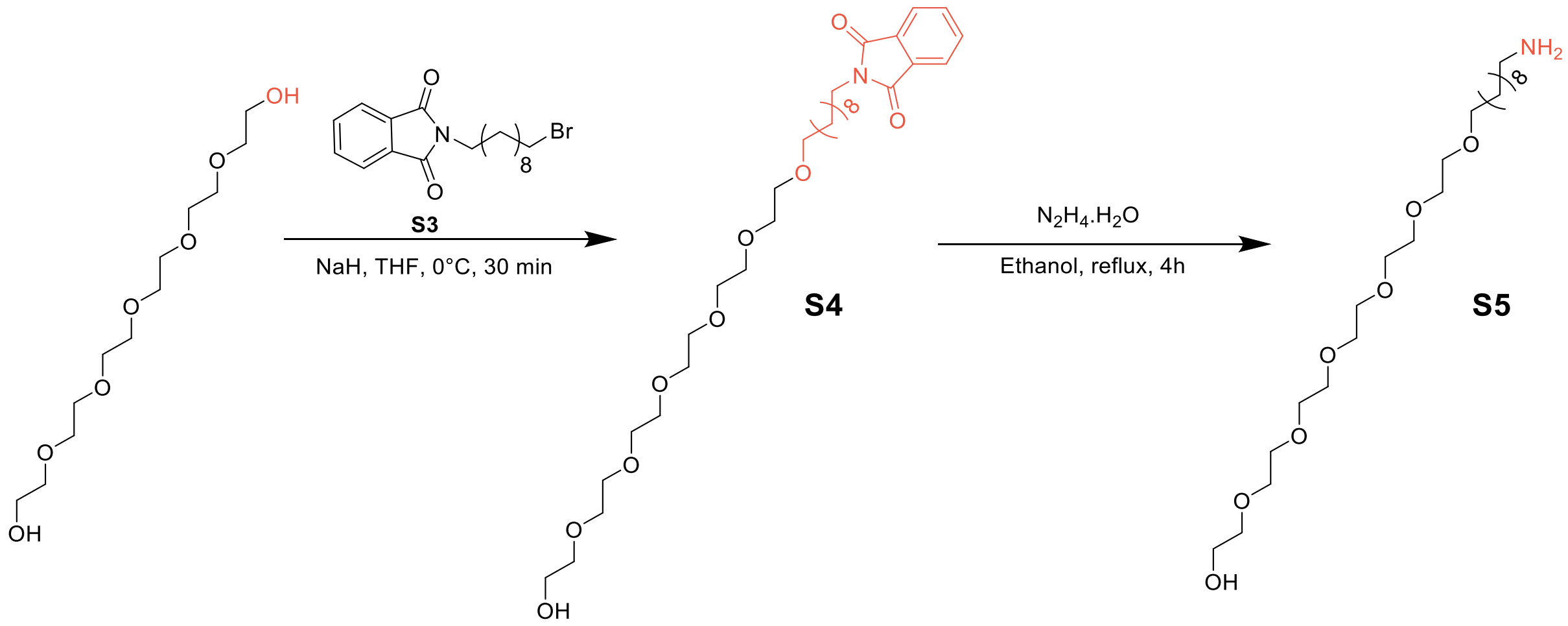

Figure S3. Synthetic scheme of sidechains.

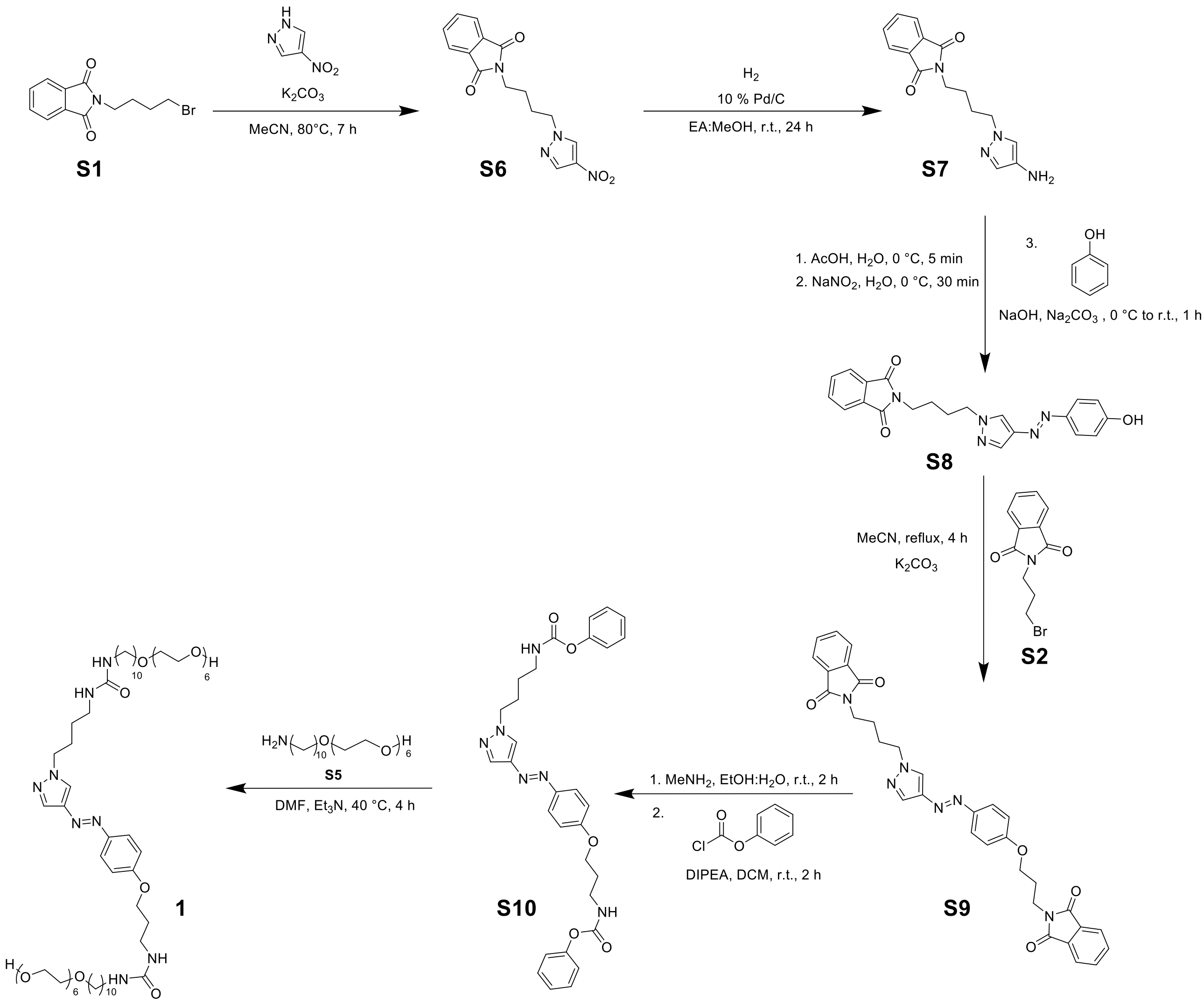

Figure S4. Synthetic scheme of **1**.

2.1. Synthesis of Hydrocarbon Linkers

Synthesis of 2-(4-bromobutyl)isoindoline-1,3-dione, S1

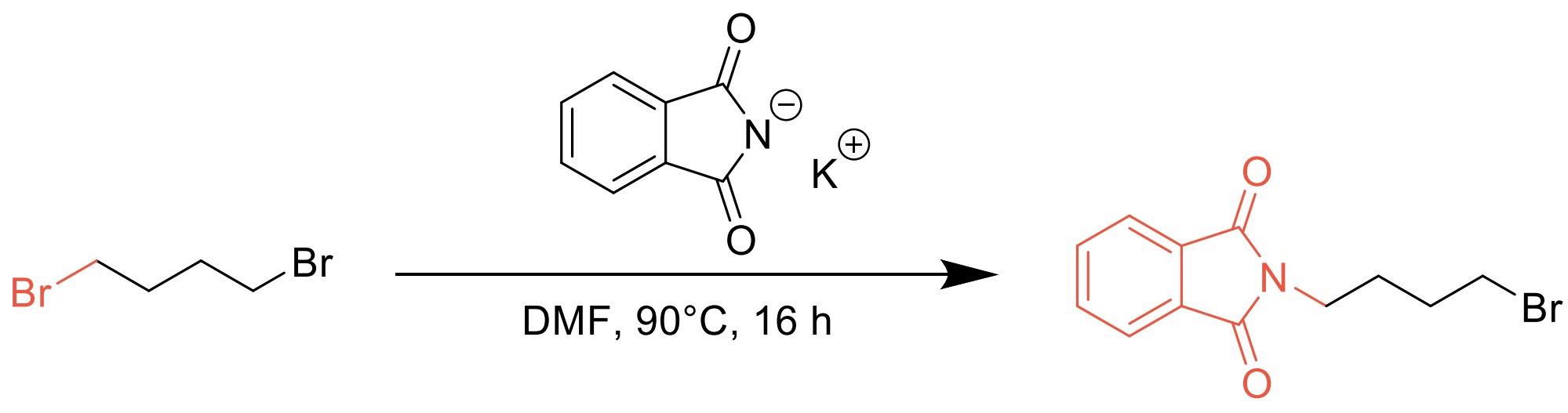

S1

To a stirred solution of 1,4-Dibromobutane (10.72 g, 49.7 mmol, 1 eq.) in anhydrous DMF (80 mL) was added potassium phthalimide (2.30 g, 12.4 mmol, 0.25 eq.). The reaction mixture was heated at 90 °C for 16 hours under N_2 atmosphere and then cooled to room temperature and filtered under gravity. The crude product was dissolved in DCM and purified by column chromatography with n-Hexane/EA (0% EA to 20% EA) to yield **S1** as white crystals (3.12 g, 11.1 mmol, 89%). ^{1}H NMR (400 MHz, 298 K, $CDCl_3$) δ 7.86 - 7.81 (m, 2H), 7.73 - 7.68 (m, 2H), 3.71 (t, J = 6.7 Hz, 2H), 3.43 (t, J = 6.4 Hz, 2H) 1.93 - 1.80 (m, 4H) ppm; ^{13}C NMR (101 MHz, 298 K, $CDCl_3$) δ 168.4, 134.0, 132.0, 123.3, 37.0, 32.8, 29.8, 27.3 ppm; HRMS (ESI-TOF, positive mode, MeCN/$CHCl_3$): m/z calculated: for $C_{12}H_{12}BrNO_2$ $[M+Na]^+$: 303.9944; found: 303.9943; mass error: −0.32 ppm.

Melting point: 81 - 82 °C

Synthesis of 2-(3-bromopropyl)isoindoline-1,3-dione, S2

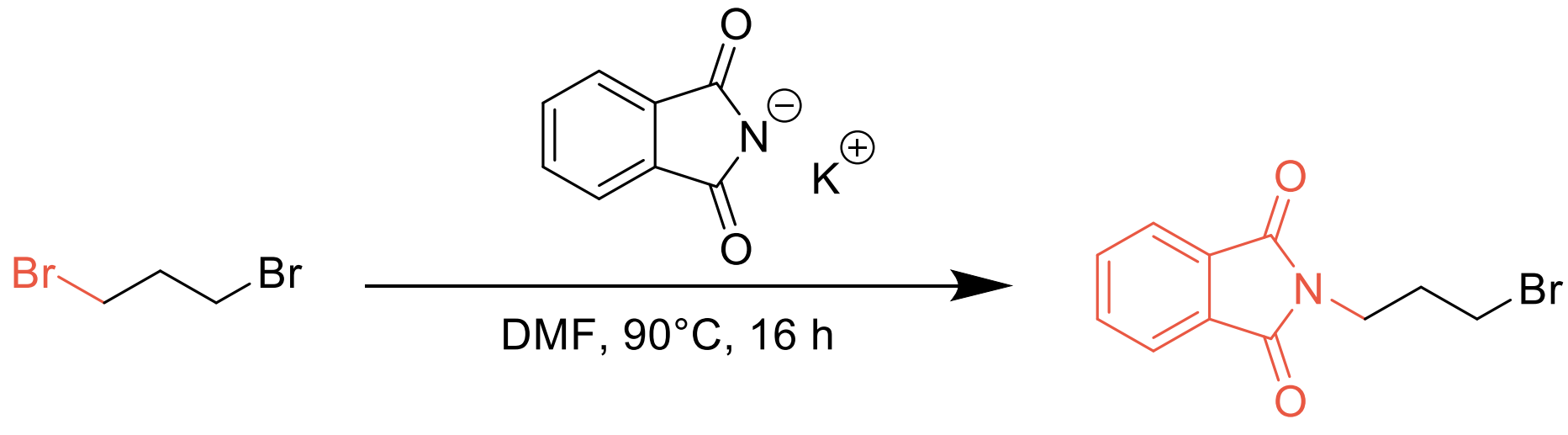

S2

To a stirred solution of 1,3-Dibromopropane (8.72 g, 4.40 mL, 43.2 mmol, 1 eq.) in anhydrous DMF (70 mL) was added potassium phthalimide (2.00 g, 10.8 mmol, 0.25 eq.). The reaction mixture was heated at 90 °C for 16 hours under N_2 atmosphere, cooled to room temperature and filtered under gravity. The crude product was dissolved in DCM and purified by column chromatography with n-Hexane/EA (0 %EA to 30% EA) to yield **S2** as white crystals (2.56 g, 9.5 mmol, 88%). ^{1}H NMR (400 MHz, 298 K, $CDCl_3$) δ 7.88 - 7.83 (m, 2H), 7.75 - 7.70 (m, 2H), 3.84 (t, J = 6.8 Hz, 2H), 3.42 (t, J = 6.7 Hz, 2H), 2.27 (p, J = 6.8 Hz, 2H) ppm; ^{13}C NMR (101 MHz, 298 K, $CDCl_3$) δ 168.3, 134.1, 132.0, 123.3, 36.7, 31.7, 29.8 ppm; HRMS (ESI-TOF, positive mode, MeCN/$CHCl_3$): m/z calculated: for $C_{11}H_{10}BrNO_2$ $[M+Na]^+$: 289.9787; found: 289.9796; mass error: 3.10 ppm.

Melting point: 74 - 76 °C

Synthesis of 2-(10-bromodecyl)isoindoline-1,3-dione, S3

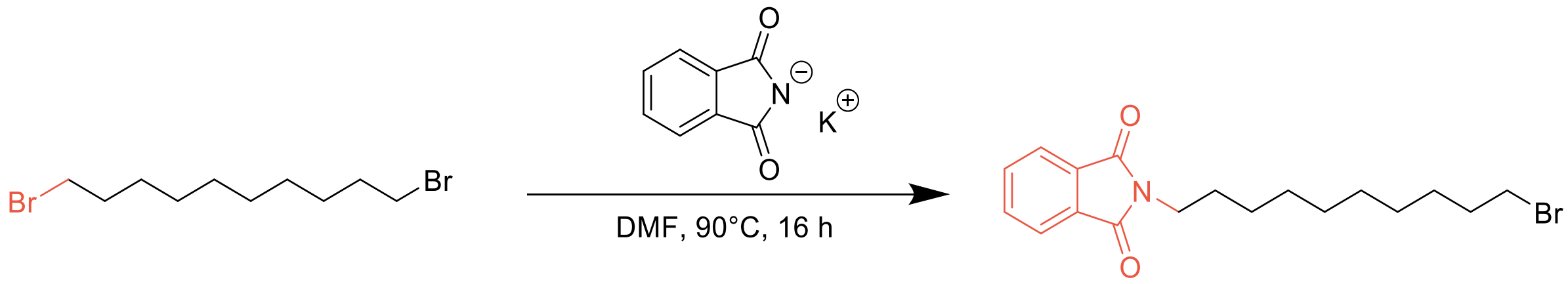

S3

To a stirred solution of 1,10-Dibromodecane (6.48 g, 21.6 mmol, 1 eq.) in anhydrous DMF (35 mL) potassium phthalimide added (1.00 g, 5.4 mmol, 0.25 eq.). The reaction mixture was heated at 90 °C for 16 hours under N_2 atmosphere, cooled to room temperature and filtered under gravity. The crude product was dissolved in DCM and purified by column chromatography with n-Hexane/EA (0% EA to 20%EA) to yield **S3** as white crystals (1.08 g, 2.95 mmol, 55%). ^{1}H NMR (400 MHz, 298 K, $CDCl_3$) δ 7.85-7.81 (m, 2H), 7.73-7.68 (m, 2H), 3.69-3.65 (m, 2H), 3.39 (t, J = 6.9 Hz, 2H), 1.84 (dt, J = 14.7, 6.9 Hz, 2H), 1.67 (p, J = 7.4 Hz, 2H), 1.40 (p, J = 6.9 Hz, 2H), 1.35-1.27 (m, 10H) ppm; ^{13}C NMR (101 MHz, 298 K, $CDCl_3$) δ 168.5, 133.8, 132.2, 123.2, 38.1, 34.0, 32.8, 29.3, 29.3, 29.1, 28.7, 28.6, 28.1, 26.8 ppm; HRMS (ESI-TOF, positive mode, MeCN/$CHCl_3$): m/z calculated: for $C_{18}H_{24}BrNO_2$ $[M+Na]^+$: 388.0883; found: 388.0891; mass error: 2.06 ppm.

Melting point: 58 - 60 °C

2.2. Synthesis of the Sidechain

Synthesis of 2-(1-hydroxy-3,6,9,12,15,18-hexaoxaoctacosan-28-yl)isoindoline-1,3-dione, S4

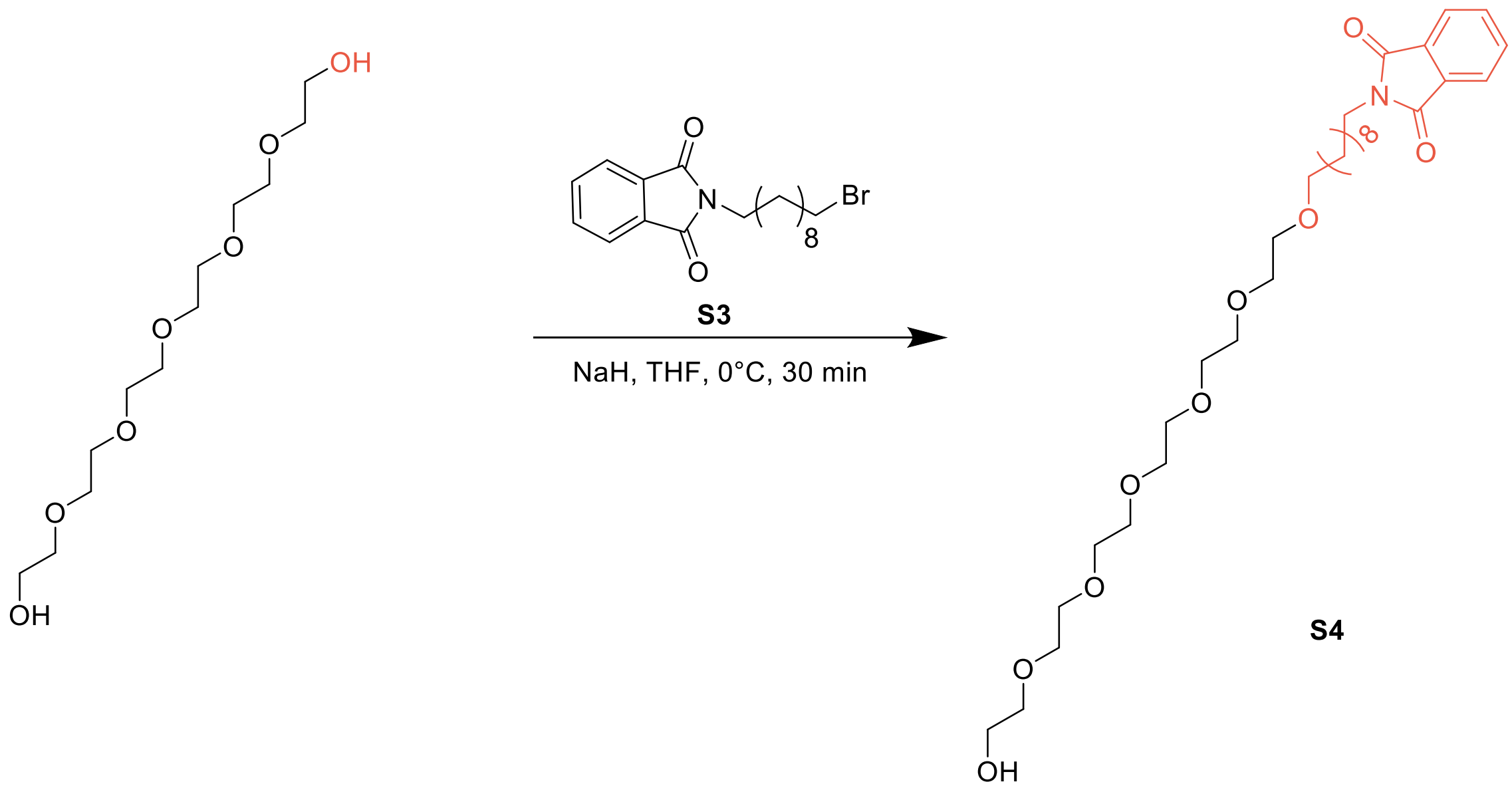

Hexaethylene glycol (2.90 g, 10.3 mmol, 1 eq.) was dried under high vacuum and dissolved in dry THF (30 mL). The solution was cooled to 0 °C and NaH (60% in mineral oil, 410.83 mg, 10.3 mmol, 1 eq.) was added upon which the solution started foaming. The reaction mixture was stirred for 30 minutes under N_2 atmosphere and when no further foam generation was observed, **S3** (3.76 g, 10.3 mmol, 1 eq.) was added. The ice bath was removed, and the reaction mixture was stirred overnight at RT. The solution was neutralised with water (30 mL) and extracted with diethyl ether (3×40 mL). The combined organic layers were dried over anhydrous magnesium sulfate, filtered, and concentrated under vacuum. The crude product was dissolved in DCM, and a vacuum filtration was carried out. First, DCM was added several times to remove leftover **S3**. The completion of this step was monitored by TLC. Once all **S3** had been removed, a mixture of DCM:MeOH (9:1) was used to obtain the product. **S4** was obtained as a colourless oil (1.55 g, 2.7 mmol, 26%).^{1}H NMR (400 MHz, 298 K, $CDCl_3$): δ 7.85-7.81 (m, 2H), 7.73-7.68 (m, 2H), 3.73-3.60 (m, 24H), 3.58-3.55 (m, 2H), 3.43 (t, J = 6.8 Hz, 2H), 1.70-1.63 (m, 2H), 1.59-1.52 (m, 2H), 1.33-1.25 (m, 12H) ppm; ^{13}C NMR (101 MHz, 298 K, $CDCl_3$): δ 168.5, 133.8, 132.2, 123.2, 72.5, 71.5, 70.6, 70.6, 70.6, 70.3, 70.1, 61.8, 38.1, 29.6, 29.5, 29.4, 29.2, 28.6, 26.9, 26.1 ppm; HRMS (ESI-TOF, positive mode, MeCN/$CHCl_3$): m/z calculated: for $C_{30}H_{49}NO_9$ $[M+H]^+$: 568.3480; found: 568.3506; mass error: 4.57 ppm; $[M+Na]^+$: 590.3300; found: 590.3328; mass error: 4.74 ppm.

Synthesis of 28-amino-3,6,9,12,15,18-hexaoxaoctacosan-1-ol, S5

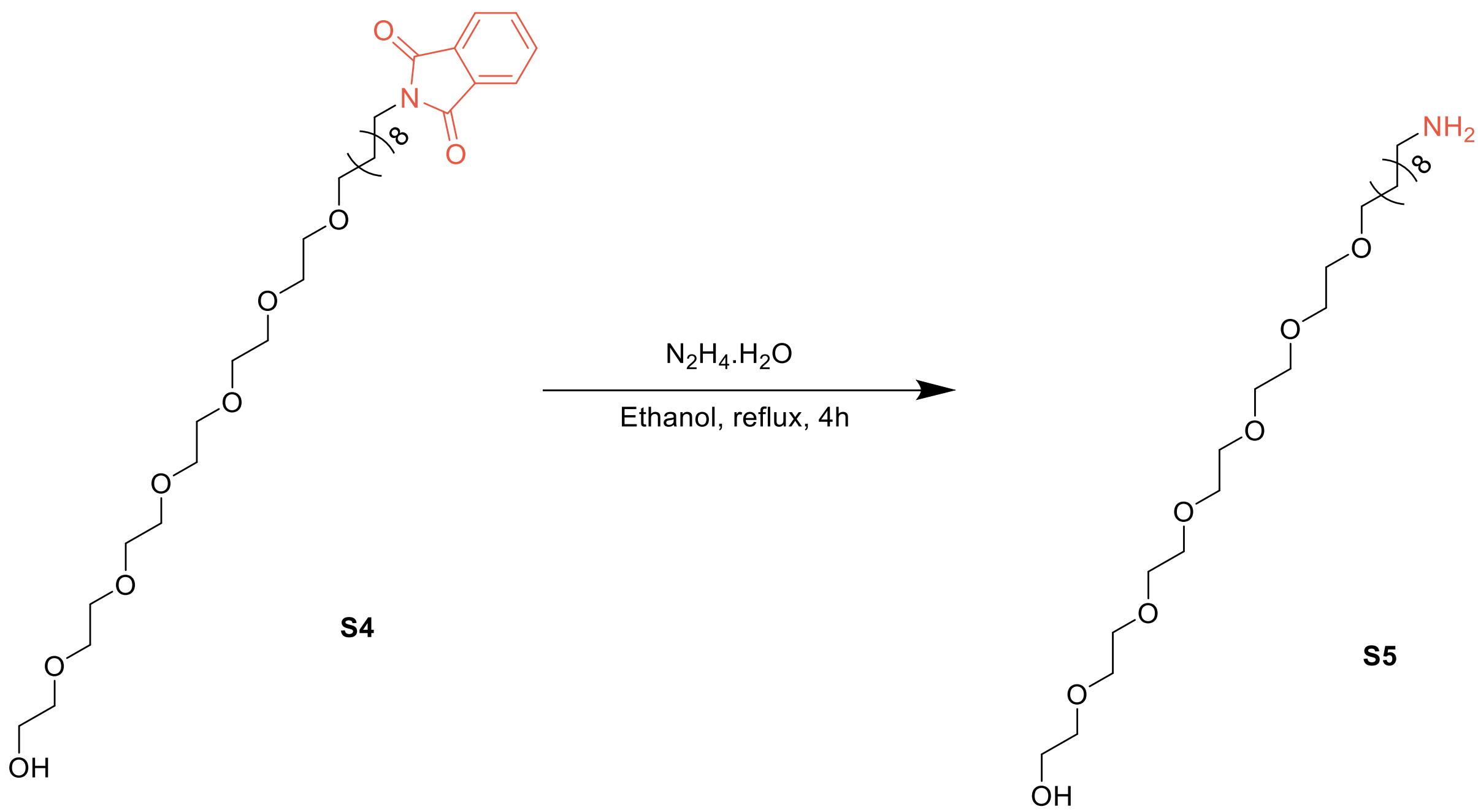

Based on a procedure reported by Feringa and co-workers,[2] **S4** (2.00 g, 3.5 mmol, 1 eq.) was dissolved in EtOH (40 mL) and hydrazine hydrate (1.75 mL, 35.2 mmol, 10 eq.) was added. The reaction mixture was heated at reflux for 4 hours, cooled to room temperature, and dissolved in 15% aq. NaOH (100 mL). After extracting with DCM, the combined organic layers were washed with brine, dried over Na_2SO_4, and concentrated under vacuum. The crude product was purified by flash column chromatography (DCM:MeOH from 5% MeOH to 15% MeOH) to afford **S5** as a colourless oil (0.86 g, 2 mmol, 57%). ^{1}H NMR (400 MHz, 298 K, $CDCl_3$): δ 3.73 - 3.56 (m, 24H), 3.44 (t, J = 6.8 Hz, 2H), 2.69 - 2.66 (m, 2H), 1.60 - 1.53 (m, 2H), 1.45 - 1.40 (m, 2H), 1.29 - 1.25 (m, 12H) ppm; ^{13}C NMR (101 MHz, 298 K, $CDCl_3$): δ 72.6, 71.5, 70.6, 70.6, 70.6, 70.6, 70.3, 70.1, 61.7, 42.2, 33.7, 29.6, 29.5, 29.5, 29.5, 29.4, 26.9, 26.1 ppm; HRMS (ESI-TOF, positive mode, MeCN/$CHCl_3$): m/z calculated: for $C_{22}H_{47}NO_7$ $[M+H]^+$: 438.3425; found: 438.3446; mass error: 4.79 ppm; $[M+Na]^+$: 460.3245; found: 460.3267; mass error: 4.77 ppm.

2.3. Main Synthetic route

Synthesis of 2-(4-(4-nitro-1H-pyrazol-1-yl)butyl)isoindoline-1,3-dione, S6

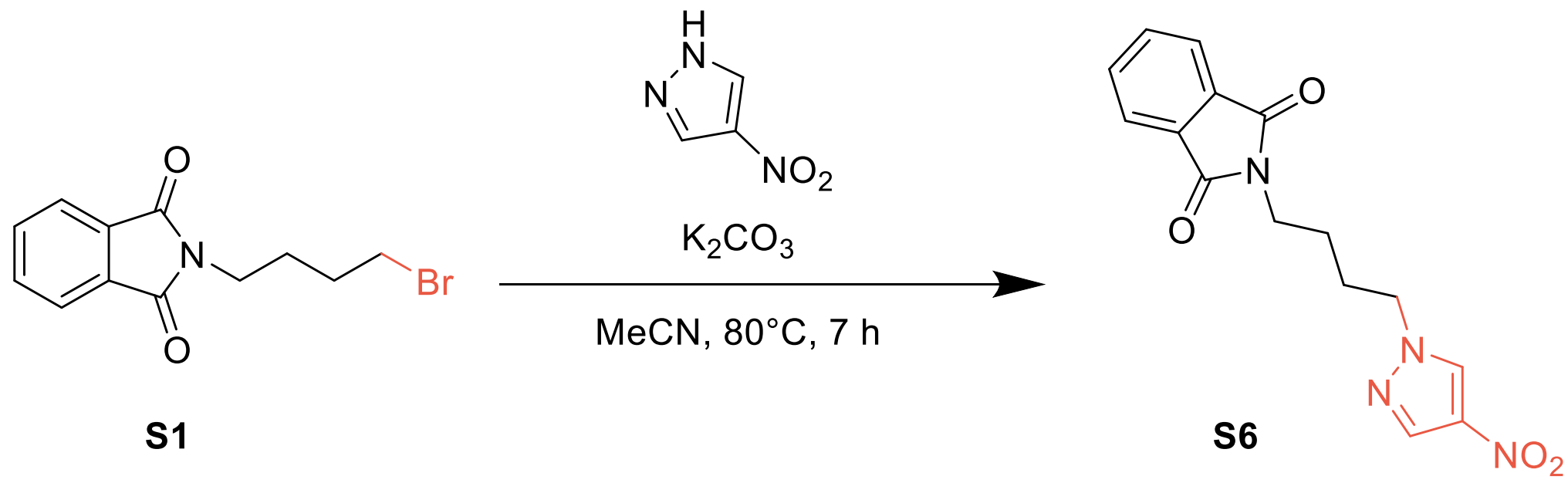

Based on a procedure reported by Fuchter,[5] **S1** (2.30 g, 8.2 mmol, 1 eq.), 4-nitro-1H-pyrazole (1.11 g, 9.8 mmol, 1.2 eq.) and K_2CO_3 (2.48 g, 17.9 mmol, 2.2 eq.) and dry MeCN (10 mL) were added to a dried flask equipped with a condenser. The reaction mixture was stirred at 80 °C for 7 hours under a N_2 atmosphere. After cooling to room temperature, the reaction mixture was filtered under gravity and concentrated under vacuum. Purification by column chromatography using cyclohexane and ethyl acetate mixture (4:1 to 0:1) afforded **S6** as white crystals (2.44 g, 7.7 mmol, 94%). ^{1}H NMR (400 MHz, 298 K, $CDCl_3$): δ 8.14 (d, J = 0.7 Hz, 1H), 8.03 (d, J = 0.7 Hz, 1H), 7.86 - 7.82 (m, 2H), 7.75 - 7.69 (m, 2H), 4.21 (t, J = 7.1 Hz, 2H), 3.73 (t, J = 6.9 Hz, 2H), 1.95 (tt, J = 7.7, 6.4 Hz, 2H), 1.75 - 1.68 (m, 2H) ppm; ^{13}C NMR (101 MHz, 298 K, $CDCl_3$): δ 168.4, 135.9, 134.1, 132.0, 128.5, 123.4, 52.7, 36.8, 27.0, 25.4 ppm; HRMS (ESI-TOF, positive mode, MeCN/$CHCl_3$): m/z calculated: for $C_{15}H_{14}N_4O_4$ $[M+Na]^+$: 337.0907; found: 337.0896; mass error: −3.26 ppm.

Synthesis of 2-(4-(4-amino-1H-pyrazol-1-yl)butyl)isoindoline-1,3-dione, S7

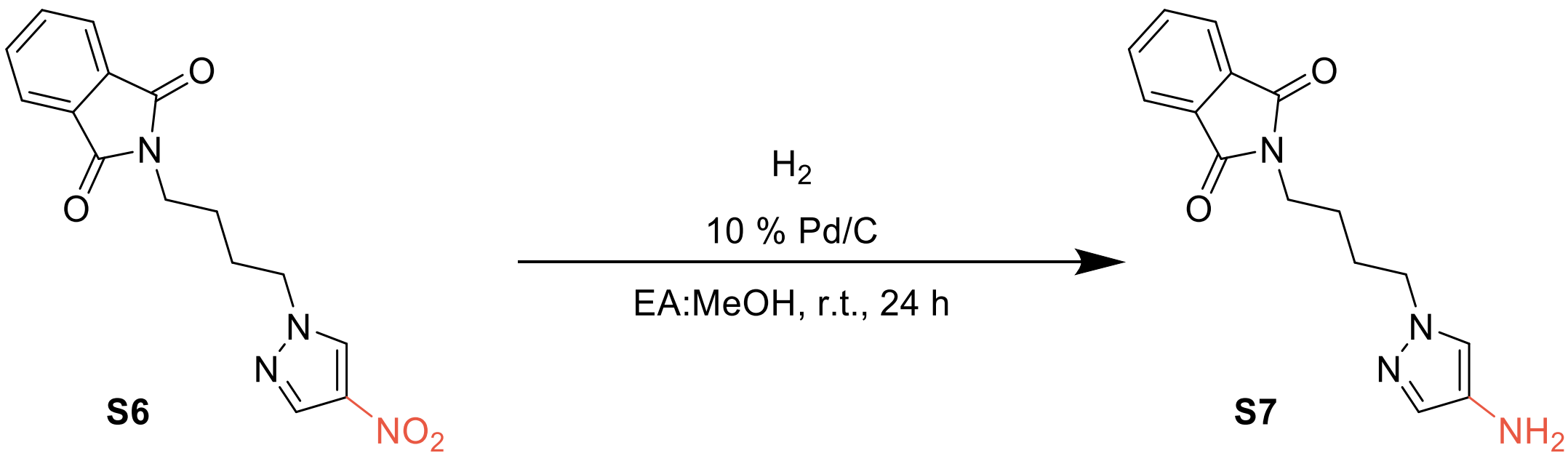

S6 (2.39 g, 7.6 mmol, 1 eq) was dissolved in 90 mL of EA and 15 mL of MeOH. The solution was degassed with N_2 for 15 minutes, then Pd/C (10 wt%, 239 mg) was added and the solution was degassed for another 15 minutes. Then H_2 was led through the stirred reaction mixture using a balloon and an exit needle. After 10 minutes, the exit needle was removed, and the mixture was stirred at room temperature for one day. The progress of the reaction was monitored by TLC (cyclohexane:EA, 1:1). When completion was reached, the reaction mixture was degassed with N_2 for 15 minutes and filtered through celite with vacuum. A mixture of 50 mL of EA and 10 mL MeOH was used to wash all compounds out of the celite. The resulting solution was concentrated under vacuum to afford **S7** as a yellow oil which was used without further purification (2.17 g, 7.6 mmol, quantitative). ^{1}H NMR (400 MHz, 298 K, $CDCl_3$): δ 7.83 - 7.79 (m, 2H), 7.72 - 7.68 (m, 2H), 7.11 (d, J = 1.0 Hz, 1H), 6.99 (d, J = 0.9 Hz, 1H), 4.02 (t, J = 6.9 Hz, 2H), 3.68 (t, J = 7.0 Hz, 2H), 2.87 (s, 2H), 1.87 - 1.8 (m, 2H), 1.69 - 1.61 (m, 2H) ppm; ^{13}C NMR (101 MHz, 298 K, $CDCl_3$): δ 168.4, 134.0, 134.0, 132.1, 131.2, 128.7, 123.3 123.3, 118.5, 51.5, 37.2, 27.7, 25.7 ppm**;** HRMS (ESI-TOF, positive mode, MeCN/$CHCl_3$): m/z calculated: for $C_{15}H_{16}N_4O_2$ $[M+H]^+$: 285.1346; found: 285.1348, mass error: −0.70 ppm; $[M+Na]^+$: 307.1165; found: 307.1168; mass error: 0.97 ppm.

<u>Synthesis of (*E*)-2-(4-(4-((4-hydroxyphenyl)diazenyl)-1H-pyrazol-1-yl)butyl)isoindoline-1,3-dione, S8</u>

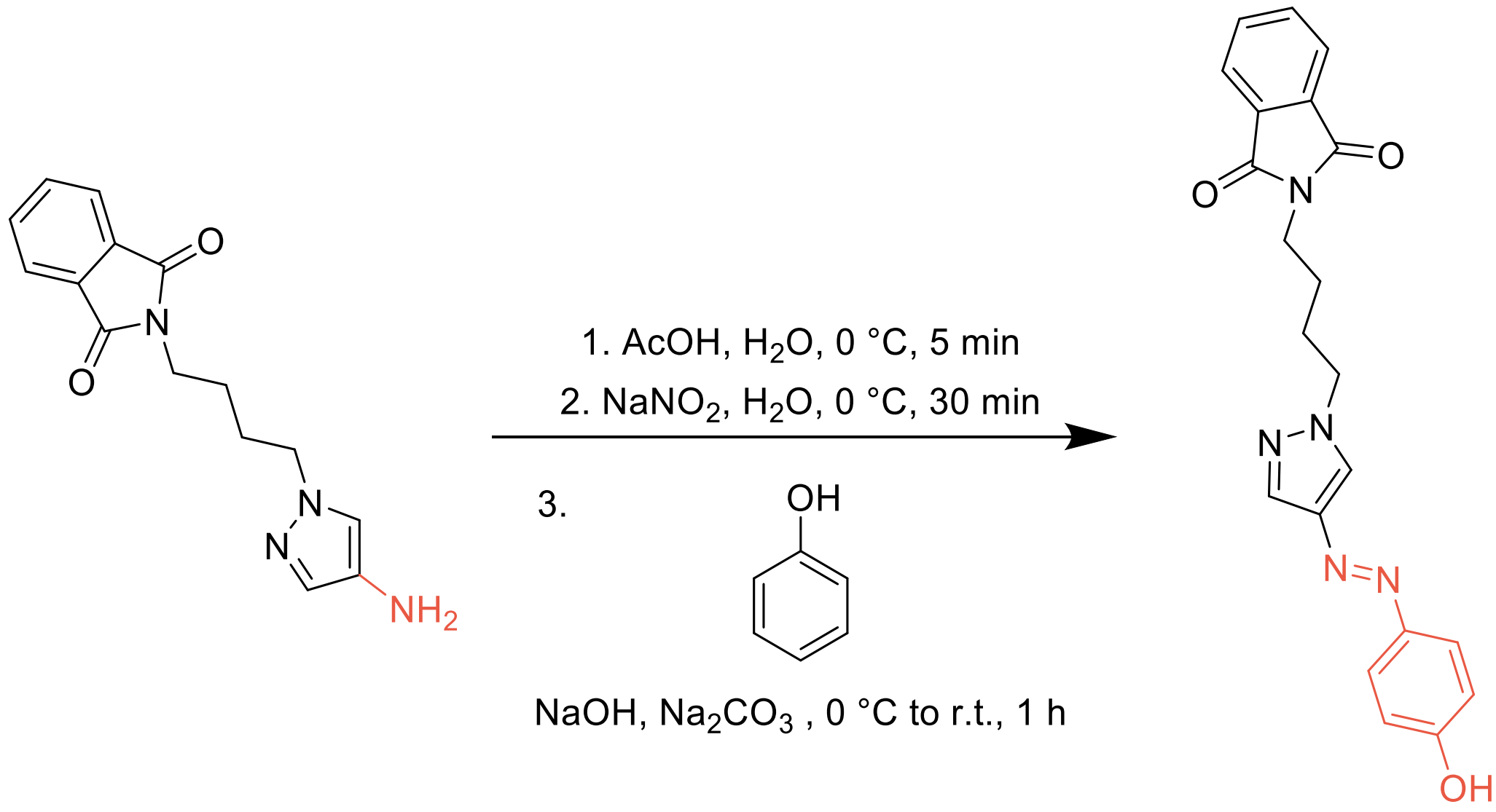

S7 **S8**

Based on a procedure reported by Fuchter,[5] **S7** (743 mg, 2.6 mmol, 1 eq.) was dissolved in acetic acid (2.25 mL, 39.3 mmol, 15 eq.), EtOH (4 mL) and water (10 mL) at 0 °C. After 5 min, a precooled solution of $NaNO_2$ (253 mg, 3.7 mmol, 1.4 eq.) in water (10 mL) was added and the reaction mixture was stirred for 30 minutes at 0 °C. In a separate flask, a phenol (295 mg, 3.1 mmol, 1.2 eq.), and NaOH (1.10 g, 27.5 mmol, 10.5 eq.) were dissolved in water (10 mL) and cooled to 0 °C. This solution was slowly added to the reaction mixture and immediately after, a precooled solution of Na_2CO_3 (2.49 g, 23.6 mmol, 9 eq.) in water (20 mL) was carefully added. The ice bath was removed, and the reaction mixture was stirred for 1 h, resulting in the precipitation of a yellow solid. The precipitate was collected by suction filtration and dried under high vacuum yielding a yellow solid which was used without further purification (762 mg, 2 mmol, 77%). ^{1}H NMR (400 MHz, 298 K, $CDCl_3$): δ 7.98 (s, 1H), 7.94 (s, 1H), 7.85 - 7.81 (m, 2H), 7.74 - 7.68 (m, 4H), 6.93 - 6.89 (m, 2H), 6.12 (s, 1H), 4.22 (t, J = 7.0 Hz, 2H), 3.74 (t, J = 7.0, 2H), 2.00 - 1.93 (m, 2H), 1.77 - 1.69 (m, 2H) ppm; ^{13}C NMR (101 MHz, 298 K, $CDCl_3$): δ 168.5, 158.0, 147.2, 141.5, 134.1, 133.3, 132.0, 125.5, 124.2, 123.3, 115.8, 52.1, 37.1, 27.4, 25.6 ppm; HRMS (ESI-TOF, positive mode, MeCN/$CHCl_3$): m/z calculated: for $C_{21}H_{19}N_5O_3$ $[M+H]^+$: 390.1561; found: 390.1547; mass error: −3.58 ppm; $[M+Na]^+$: 412.1380; found: 412.1374; mass error: −1.45 ppm.

Synthesis of (*E*)2-(3-(4-((1-(4-(1,3-dioxoisoindolin-2-yl)butyl)-1H-pyrazol-4-yl)diazenyl)phenoxy)propyl)isoindoline-1,3-dione, S9

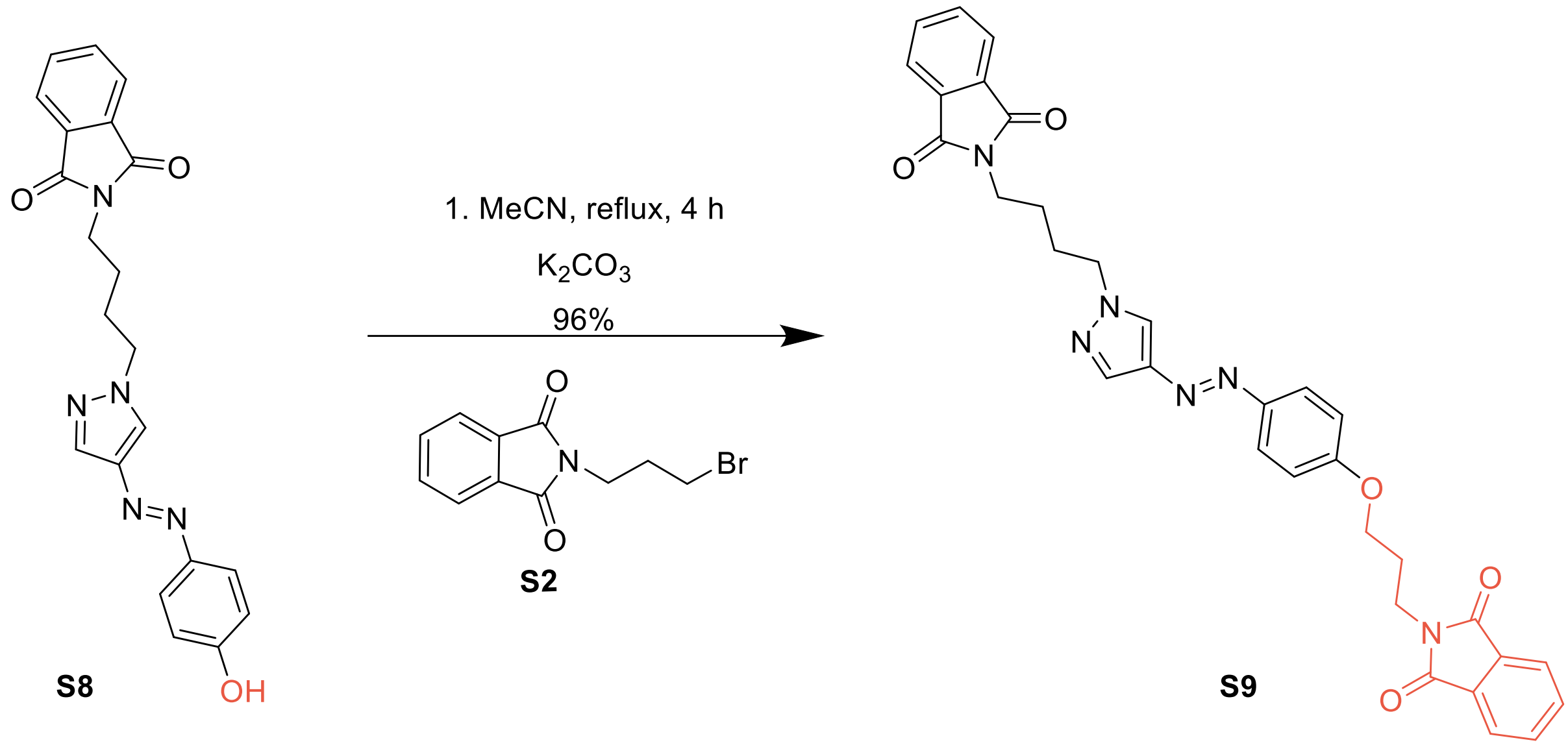

In a dried flask, **S8** (1.40 g, 3.6 mmol, 1 eq.), **S2** (1.45 g, 5.4 mmol, 1.5 eq.), and K_2CO_3 (1.99 g, 14.4 mmol, 4 eq.) were combined and dissolved in dry MeCN (150 mL). The reaction mixture was stirred at reflux under N_2 atmosphere for 4 hours. Subsequently, excess K_2CO_3 was removed by gravity filtration. The remaining solution was concentrated under vacuum and purified by flash column chromatography using DCM with MeOH (0 - 2%) to afford **S9** as white solid (1.99 g, 3.5 mmol, 96%). ^{1}H NMR (400 MHz, 298 K, $CDCl_3$): δ 7.94 (d, J = 0.6 Hz, 1H), 7.93 (d, J = 0.6 Hz, 1H), 7.83 - 7.81 (m, 4H), 7.71 - 7.68 (m, 6H), 6.86 - 6.82 (m, 2H), 4.20 (t, J = 7.0 Hz 2H), 4.08 (t, J = 5.8 Hz, 2H), 3.91 (t, J = 6.8 Hz, 2H), 3.72 (t, J = 7.0 Hz, 2H), 2.20 (p, J = 6.4 Hz, 2H), 1.97 - 1.91 (m, 2H), 1.75 - 1.68 (m, 2H) ppm; ^{13}C NMR (101 MHz, 298 K, $CDCl_3$): δ 168.4, 160.5, 147.2, 141.6, 134.0, 134.0, 133.2,132.1, 132.0, 125.5, 123.9, 123.3, 114.6, 65.9, 60.4, 52.1, 37.1, 35.4, 28.3, 27.4, 25.6, 21.1, 14.22 ppm; HRMS (ESI-TOF, positive mode, MeCN/$CHCl_3$): m/z calculated: for $C_{32}H_{28}N_6O_5$ $[M+H]^+$: 577.2194; found: 577.2212; mass error: 3.11 ppm; $[M+Na]^+$: 599.2013; found: 599.2038; mass error: 4.17 ppm.

Synthesis of phenyl (*E*)-(3-(4-((1-(4-((2-oxo-2-phenoxyethyl)amino)butyl)-1H-pyrazol-4-yl)diazenyl)phenoxy)propyl)glycinate, S10

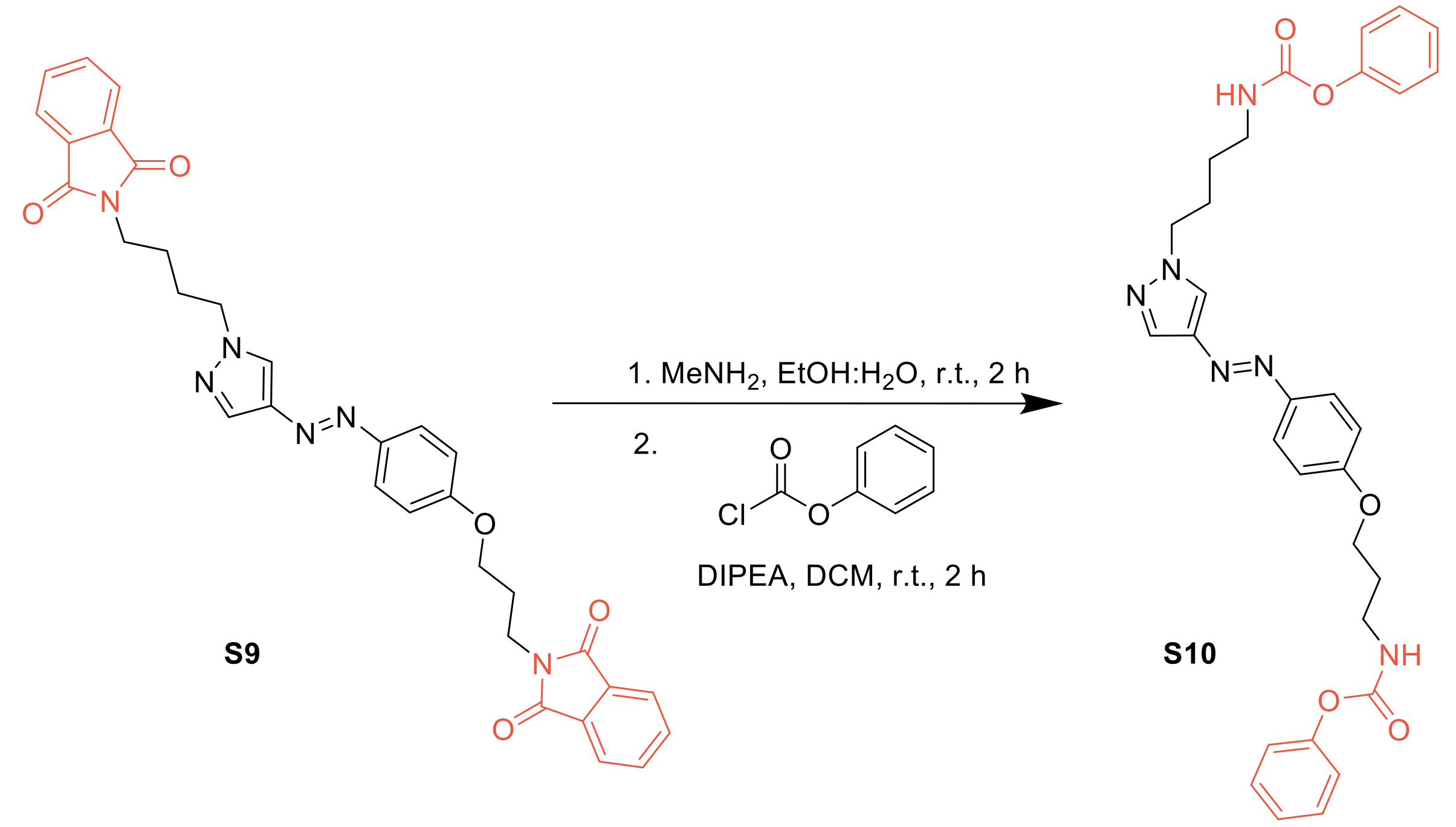

S9 (1.96 g, 3.4 mmol, 1 eq.) was dissolved in a solution of methylamine in EtOH (33%, 30 mL) and water (30 mL) was added. The reaction mixture was stirred at room temperature for 2 hours. The progress of the reaction was monitored by TLC (Et_2O). Once the step was completed, the solvent and excess methylamine were evaporated under vacuum. The remaining solid was dried in the same flask under high vacuum overnight. Then, the flask was equipped with a condenser, N_2 atmosphere was applied, and the flask was cooled to 0 °C. In a separate flask, phenyl chloroformate (1.29 mL, 10.2 mmol, 3 eq.) and N, N-diisopropylethylamine (1.76 mL, 10.2 mmol, 3 eq.) were added to dry DCM (40 mL) under nitrogen atmosphere at 0 °C (273.15 K). The precooled solution was added to the flask containing the intermediate compound and the reaction mixture was stirred for 5 min after which the ice bath was removed, and the reaction mixture was stirred at room temperature overnight. Subsequently, the reaction mixture was concentrated under vacuum, and the crude product was purified by flash column chromatography (n-hexane:EA, 1:0 to 1:3) to afford product **S10** as orange solid (1.37 g, 2.5 mmol, 73%). ^1H NMR (400 MHz, 298 K, $CDCl_3$)**:** δ 8.01 (s, 1H), 7.93 (s, 1H), 7.80 - 7.77 (m, 2H), 7.37 - 7.31 (m, 4H), 7.20 - 7.15 (m, 2H), 7.13 - 7.10 (m, 4H), 6.98 - 6.94 (m, 2H), 5.57 (t, J = 5.9 Hz, 1H), 5.41 (t, J = 6.1 Hz, 1H), 4.16 - 4.12 (m, 4H), 3.45 (q, J = 6.4 Hz, 2H), 3.23 (q, J = 6.7 Hz, 2H), 2.08 - 2.00 (m, 2H),

1.97 - 1.90 (m, 2H), 1.56 - 1.52 (m, 2H) ppm; ^{13}C NMR (101 MHz, 298 K, $CDCl_3$): δ 160.6, 154.8, 151.1, 151.1, 147.3, 141.6, 133.3, 129.3, 129.31, 125.6, 125.3, 125.3, 124.0, 121.6, 114.7, 66.1, 60.4, 52.3, 40.5, 38.7, 29.2, 27.3, 26.8, 21.1, 14.2 ppm; HRMS (ESI-TOF, positive mode, MeCN/$CHCl_3$): m/z calculated: for $C_{30}H_{32}N_6O_5$ $[M+Na]^+$: 579.2326; found: 579.2317; mass error: −1.57 ppm.

Synthesis of (E)-1-(5-(4-((4-((1-hydroxy-30-oxo-3,6,9,12,15,18-hexaoxa-29,31-diazapentatriacontan-35-yl)oxy)phenyl)diazenyl)-1H-pyrazol-1-yl)pentyl)-3-(1-hydroxy-3,6,9,12,15,18-hexaoxaoctacosan-28-yl)urea (1)

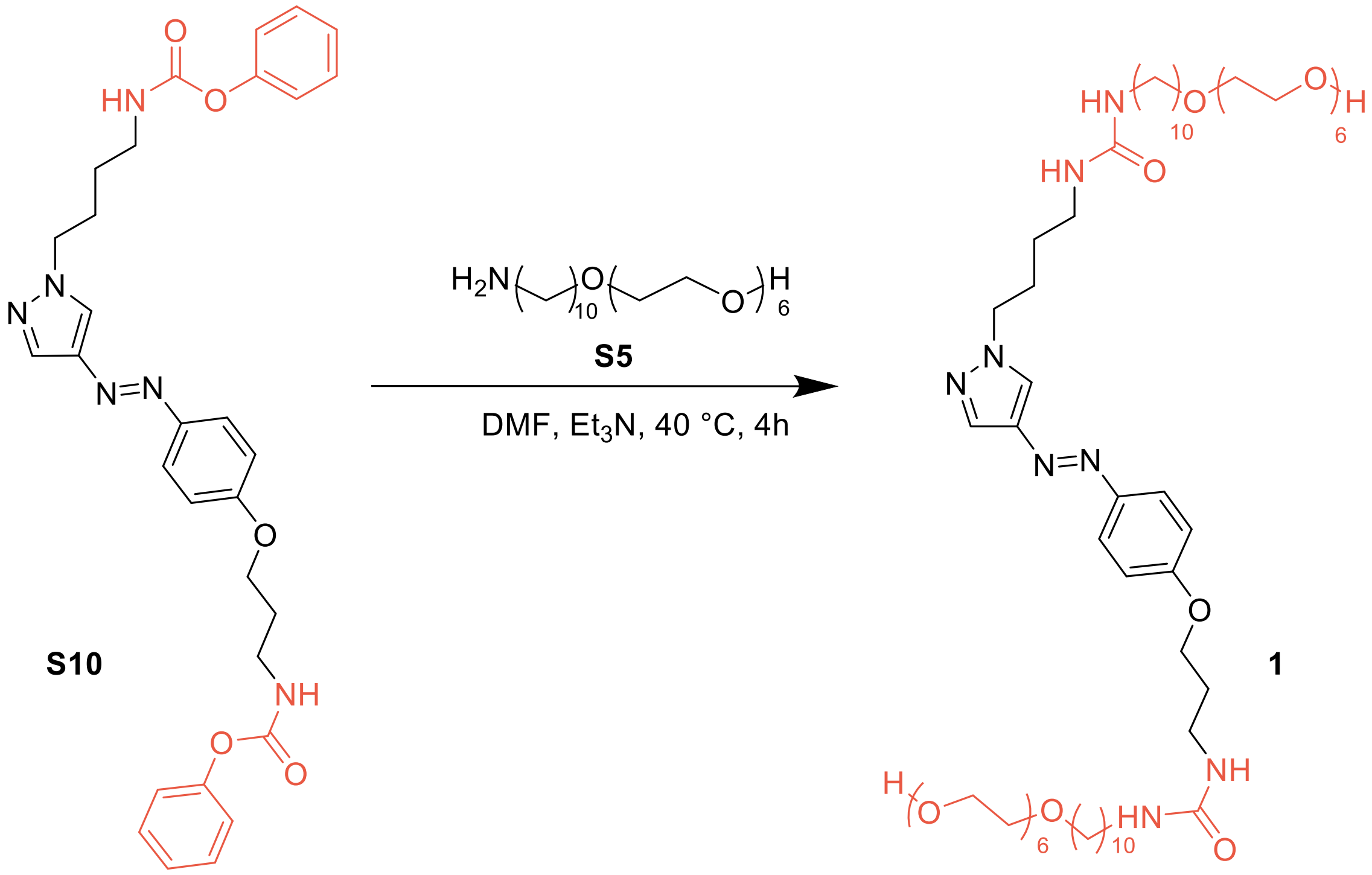

To a solution of **S10** (1.00 g, 1.8 mmol, 1 eq.) in dry DMF (40 mL) was added triethylamine (547.9 µL, 4.0 mmol, 2.2 eq.) and compound **S5** (1.73 g, 4.0 mmol, 2.2 eq.). The mixture was stirred at 40 °C for 4 hours. After cooling to room temperature, the solution was concentrated under vacuum. The resulting viscous oil was dissolved in a minimum amount of DCM, and the product was precipitated by adding pentane (8 mL). This process was repeated five times. After drying under vacuum, the solid was dissolved in a mixture of DCM and MeOH (20 % MeOH) and passed through celite. The solvent was removed under vacuum again, after which the compound was purified by gel permeation chromatography (LaboAce, $CHCl_3$:MeOH, 9:1) to yield **1** as a yellow solid (1.05 g, 0.8 mmol, 45 %). ^{1}H NMR (400 MHz, 298 K, DMSO-d6): δ 8.42 (d, J = 0.7 Hz, 1H), 7.92 (d, J = 0.7 Hz, 1H), 7.74 - 7.70 (m, 2H), 7.08 - 7.04 (m, 2H), 5.88 (t, J = 5.8 Hz, 1H), 5.79 - 5.76 (m, 2H), 5.72 (t, J = 5.7 Hz, 1H), 4.57 (t, J = 5.5 Hz, 2H), 4.17 (t, J = 7.0 Hz, 2H), 4.05 (t, J = 6.3 Hz, 2H), 3.50 - 3.39 (m, 48H), 3.36 - 3.31 (m, 4H), 3.17 - 3.12 (m, 2H), 3.02 - 2.91 (m, 6H), 1.87 - 1.75 (m, 4H), 1.47 - 1.44 (m, 4H), 1.32 - 1.31 (m, 6H), 1.23 - 1.22 (m, 24H) ppm; ^{13}C NMR (101 MHz, 298 K, DMSO-d6): δ 160.5, 158.1, 158.1, 146.4, 140.7, 131.9, 127.0, 123.5, 114.9, 72.3, 70.3, 69.8, 69.8,

69.8, 69.5, 65.8, 60.2, 51.6, 38.6, 36.2, 30.0, 29.7, 29.2, 29.0, 29.0, 28.9, 28.8, 28.8, 27.1, 26.4, 25.7 ppm; HRMS (MALDI-TOF, positive mode, MeCN): m/z calculated: for $C_{62}H_{114}N_8O_{17}$ $[M+H]^+$: 1243.8375; found: 1243.8328; mass error: 3.77 ppm.

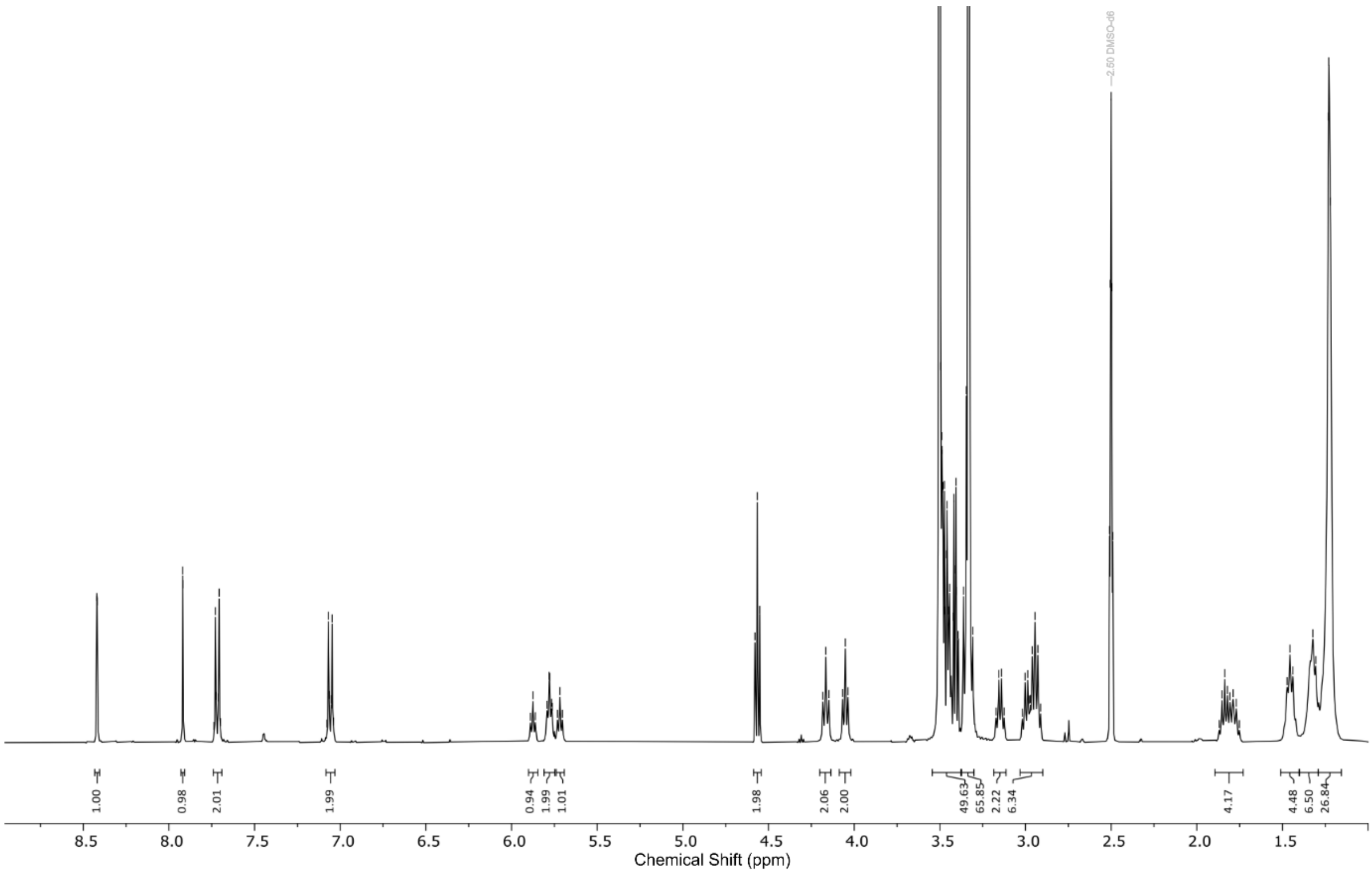

Figure S5. ^{1}H NMR (400 MHz, 298 K, DMSO-d6) of *E*-**1**.

3. UV/vis and Photoswitching Studies

3.1. Extinction Coefficient

To determine the molar extinction coefficient (ε) of **1**, five solutions of varying concentrations of **1** in DMSO were prepared (10, 20, 30, 40 and 50 μM), and their UV/vis spectra were recorded on Jasco V-770 UV/vis/NIR spectrophotometer. According to the Beer Lambert law, there exists a linear relationship between the absorbance and the concentration of the sample solution, which can be expressed as:

$$A = \varepsilon l c \qquad (1)$$

Where "A" is the absorbance of the sample in absorbance units (a.u.), "l" is the optical pathlength in cm, and "c" is the concentration of the sample solution in moles per litre (M) and "ε" is the molar extinction coefficient (M^{-1} cm^{-1}).

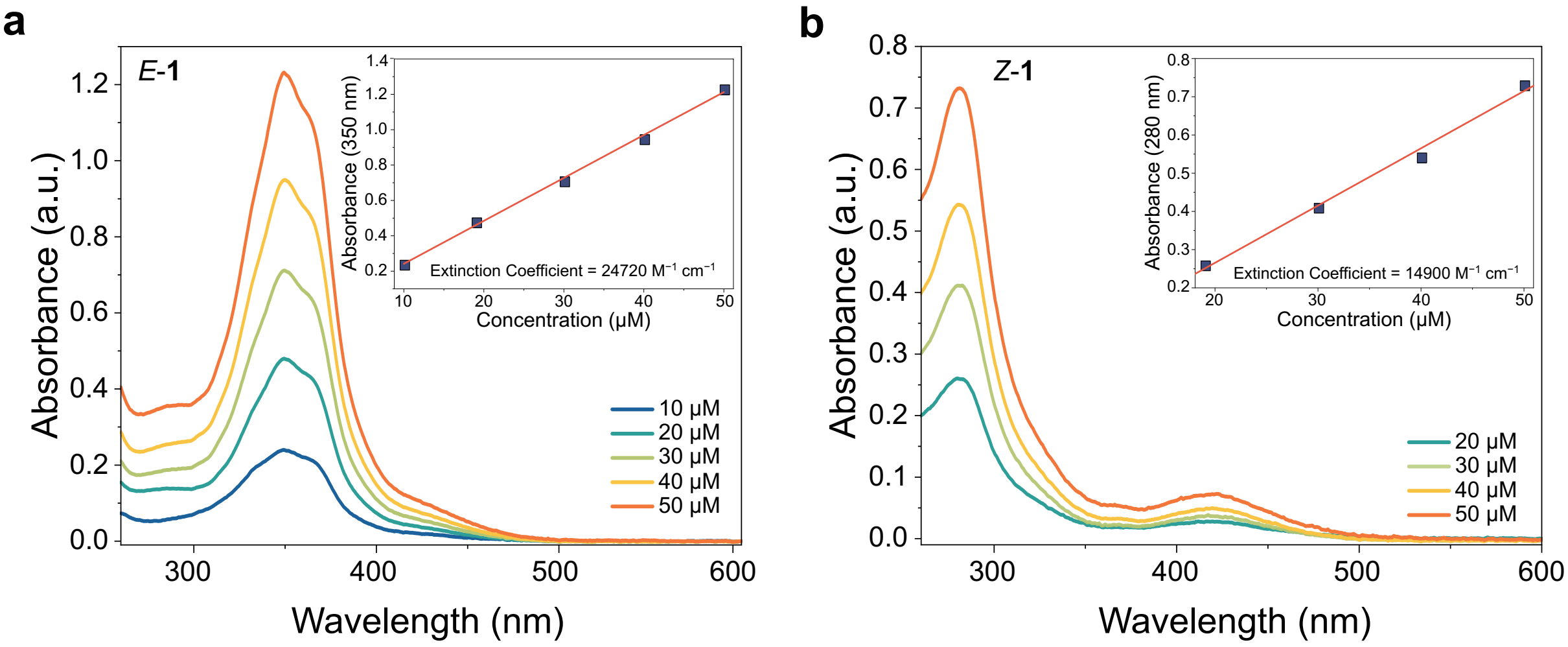

Figure S6. The UV/vis absorption spectra of the various concentrations of **a**, *E*-**1** and **b**, *Z*-**1** were measured at 20 °C (293 K) in DMSO. The calibration curves, shown in the top right corner of each figure, were plotted at the wavelength of λ_{max} to determine the slopes, which represent the extinction coefficients, as shown in Table S2.

Table S2. The wavelengths of maximum absorbance (λ_{max}) and the extinction coefficients at λ_{max} of *E*-**1** and *Z*-**1**.

	λ_{max} (nm)	ε (M^{-1} cm^{-1})
E-**1**	350	24720
Z-**1**	280	14900

3.2. Thermodynamic Properties

The thermal half-life ($t_{1/2}$) of *Z*-**1** was measured using UV/vis absorption spectroscopy. The sample of **1** was initially converted to *Z*-**1** by irradiation with 365 nm light. The rate constants (*k*) for the thermal process of *Z*-**1** to *E*-**1** were determined by fitting the absorbance changes over time (*t*) to an exponential (Equation 2, Figure S7).

$$[A]_t = [A]_0 e^{-kt} \tag{2}$$

The rate constant at room temperature, 20 °C (293 K), was estimated by extrapolating the Eyring plot, and the thermal half-life ($t_{1/2}$) was calculated from this. The expression for the $t_{1/2}$ is shown below.

$$t_{1/2} = \frac{\ln 2}{k} \tag{3}$$

The Eyring equation shown below:[6]

$$\ln\left(\frac{k}{T}\right) = -\frac{\Delta H^{\ddagger}}{R} \cdot \frac{1}{T} + \frac{\Delta S^{\ddagger}}{R} + \ln\left(\frac{k_B}{h}\right) \tag{4}$$

Gibbs energy of activation can be calculated using the Gibbs function:

$$\Delta G^{\ddagger} = \Delta H^{\ddagger} - T\Delta S^{\ddagger} \tag{5}$$

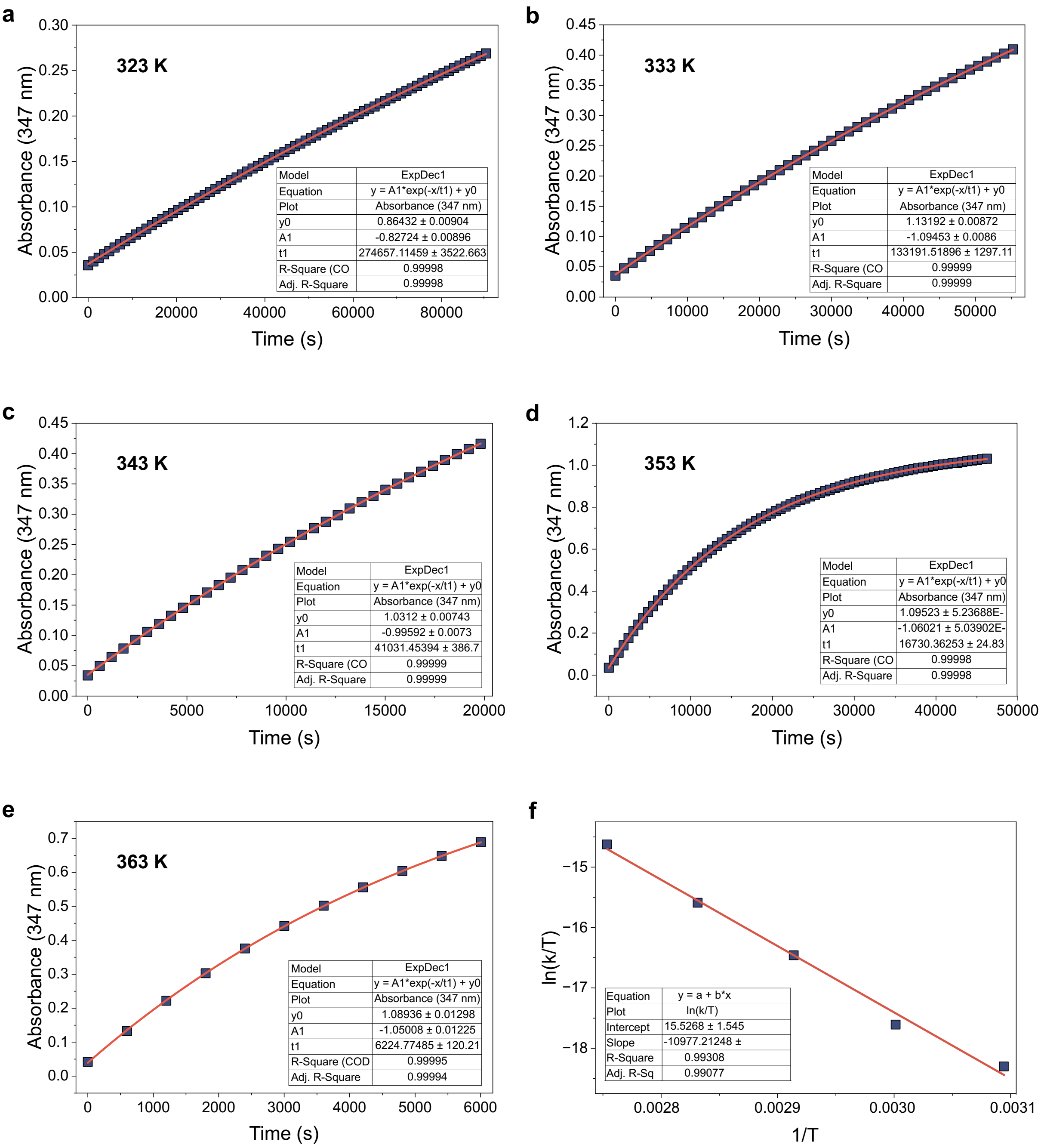

Figure S7. The change in absorbance of **1** at the λ_{max} over time at: **a**, 50 °C (323 K); **b**, 60 °C (333 K); **c**, 70 °C (343 K); **d**, 80 °C (353 K); **e**, 90 °C (363 K). The initial absorbance value corresponds to the absorbance of pure *Z*-**1** with the increase in absorbance relating to the thermal isomerization of *Z*-**1** to *E*-**1**. All samples were irradiated under 365 nm LED for 5 minutes in DMSO before the measurements started to ensure the 365 nm PSS was obtained. The exponential fittings were applied to determine the rate constants and $t_{1/2}$. **f**, The Eyring plot of **1** was generated using the rate constants calculated at different temperatures. The data exhibited linearity with R^2 values exceeding 0.99, and the fitted parameter was used to estimate the enthalpy of activation ($\Delta H^{\ddagger}$ = 91.3 kJ mol^{-1}) and an entropy of activation ($\Delta S^{\ddagger}$ = –68.5 J mol^{-1} K^{-1}). The thermal *Z*-**1** to *E*-**1** isomerism exhibits a Gibbs energy of

activation ($\Delta G^{\ddagger}$) at 20 °C (293 K) of 111.3 kJ mol^{-1}. At 20 °C (293 K), the $t_{1/2}$ is estimated to be 90.5 days.

3.3. Photostationary State (PSS)

To determine the composition of the *E*- and *Z*-isomers at different PSSs, a cuvette, with four transparent windows, containing a 42 µM sample of **1** was placed in the CCD photoswitching setup shown in Figure S1 and irradiated with LEDs ranging from 340 nm to 530 nm (Table S1).

The spectrum of the pure *E*-**1** was obtained after heating an *E*-rich sample at 80 °C (353 K) in the CCD setup (Figure S1) for two hours to ensure that any trace amounts of *Z*-**1** had thermally isomerized to the *E*-**1.** UV/vis spectra of the 340 nm to 540 nm PSSs were obtained by irradiating the sample with LEDs in the CCD setup (Figure S1) until no further spectral changes were observed. The samples were then swiftly moved to the Cary 3500 spectrophotometer to record a higher-resolution spectrum.

^{1}H NMR spectroscopy revealed that irradiation with 365 nm LED resulted in quantitative conversion of *E*-**1** to *Z*-**1** (Figure S8). Hence, the fractions of each isomer at the different PSSs were calculated from a linear extrapolation between the spectra of *Z*-**1** and *E*-**1** (Figure S9). The calculated %*E*-**1** and %*Z*-**1** at PSS are listed in Table S3.

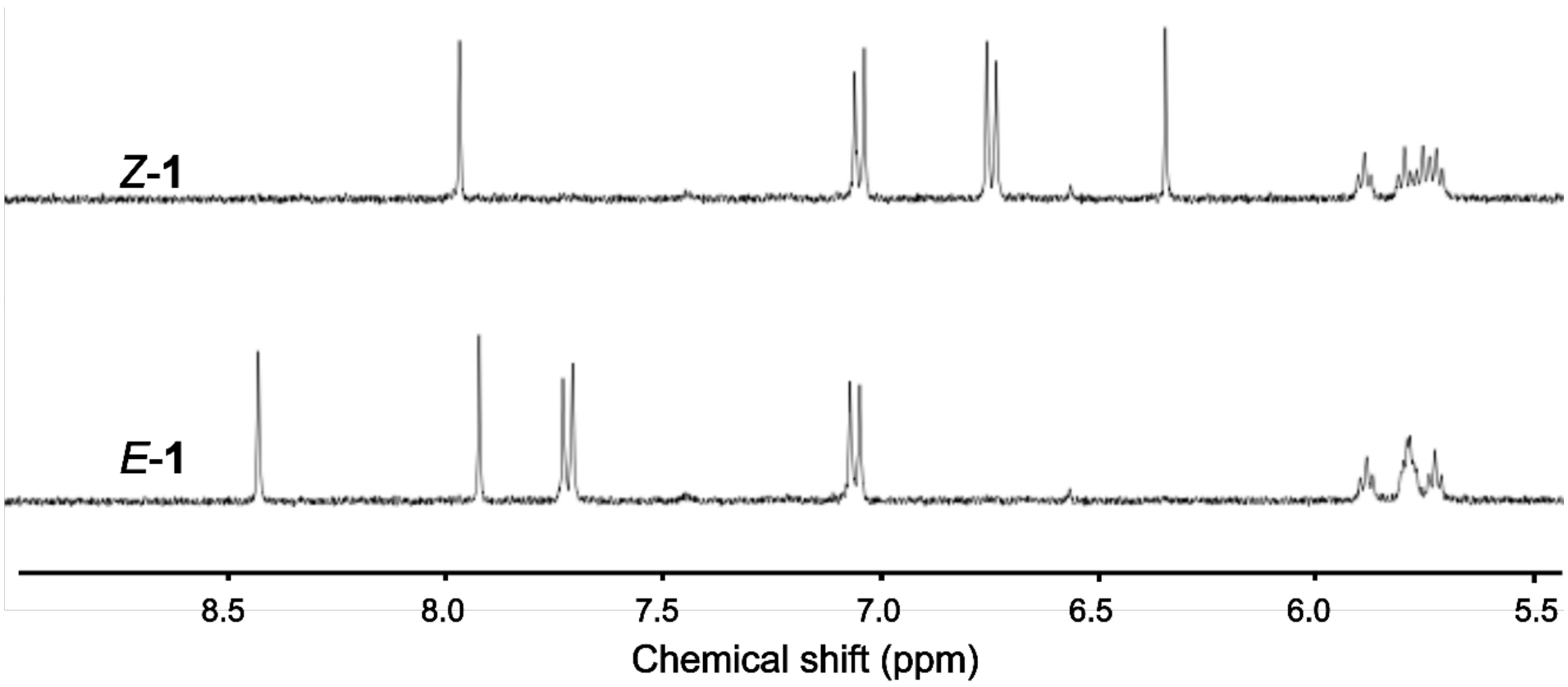

Figure S8. ^{1}H NMR spectra (400 MHz, 298 K, DMSO-d_6) of *E*-**1** before irradiation (bottom) and after irradiation at 365 nm LED (top). The sample was irradiated with 365 nm in the NMR tubes until complete switching is observed from the ^{1}H NMR spectra.

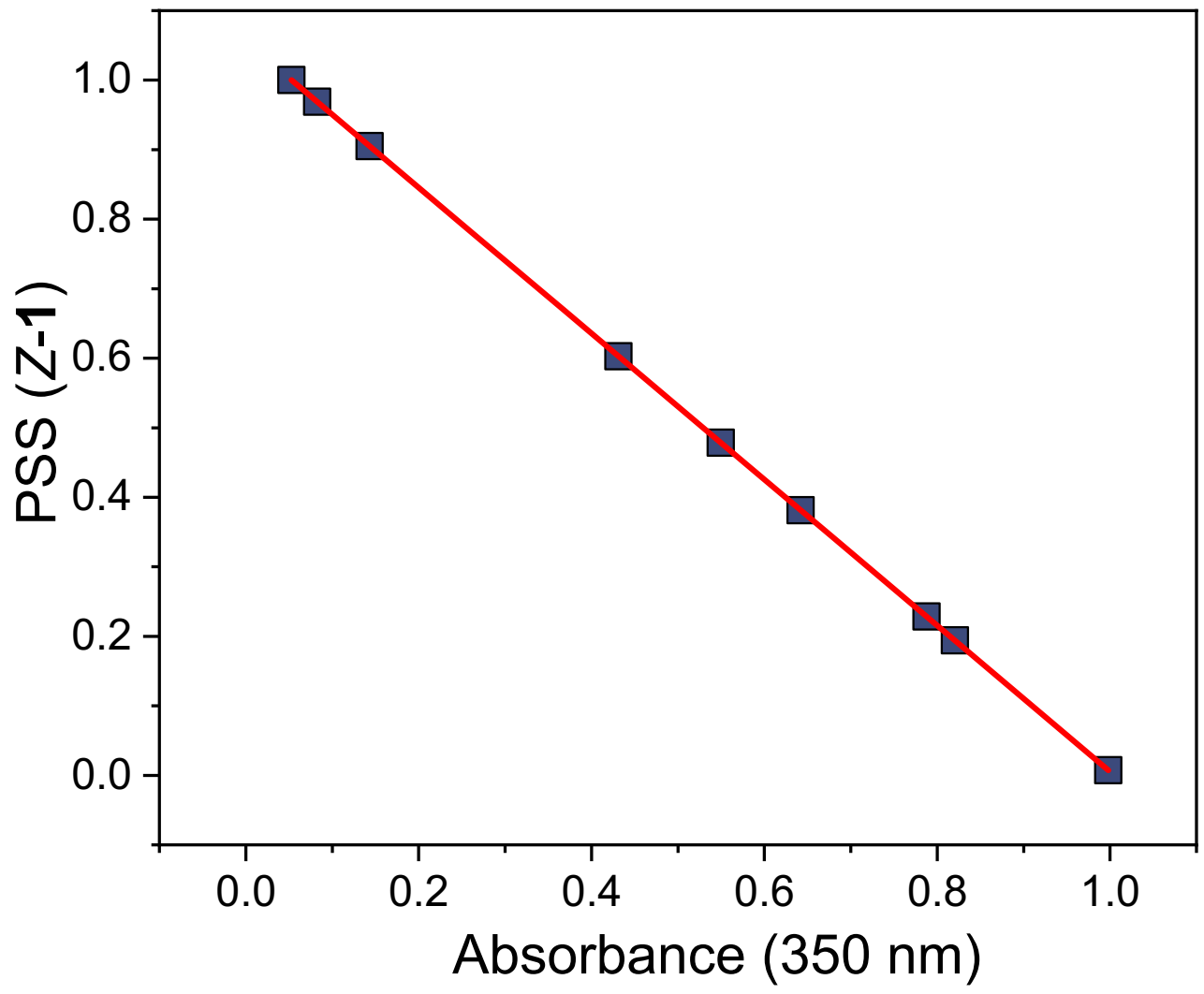

Figure S9. Fraction of *Z*-**1** at different PSSs, as calculated from the absorption of 42 μM of **1** in DMSO at 350 nm, measured at 20 °C (293 K).

Table S3. Fraction of *E*-**1** and *Z*-**1** at different PSS between 530 and 340 nm.

**Wavelength (nm)**	**PSS (% *E*-1)**	**PSS (% *Z*-1)**
530	99	1
505	81	19
490	77	23
470	62	38
430	52	48
405	40	60
385	10	90
365	0	100
340	4	96

The PSS compositions measured in DMSO were used as a guide for the light-responsive supramolecular polymerisation studies. To evaluate the validity of this estimate, we further measured the PSSs of a sample in H_2O/MeCN (4:1, *v/v*). To avoid contributions from the aggregated state appearing in the UV/vis spectra, we used a

2.5 µM solution of monomer **1**, along with a 10 cm path length cuvette. Importantly, this concentration was below that of the critical concentration.

Similar PSS compositions were obtained, compared to the DMSO studies, as shown in Figure S10, and Table S4.

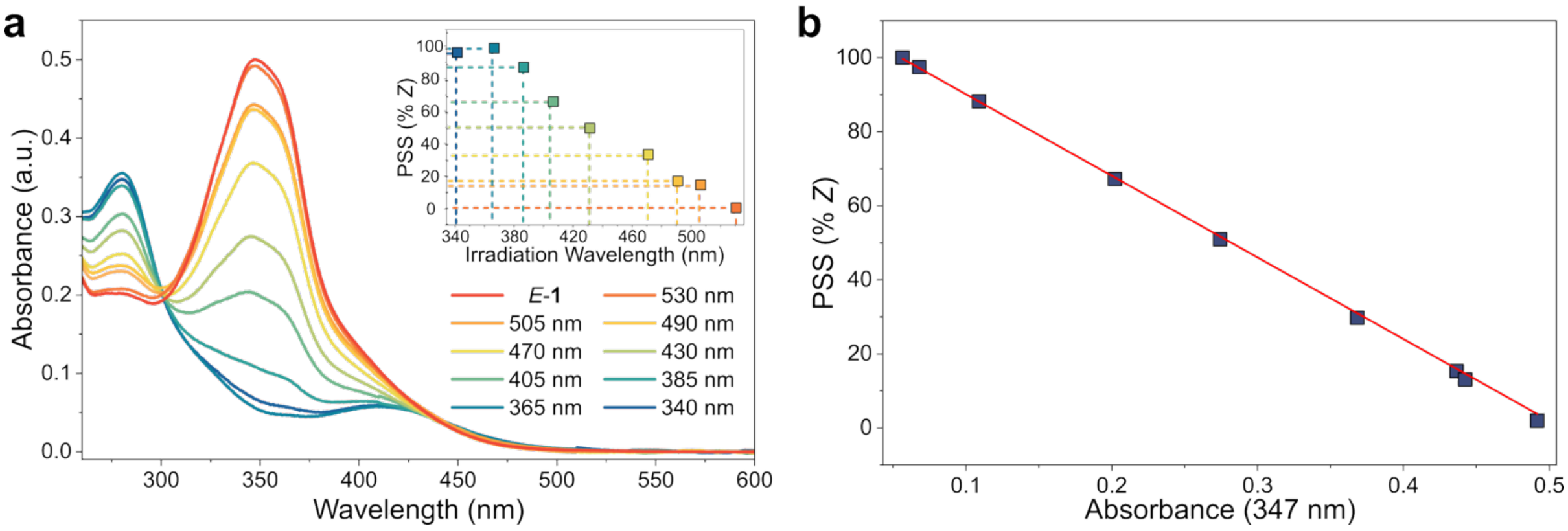

Figure S10. a, UV/vis absorption spectra of *E*-**1** in H_2O/MeCN (4:1, *v/v*) at 20 °C (293 K) after irradiation at different wavelengths. (**Inset**) Irradiation wavelengths from 340 to 530 nm provide broad coverage of isomer compositions. **b**, Fraction of *Z*-**1** at different PSSs, as calculated from the absorption of **1** in H_2O/MeCN (4:1, *v/v*) at 347 nm, measured at 20 °C (293 K). Note that these measurements were performed in a 10 cm pathlength cuvette, using a 2.5 µM solution of *E*-**1** to be below the critical concentration.

Table S4. Fraction of *E*-**1** and *Z*-**1** of a 2.5 µM sample of monomer **1** in H_2O/MeCN (4:1, *v/v*) at 20 °C (293 K) at different PSSs between 530 and 340 nm. This data was obtained from Figure S10.

Wavelength (nm)	PSS (% *E*-**1**)	PSS (% *Z*-**1**)
530	98	2
505	83	17
490	81	19
470	65	35
430	50	50
405	33	67
385	11	89
365	0	100
340	3	97

3.4. Fatigue Resistance

To test the fatigue resistance of the photoswitching unit, a 35 μM sample of **1** was switched back and forth 20 times between a *E*-rich and *Z*-rich PSS at 25 °C (298 K). This was achieved using 365 nm and 505 nm light. No observable decay in the UV/vis absorption spectra were observed across these 20 cycles, indicating a good resistance to fatigue (Figure S11).

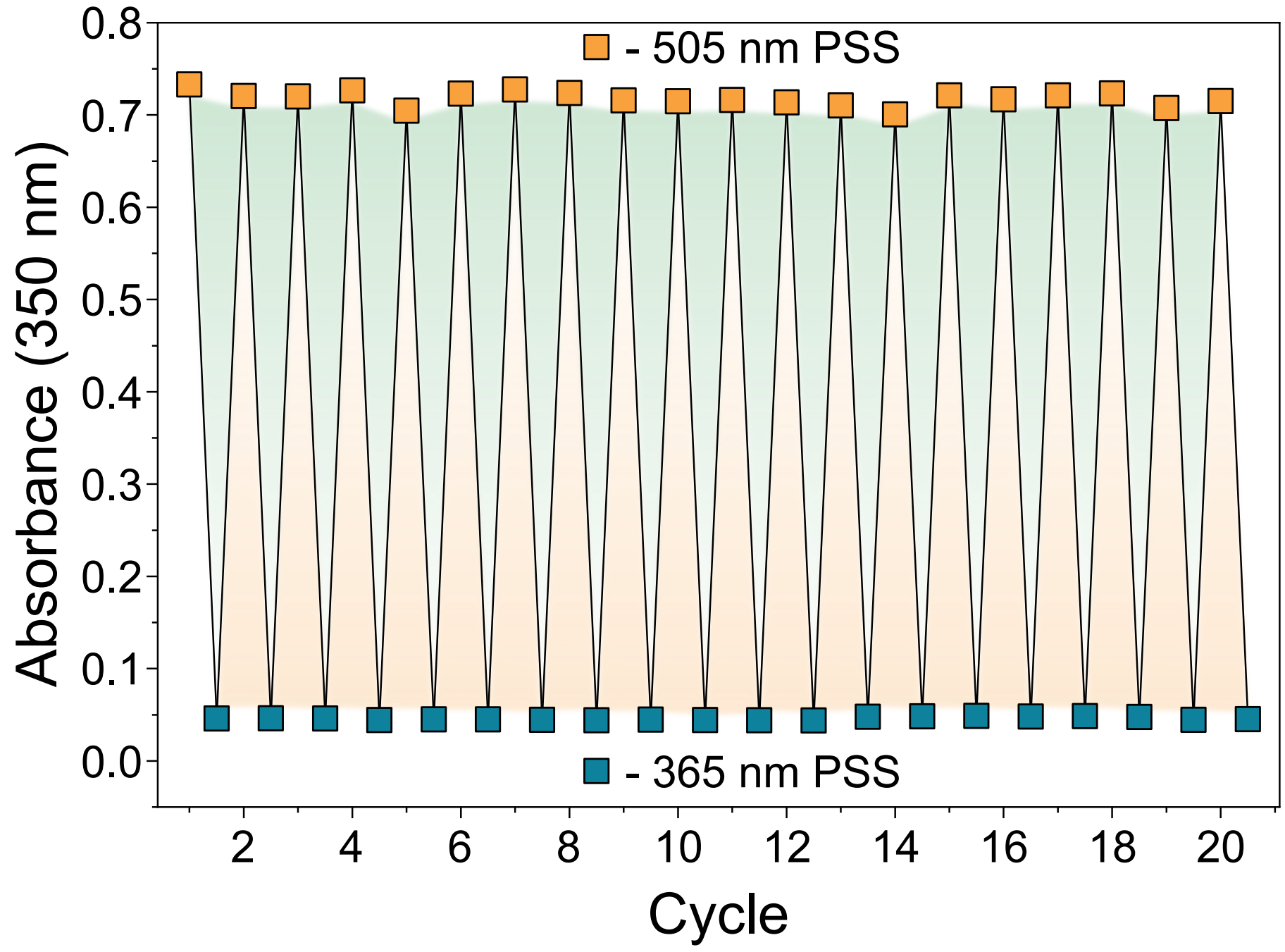

Figure S11. Fatigue resistance of **1** measured using UV/vis spectroscopy (35 μM in DMSO, 298 K) upon repeated photoisomerism with alternating 365 nm and 505 nm irradiation. No signs of degradation of the photoswitch were observed over 20 switching cycles.

3.5. Quantum Yields

The photon flux for the LED light sources were determined based on their measured power output (using a thermal power sensor and Thorlabs Optical Power Monitor software). Following the procedure reported by Crespi and co-workers,[1] the LED power was first measured without any quartz cuvette between the collimating lens and the thermopile. Then, the LED power was measured again with the cuvette containing the blank solvent. The power loss at the sample was approximated as half of the total power loss observed for the system.

The samples of *E*-**1** (35 µM, for *E*-**1** to *Z*-**1,** 365 nm) and *Z*-**1** (400 µM, for *Z*-**1** to *E*-**1,** 530 nm) were prepared in DMSO at 20 °C (293 K). The irradiation and measurements were performed in the CCD spectrometer setup (Figure S1), under the same irradiation conditions employed during the power determination process.

The reader is directed to the work of Crespi and co-workers for an in-depth discussion on the approach for calculating the quantum yields.[1] Briefly, the photoisomerization kinetics were analysed using a two-state model. The thermally stable and metastable isomers are denoted as A and B, respectively. For irradiation with a narrow-band LED (as shown in Table S1), the spectral width of the light source was neglected and the molar extinction coefficients and quantum yields were evaluated at the irradiation wavelength. The absorbed photons were partitioned between the two isomers according to their respective contributions to the total absorbance. The resulting rate equation is:

$$\frac{d[A]}{dt} = -\frac{\Phi_{A\to B} \cdot q_A}{N_A \cdot V} + \frac{\Phi_{B\to A} \cdot q_B}{N_A \cdot V} + k_{B\to A}[B] \qquad (6)$$

$$\frac{d[A]}{dt} = \frac{1}{V}\frac{q_0}{N_A}\frac{1-10^{-([A]\varepsilon_A+[B]\varepsilon_B)l}}{[A]\varepsilon_A+[B]\varepsilon_B}(\Phi_{B\to A}[B]\varepsilon_B - \Phi_{A\to B}[A]\varepsilon_A) + k_{B\to A}[B] \qquad (7)$$

Here, q_0 is the photon flux, V is the irradiated sample volume, N_A is Avogadro's number, $\Phi_{A\to B}$ and $\Phi_{B\to A}$ are the photoisomerization quantum yields, and $k_{B\to A}$ is the first-order rate constant for thermal relaxation of the metastable isomer. The total photoswitch concentration was assumed to remain constant, supported by their excellent resistance to fatigue upon photoswitching (Figure S11):

$$[A] + [B] = c_0 \tag{8}$$

The ordinary differential equation shown in equation (7) was solved numerically using the SciPy package to obtain the quantum yields of photoisomerism.[1]

Table S5. The summary of quantum yields of photoisomerization using 365 nm and 530 nm LEDs.

	Quantum yield of photoisomerization
E-**1** to *Z*-**1** (365 nm)	0.71
Z-**1** to *E*-**1** (530 nm)	0.43

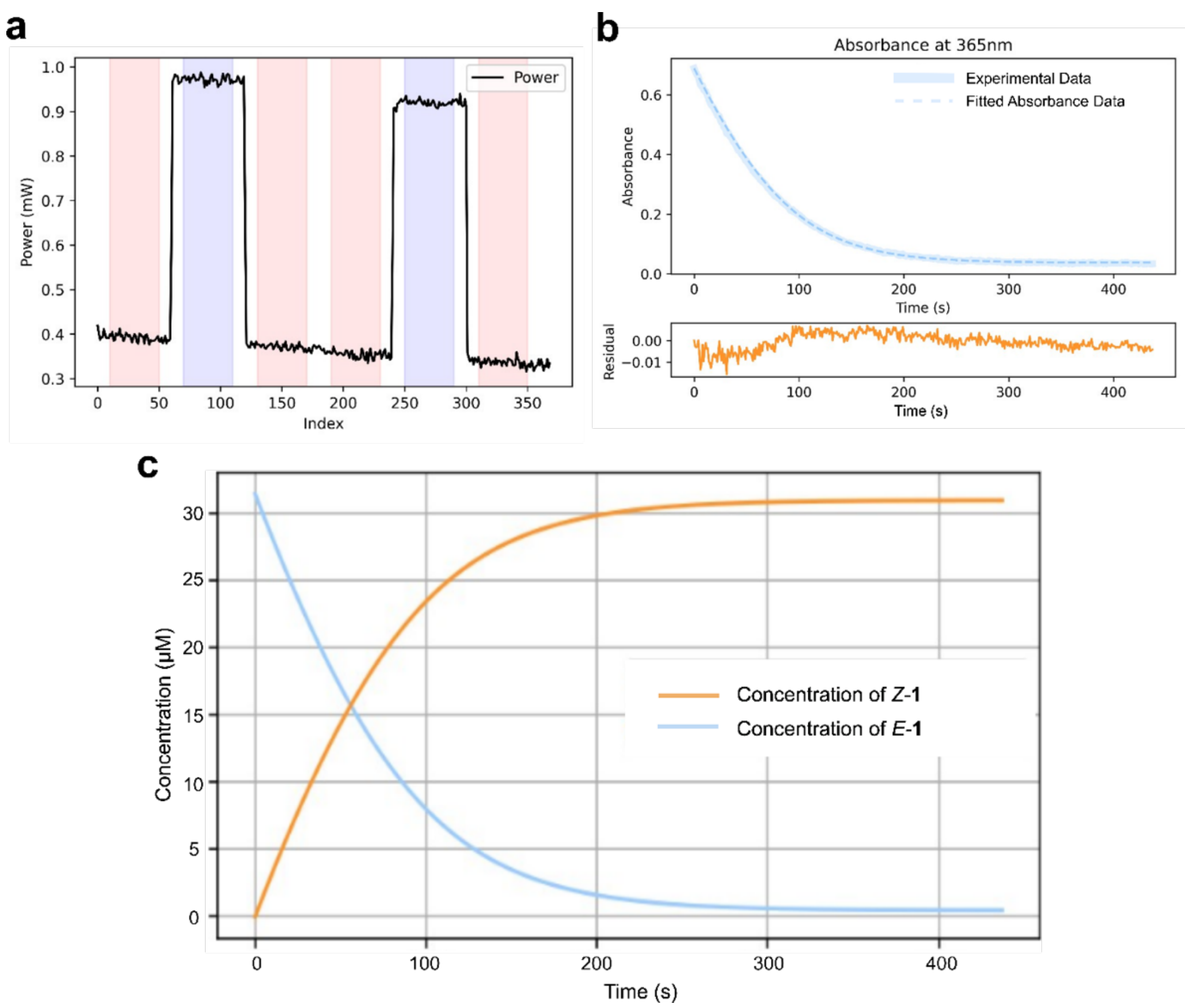

Figure S12. Photoswitching quantum yield of **1** (*E*-**1** to *Z*-**1**) under 365 nm (200 mA and 20 percent brightness). **a**, Power determination, showing the power measured without any elements in the optical path (left) and with the insulated jacket and cuvette (right). The blue shaded regions correspond to when the LED is switched on, and the red regions when it is switched off, enabling the background to be correctly measured. **b**, Absorption at 365 nm over time, with the fitted total absorbance curve indicated by a dashed line. **c**, Concentration of *E*-**1** and *Z*-**1** over time under 365 nm irradiation.

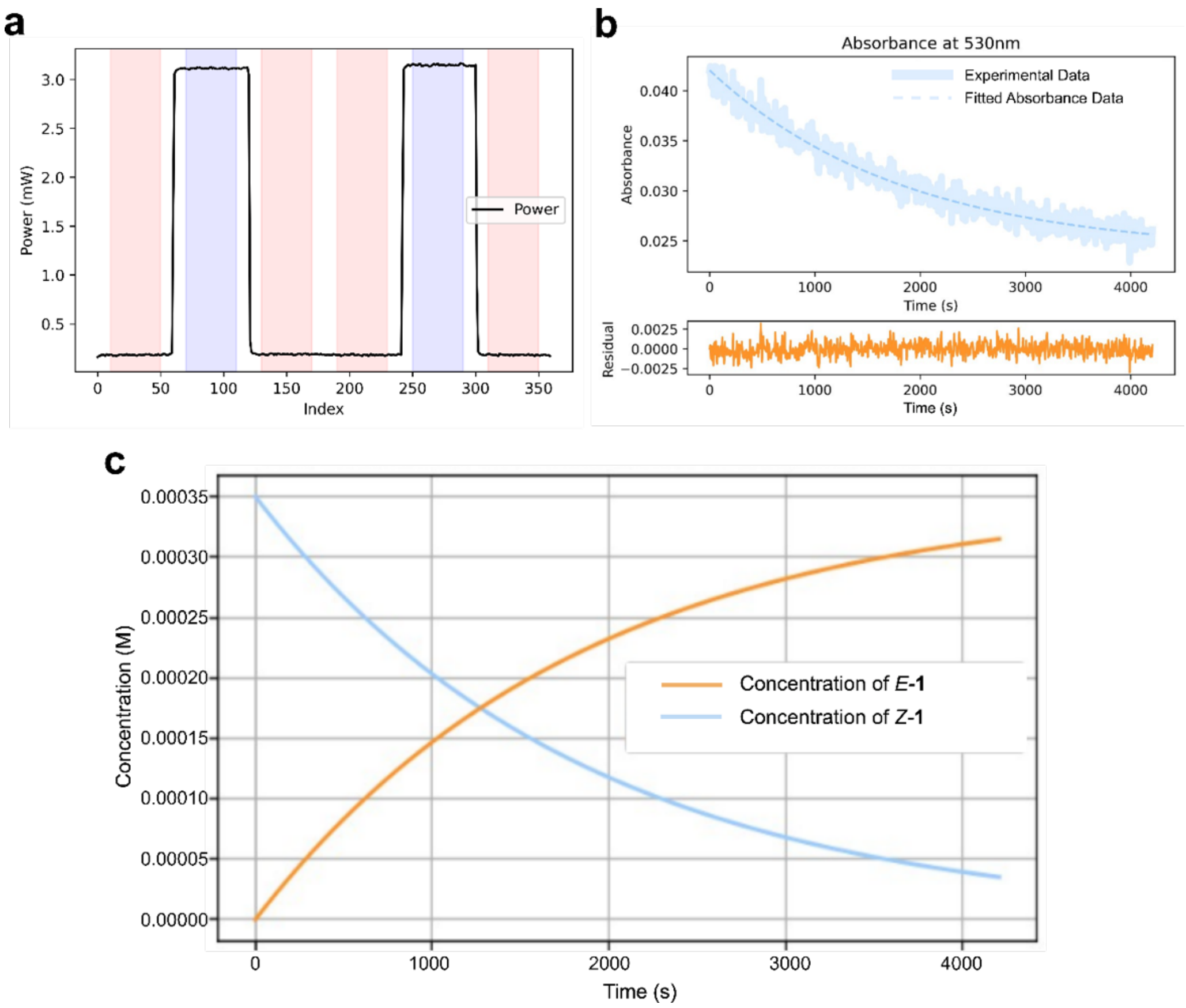

Figure S13. Photoswitching quantum yield of **1** (*Z*-**1** to *E*-**1**) under 530 nm irradiation (800 mA current and 90% brightness). **a**, Power determination, showing the power measured without any elements in the optical path (left) and with the insulated jacket and cuvette (right). The blue shaded regions correspond to when the LED is switched on, and the red regions when it is switched off, enabling the background to be correctly measured. **b**, Absorption at 530 nm over time, with the fitted total absorbance curve indicated by a dashed line. **c**, Concentration of *E*-**1** and *Z*-**1** over time under 530 nm irradiation.

4. Investigation of the Polymerization Process

4.1. Solvent Screening

Binary mixtures of water and organic solvents were prepared at a fixed concentration of *E*-**1** (35 μM). This was to ensure a UV/vis absorbance below a value of 1. The degree of aggregation (α) of *E*-**1** into **2** was modulated by varying the temperature: at low temperatures, the degree of aggregation, α, is high and at high temperatures it is low.

During this study, we sought to identify a solvent system that enabled monomer **1** to remain soluble in the non-assembled state, and that could support the assembly of this monomer into polymer **2** at lower temperatures. Importantly, supramolecular polymer **2** must also remain in solution and avoid precipitating out. A schematic of the challenges encountered during this screen is shown below in Figure S14.

Based on this approach different solvent mixtures (as shown in Table S6 and Figure S15) were explored.

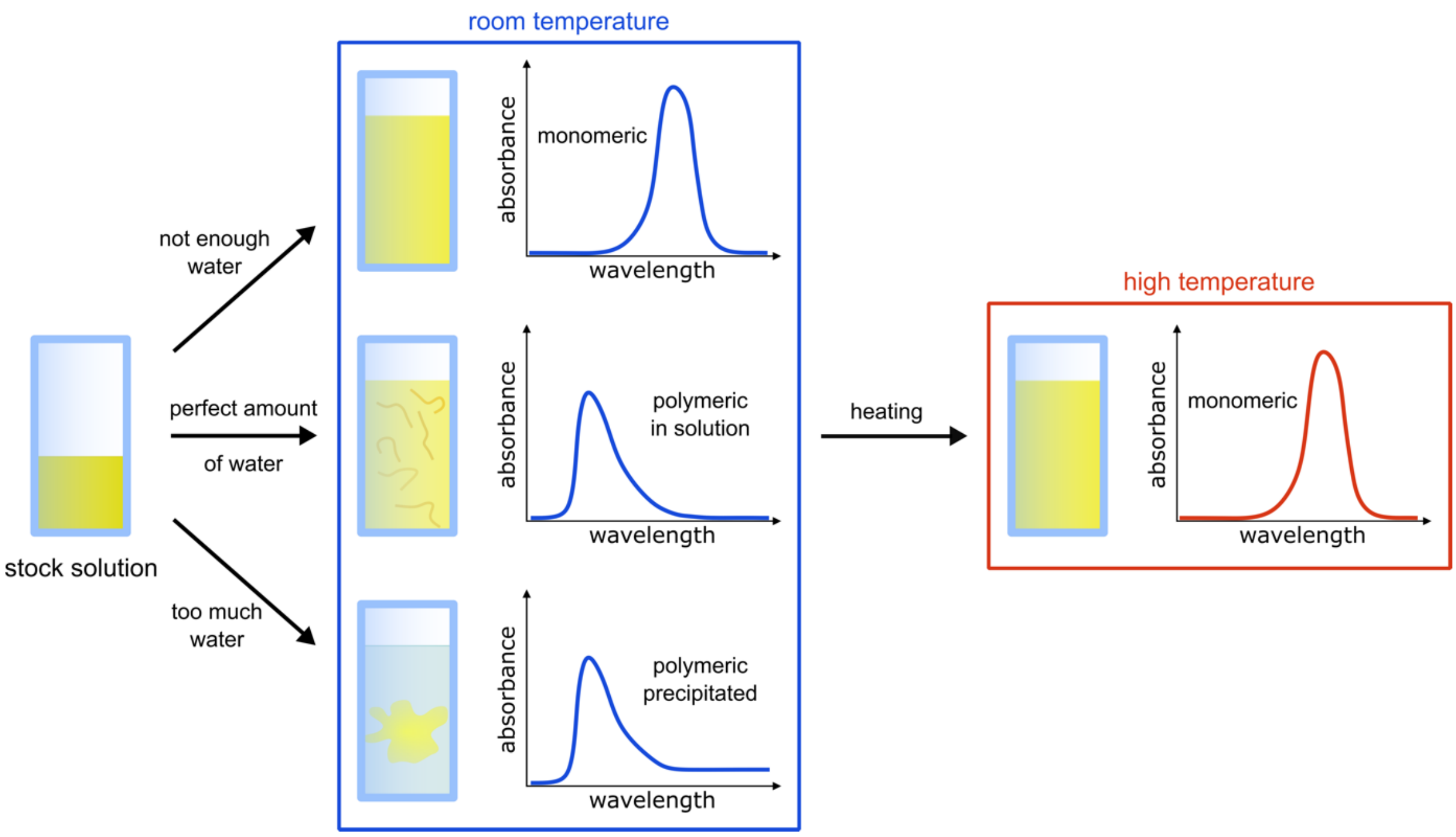

Figure S14. Schematic illustration of the solvent screening process. From each stock solution of **1** in organic solvent, 35 μM solutions containing different fractions of water were prepared. UV/vis absorbance spectroscopy was used to identify solutions with ideal properties, *i.e.*, solutions in which *E*-**1** polymerizes at 20 °C (293 K) without precipitating and disassembles into monomers upon heating. Too low a fraction of water resulted in a low degree of polymerization at 20 °C (293 K) whereas too low a fraction of water caused precipitation of the polymers.

Table S6. Solvent mixtures screened and the suitable solvent combination identified during the screening.

Solvent mixture	Suitable solvent mixture (H_2O/X)
H_2O/Ethanol	Not found
H_2O/THF	Not found
H_2O/DMF	60/40
H_2O/MeCN	80/20
H_2O/DMSO	40/60

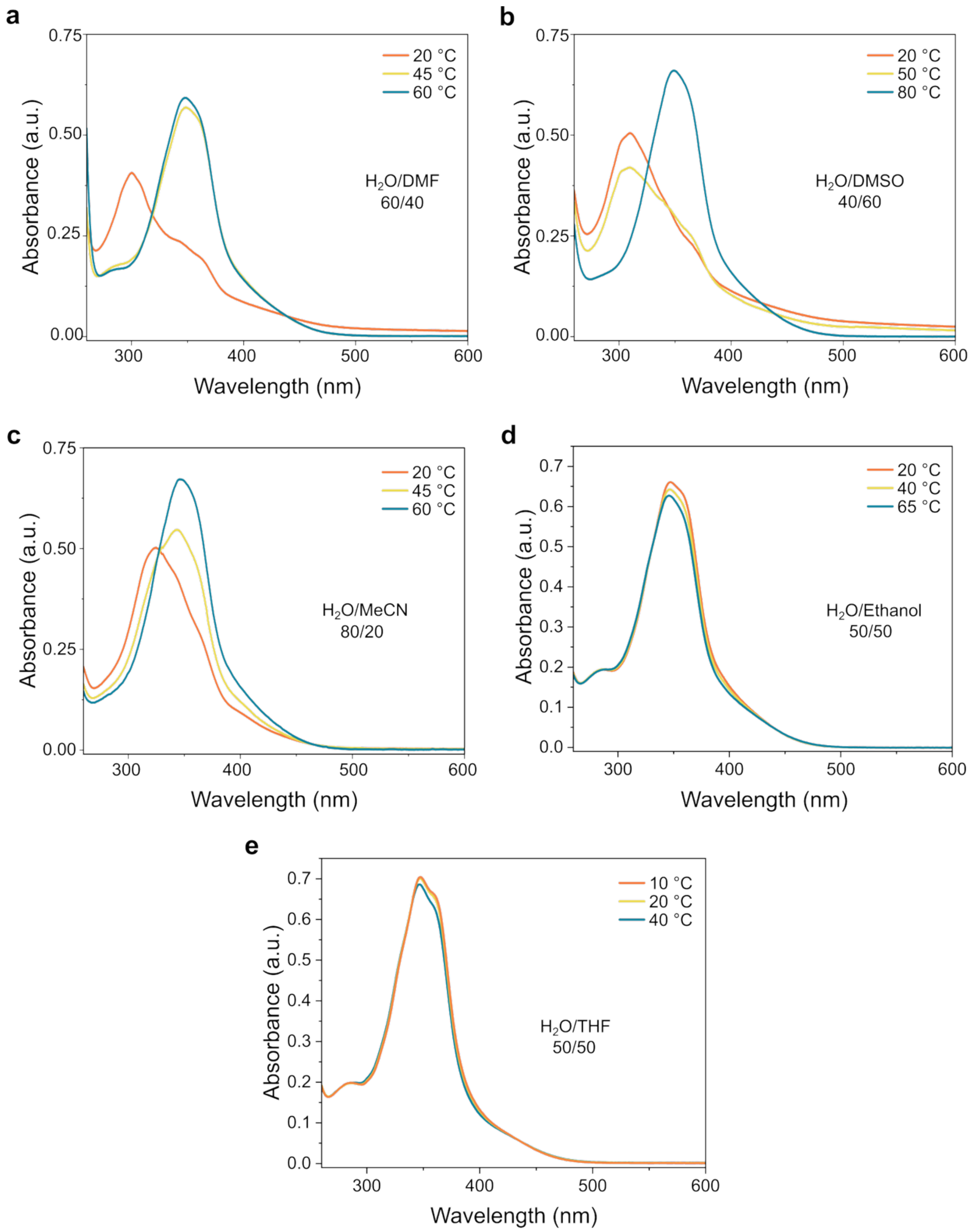

Figure S15. UV/vis absorption spectra of 35 μM solutions of *E*-**1** in different solvent mixtures at various temperatures. For **a-c**, lower temperatures, *i.e.*, 20 °C (293 K), exhibit an aggregate spectrum; while high temperatures, *i.e.*, 60 °C (333 K) or 80 °C (353 K), exhibit a more monomer-like spectrum. **a**, H_2O/DMF (60/40). **b**, H_2O/DMSO (40/60). **c**, H_2O/MeCN (80/20). In contrast, **d**, H_2O/EtOH (50/50) and **e**, H_2O/THF (50/50) reveal no significant aggregation across the temperature range for the compositions shown.

The solvent screening revealed that *E*-**1** does not aggregate in the pure organic solvents. Addition of water to the monomer solution in organic media induced aggregation leading to the formation of H-type aggregates. Among the solvents

examined, DMF, DMSO and MeCN afforded the behaviour we sought, with a H_2O/MeCN (4:1, *v/v*) system being selected for further studies into the aggregation behaviour. This choice of solvent system was made for its lower UV cut-off, resulting in reliable UV/vis measurements below 300 nm, and that the aggregates formed in this mixture exhibited substantially less light scattering (non-zero baseline at longer wavelengths in the UV/vis spectra) than those formed in H_2O/DMF and H_2O/DMSO, at the concentrations used. No evidence of significant aggregation was observed in the H_2O/EtOH or H_2O/THF mixtures at low temperatures. We speculate that EtOH and THF more effectively solvate both parts of the amphiphilic side chain, while also providing hydrogen-bonding interactions with the urea moieties, disfavouring the polymerisation.

To confirm that *Z*-**1** does not polymerise into polymer **2** in a H_2O/MeCN (4:1, *v/v*), the sample (35 μM, 293 K) was irradiated with 365 nm to reach the PSS, and a UV/vis spectrum was recorded on Agilent Technologies Cary 3500 Series UV/vis spectrophotometer. The resulting spectrum resembled that of *Z*-**1** in DMSO. To further investigate this, the sample was stirred at room temperature in the dark for 1.5 hours and monitored by time-dependent UV/vis absorption spectroscopy. No evidence for the formation of polymer **2** or extended fibrous aggregates by *Z*-**1** was observed under these conditions.

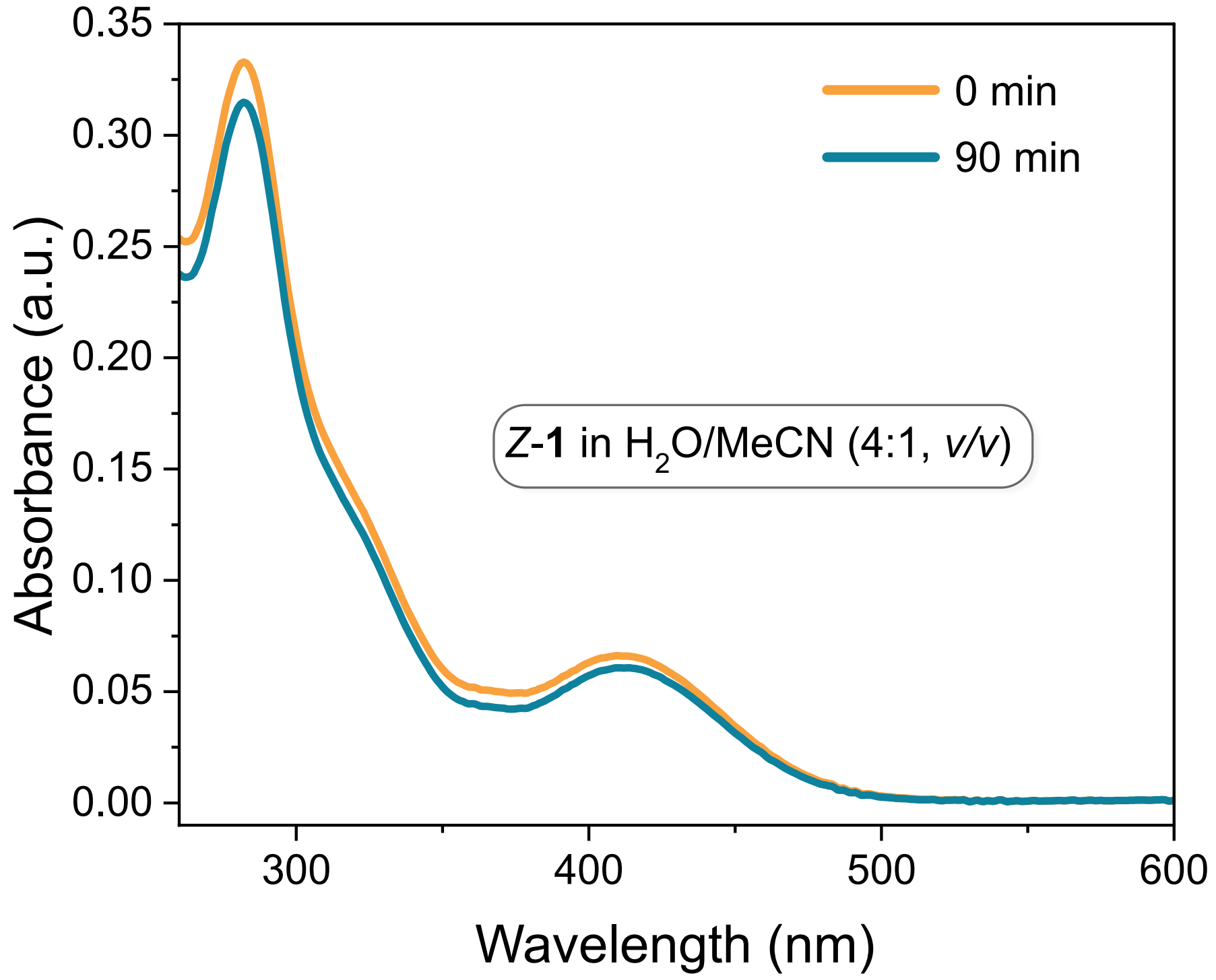

Figure S16. UV/vis absorbance spectra of a 35 μM sample of *Z*-**1** in H_2O/MeCN (4:1, *v/v*) at 20 °C (293 K) immediately after preparation of the sample and after 1.5 hours.

4.2. DLS Studies

To further confirm the absence of aggregates in samples of *Z*-**1**, as well as the formation supramolecular fibres for *E*-**1**, DLS experiments were conducted. Initially, samples of *E*-**1** (35 μM) were prepared in a mixture of H_2O/MeCN (4:1, *v/v*) and filtered through a 0.2 μm PTFE membrane filter to remove dust and other particulates in the solution. However, this resulted in significant sample loss, which we attributed to large polymeric fibres being captured in the filter. Instead, we changed the procedure so that samples were initially converted to the *Z*-isomer and then filtered. As the *Z*-isomer didn't form large aggregates, the material could readily pass through the filter.

As shown in Figure S17, DLS measurement of the *Z*-**1** samples revealed the particle diameter is in the range of 8 - 15 nm. Subsequent irradiation of these samples with 530 nm light induced *Z*-to-*E* isomerization, resulting in the formation of large particles (650 - 1500 nm). A 6-fold increase in the scattering count rate also confirmed the existence of large particles in the system (Table S7).

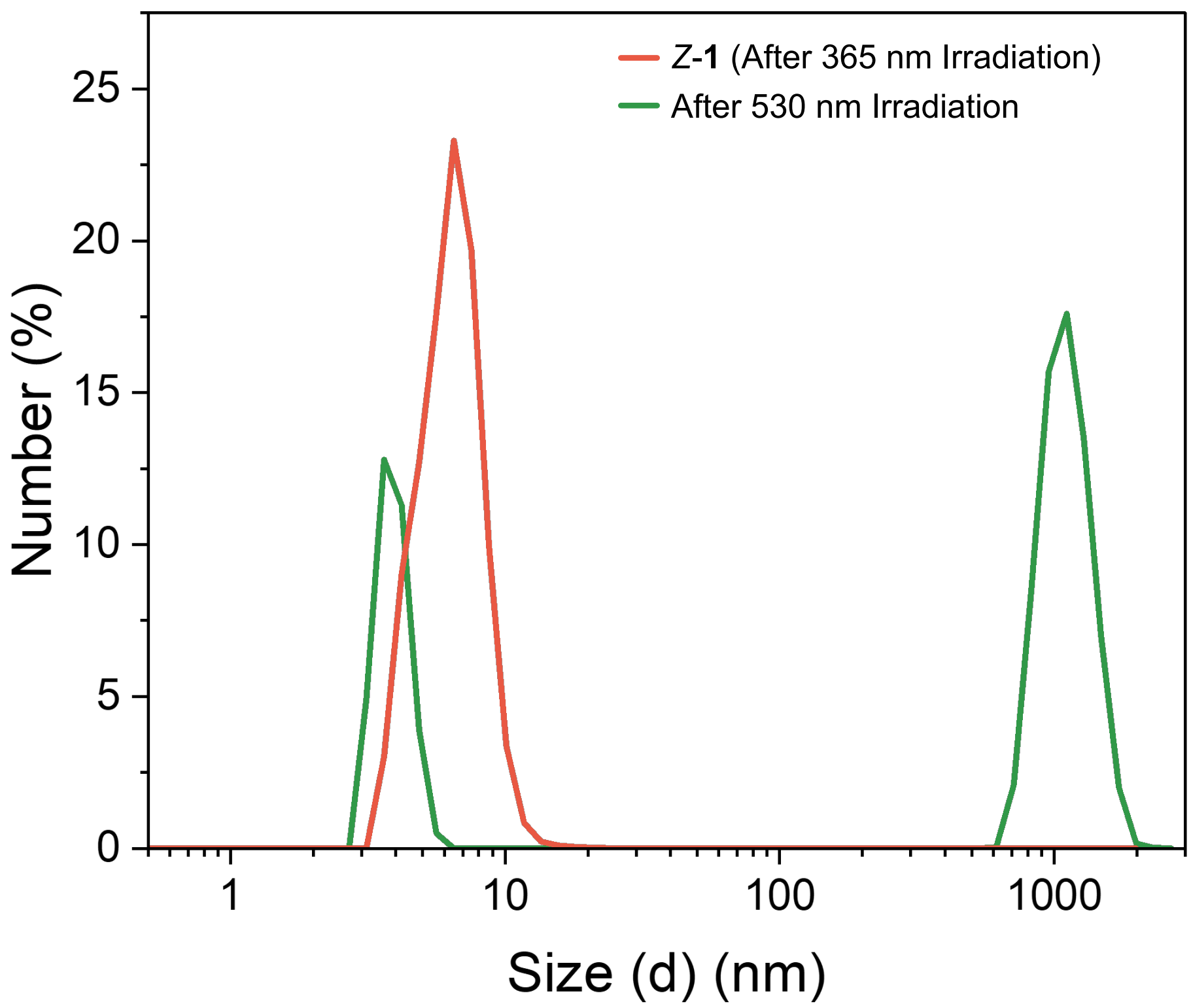

Figure S17. DLS data of 80 μM sample of *Z*-**1** in H_2O/MeCN (4:1, *v/v*) (red line) and after irradiation with 530 nm for 12 hours to convert it to *E*-**1**.

Table S7. Change in the count rate upon irradiation.

	Count rate (kcps)
Z-**1**	197.5
After Irradiation With 530 nm	1308.8

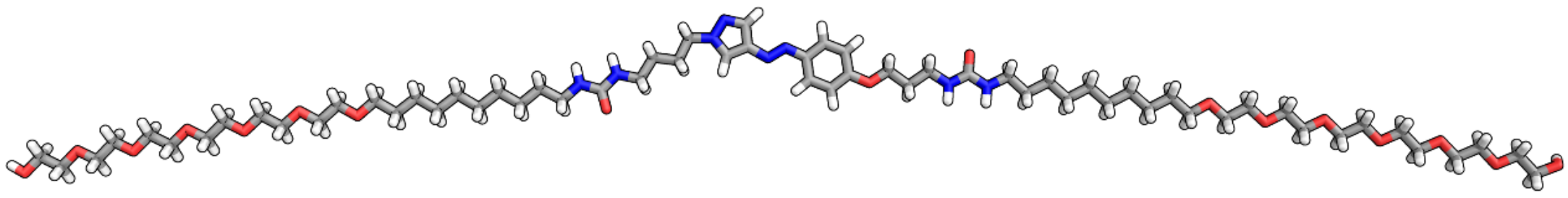

Figure S18. Optimised molecular geometry of *E*-**1** generated using molecular mechanics modelling (MMFF94s).

4.3. Investigation of the Polymerization Mechanism

Supramolecular polymers consist of repeating monomer units held together by non-covalent interactions, and such monomeric species can be assembled via different mechanisms. Two common mechanisms are the isodesmic and the cooperative (nucleation-elongation) mechanisms.[7,8] In the isodesmic (equal-K) model, the binding constant is identical for every polymerization step, analogous to step-growth polymerization. While cooperative model has two regimes (nucleation and elongation) with distinct binding constants, analogous to chain-growth polymerization.

To determine the polymerization mechanism of *E*-**1** into **2**, temperature-dependent and concentration-dependent UV/vis absorption spectroscopic studies were performed. The resulting spectral changes at the λ_{max} of *E*-**1** were expressed in terms degree of aggregation (α), calculated at each temperature/concentration using equation (9). The resulting α values were then plotted as a function of temperature/concentration and fitted using isodesmic and nucleation-elongation models to determine polymerization mechanism of *E*-**1**.[7–9]

$$\alpha_{\mathrm{Agg}}(T\ or\ c) = \frac{\varepsilon_{Mon} - \varepsilon_{(c\ or\ T)}}{\varepsilon_{Mon} - \varepsilon_{Agg}} \tag{9}$$

where $\varepsilon(c)$ or $\varepsilon(T)$ is the extinction coefficient at a given concentration or temperature, ε_{mon} is the extinction coefficient of the monomer, and ε_{agg} is that of the aggregate.

The Temperature-dependent (Section 4.3.1) and concentration-dependent (Section 4.3.2) behaviour of *E*-**1** is consistent with a nucleation-elongation polymerization mechanism.

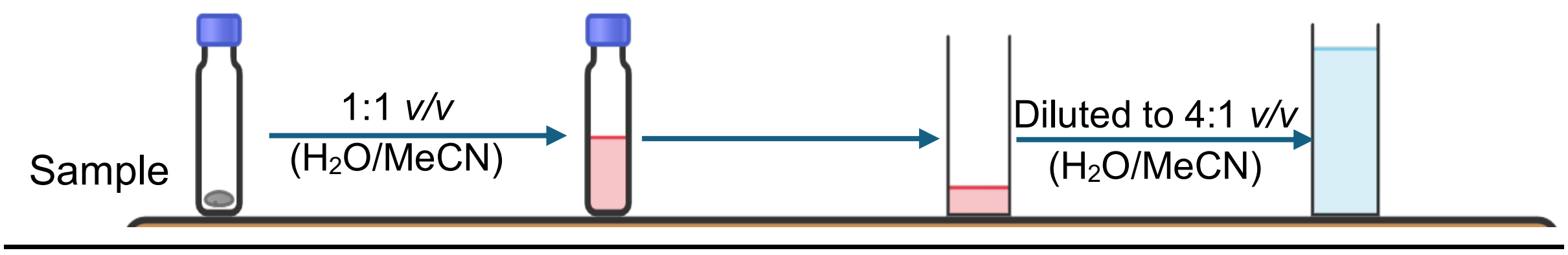

Figure S19. Sample preparation procedure for self-assembly studies.

4.3.1. Temperature-dependent UV/vis Measurements of *E*-**1**

Samples of *E*-**1** were prepared from a 300 µM stock solution (2 mL) in H_2O/MeCN (1:1, *v/v*). Samples at 25, 35, and 45 µM were prepared by diluting the stock solution into H_2O/MeCN (4:1, *v/v*), as shown in Figure S19.

For temperature-dependent UV/vis studies, each sample was first equilibrated at 70 °C (343 K) The sample was then cooled from 70 (343 K) to 4 °C (277 K) at a rate of 0.4 °C min^{-1}, with spectra recorded every 240 s under constant stirring. Only data recorded between 60 °C and 10 °C (333 to 283 K) were used for fitting.

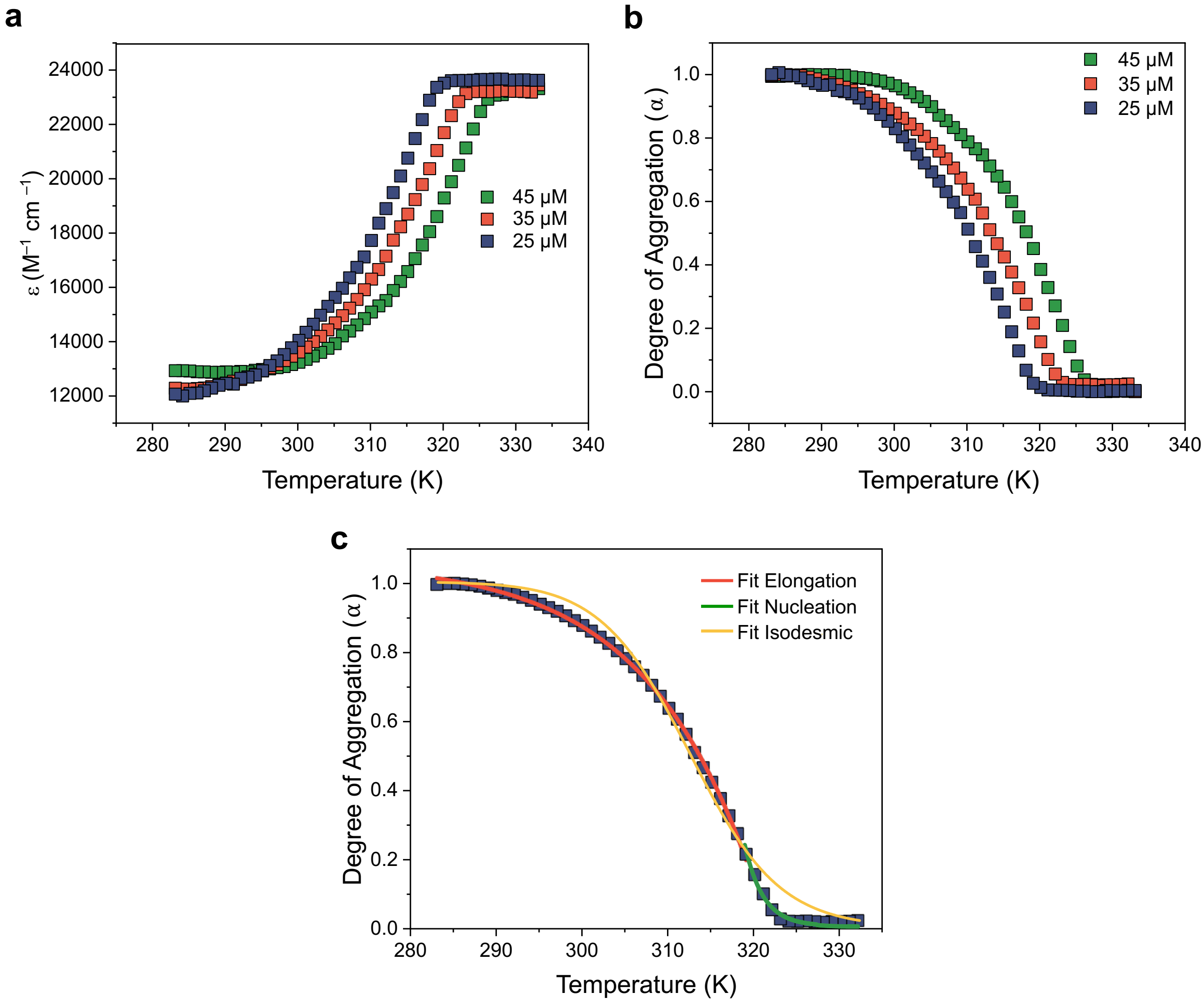

Figure S20. Temperature-dependent UV/vis data of *E*-**1** in H_2O/MeCN (4:1, *v/v*) upon cooling from 60 (333 K) to 10 °C (283 K) with a rate of 0.4 °C min^{-1} for different concentrations (25, 35, 45 μM). **a**, Change in the extinction coefficient of each solution calculated from the absorbance at 347 nm using Beer-Lambert law at different temperature. **b**, Plots of the degree of aggregation (α), calculated from the apparent extinction coefficients (at 347 nm), against the temperature for the disassembly processes for each concentration (25, 35, 45 μM). **c**, Plots of the degree of aggregation (α), calculated from the apparent extinction coefficients at 347 nm of a 35 μM sample, the plot is fitted with nucleation (green curve)-elongation (red curve) model and isodesmic model (yellow curve) for comparison showed a better overlay with the nucleation-elongation model (R^2 = 0.998).[7,8] elongation temperature, 48 °C (321 K) and molar enthalpy releases ΔH_E = –68.49 kJ mol^{-1} (upon elongation for temperature-dependent studies).

4.3.2. Concentration-dependent UV/vis Measurements of *E*-1

Note that one measurement at 7.5 μM was repeated after exhibiting anomalous behaviour. Two independent repeat measurements (at 7.2 μM and 8 μM) were consistent with the surrounding trend, and the original value was attributed to a sample preparation error and excluded from the final analysis.

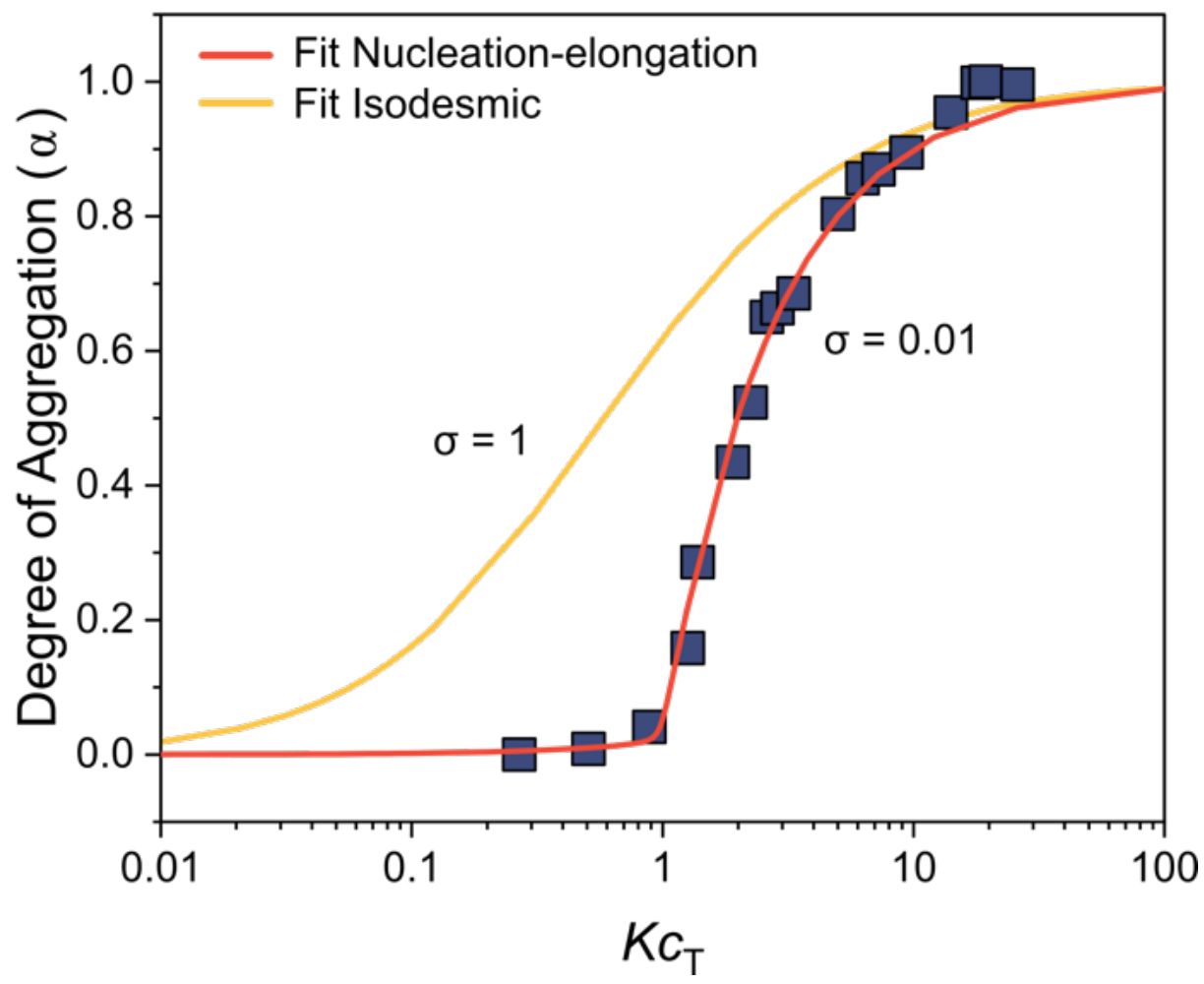

Figure S21. Plot of the degree of aggregation (α) at 347 nm against Kc_T and fit with the cooperative K_2/K model[9] with different σ values (red curve, σ = 0.01) and (yellow curve, σ = 1, isodesmic) and an elongation constant K = 3.8×10^5 M^{-1}. The sample was prepared in H_2O/MeCN (4:1, *v/v*) and measured at 20 °C (293 K)

Furthermore, to ensure that the sample was at equilibrium following dilution, a UV/vis spectra was recorded immediately after, and after 60 minutes (Figure S22).

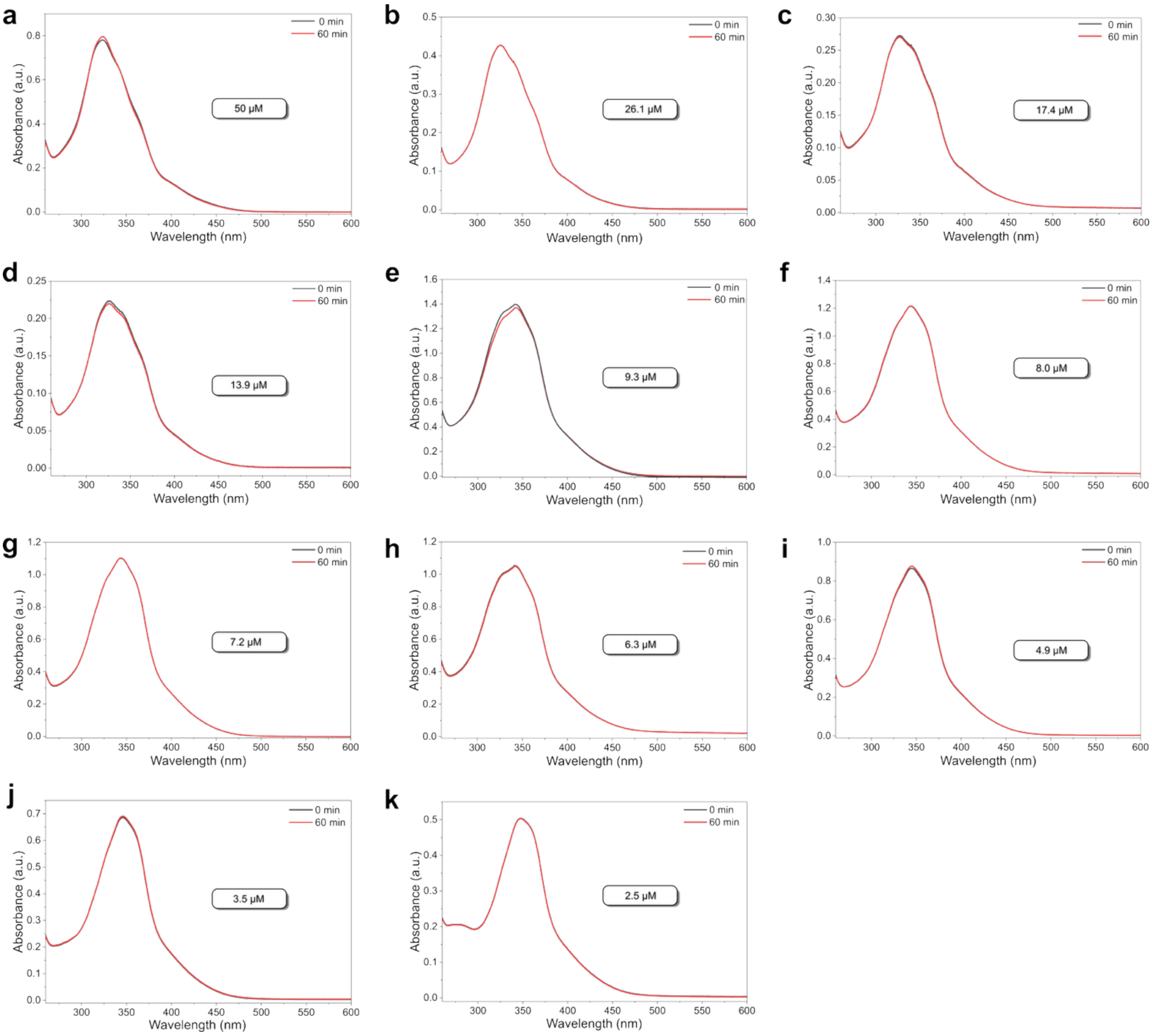

Figure S22. UV/vis absorption spectra of *E*-**1** prepared at different concentrations in H_2O/MeCN (4:1, *v/v*) at 20 °C (293 K). The black traces correspond to the sample directly after being diluted to the desired concentration, while the red traces correspond to the sample after 60 minutes of equilibration. A 10 mm pathlength cuvette was used for **a**-**d**, and a 100 mm pathlength cuvette was used for **e**-**k**.

5. Condensed Phase Studies

5.1. Condensed Phase Photoswitching

UV/vis measurements were performed on drop-cast films of **2** deposited on quartz substrates using a JASCO V-770 spectrophotometer equipped with an integrating sphere. Upon irradiation with 365 nm light, the UV/vis spectrum changed in a way that was consistent with the *E*-to-*Z* isomerism observed in solution (Figure S23). This indicates that switching can also occur in the condensed phase. Subsequent irradiation with 530 nm light for 6 h restored the original spectrum, demonstrating that the photoisomerisation is reversible in the thin film. The predicted *Z*-isomer spectrum was estimated using the Fischer method.[10]

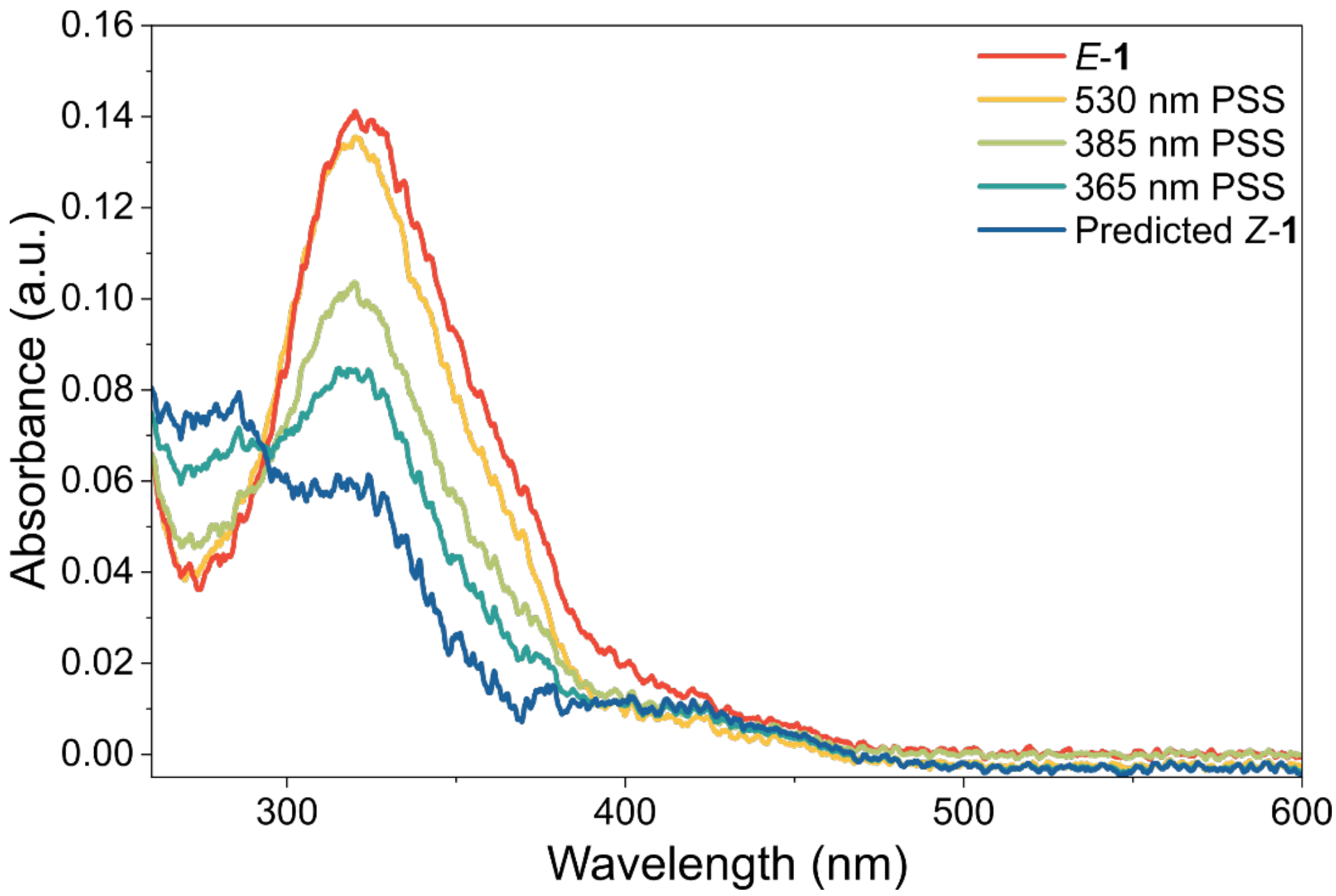

Figure S23. UV/vis spectra of drop-cast films of **2** before irradiation, and after irradiation with 365 nm, 385 nm and 530 nm light. The predicted spectrum of the *Z*-isomer, obtained using the Fischer method, see reference 10, is also shown. The spectral changes demonstrate photoisomerization in the condensed phase.

5.2. AFM Measurements

5.2.1. Morphology of *E*-1 Assemblies

AFM studies were performed using a 35 μM solution of **1** in H_2O/MeCN (4:1, *v/v*). The solution was heated to 70 °C (343 K), then cooled to room temperature, 20 °C (293 K), and drop-casted on a silicon wafer. The measurements revealed rope-like fibres with a diameter of 4.9 ± 0.3 nm and lengths of up to a few microns. The pitch of the helix varied between 25 and 30 nm. The fibres exhibited mixtures of left (*M*)- and right (*P*)- handed helicity, as expected from an achiral monomer unit.

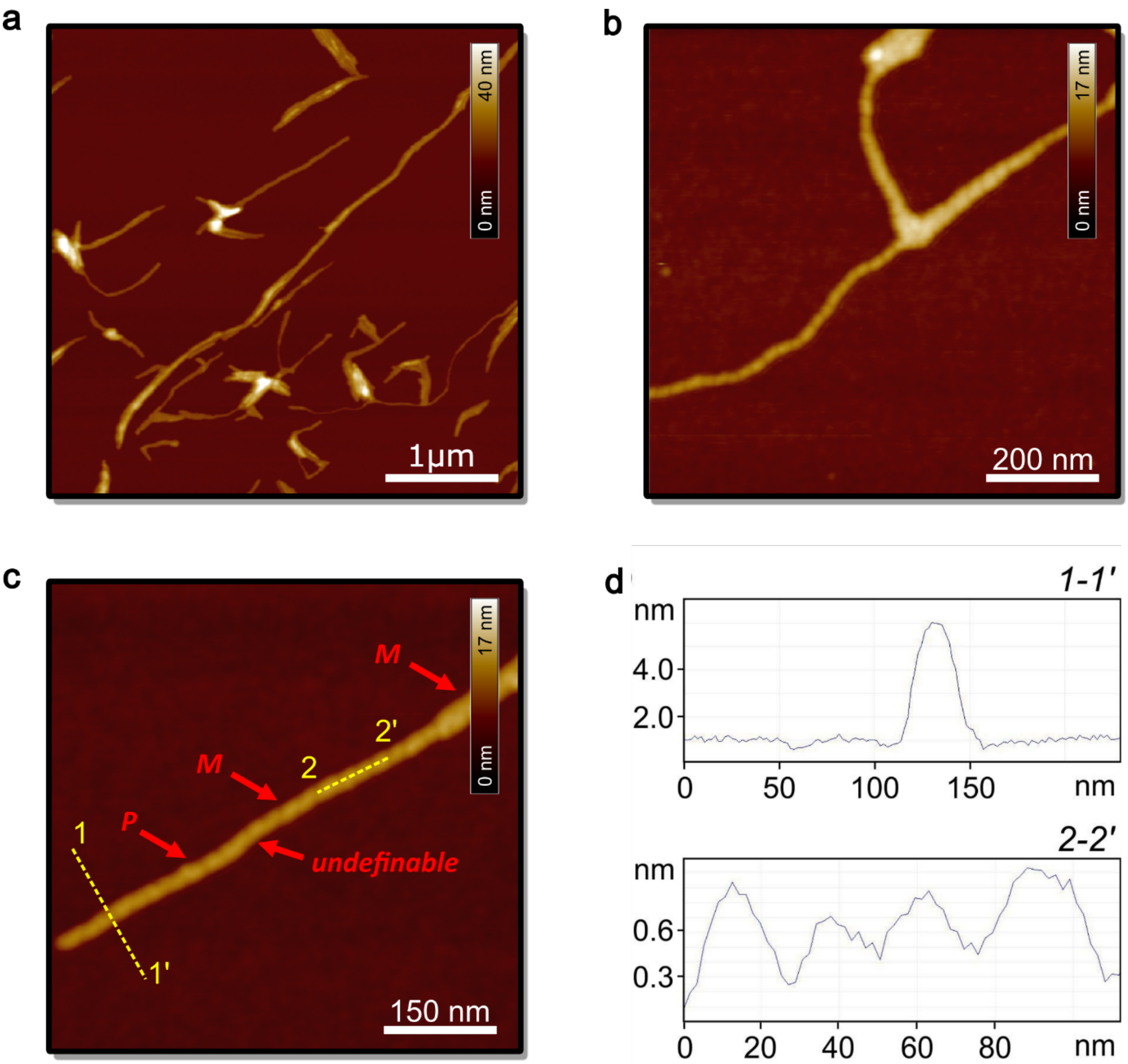

Figure S24. a-c, AFM height image of *E*-**1** spin coated (3000 r.p.m.) onto silicon substrate from a 35 μM solution in H_2O/MeCN (4:1, *v/v*) showing rope-like fibres with a diameter of 4.9 ± 0.3 nm and lengths of up to a few microns. **b** and **c,** zoomed height image *E*-**1** aggregate revealing sections exhibiting left (M)- and right (P)-handed helicity. **d,** Cross-section analyses from yellow dashed lines 1-1' and 2-2'.

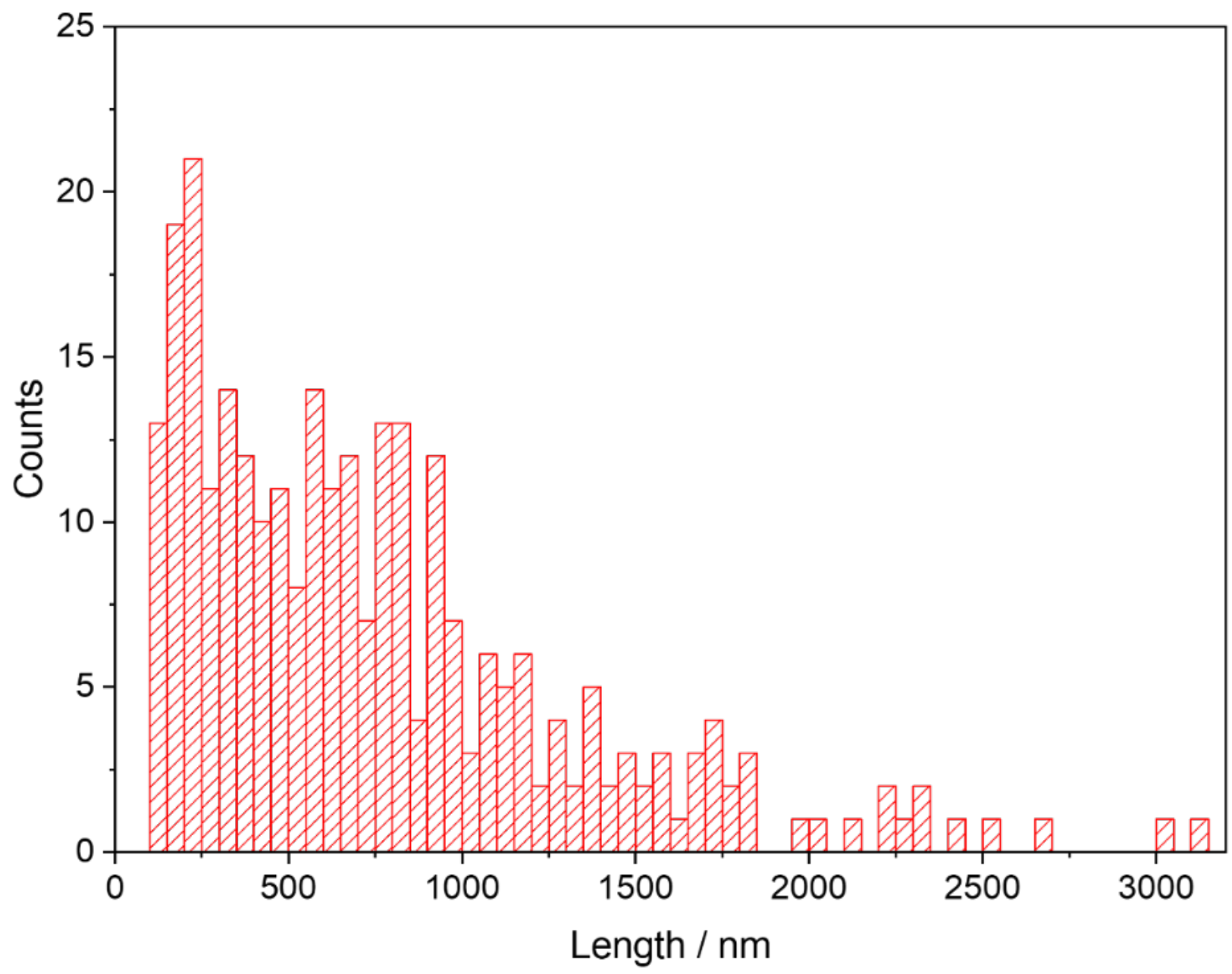

Figure S25. Histogram of the length distribution of fibre length of the *E*-**1** traced from the AFM image.

5.2.2. Morphology of *Z*-1

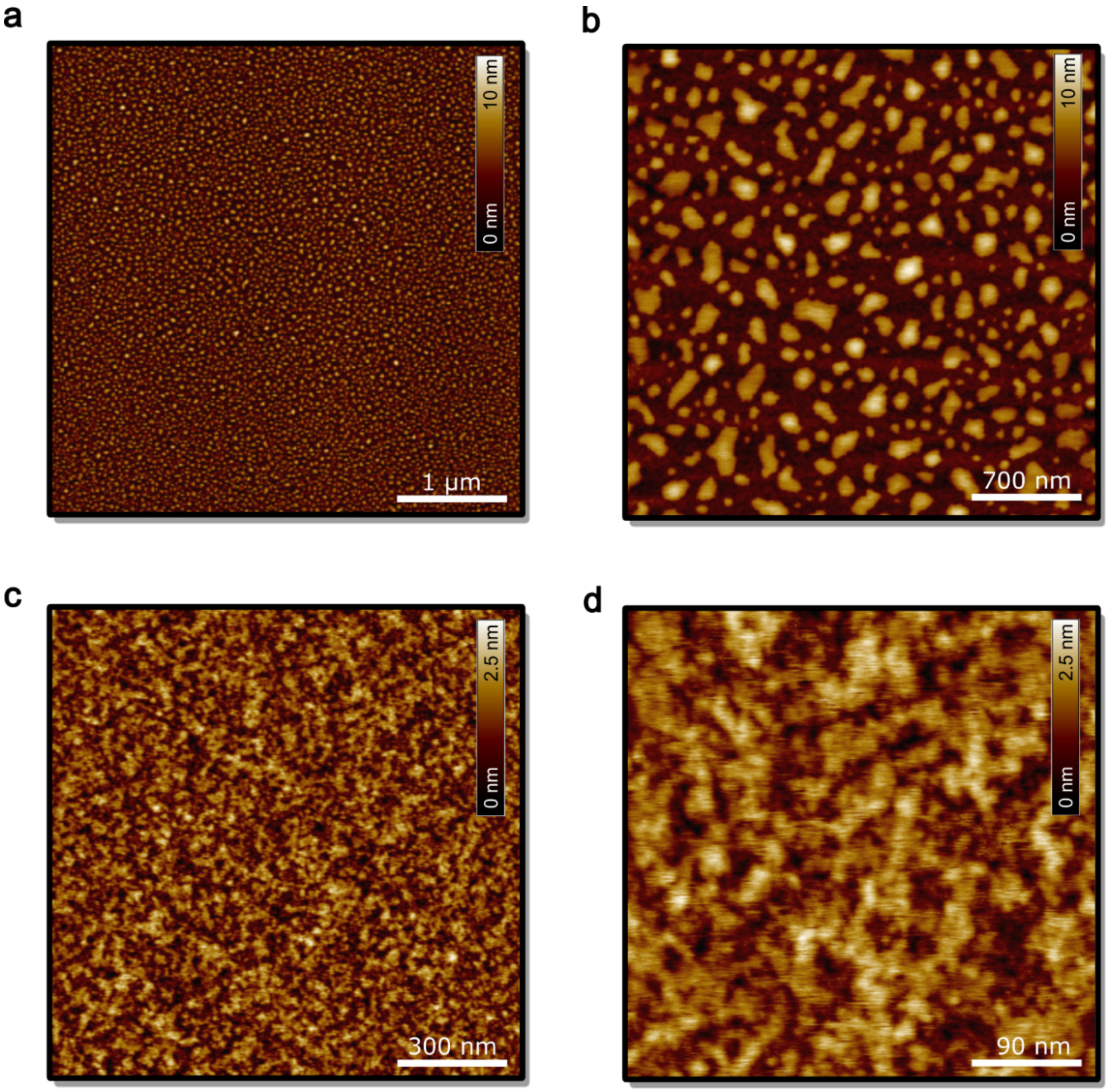

Figure S26. a-d, AFM height image of *Z*-**1** from a 35 µM solution in H_2O/MeCN (4:1, *v/v*) showing no signs of supramolecular polymerization. Micrographs **a** and **b** were recorded immediately after the spin-coating and show drops of solvent forming small globular structure with solvent. **c** and **d** zoomed height image of *Z*-**1** recorded after drying overnight.

5.2.3. AFM with in-situ irradiation

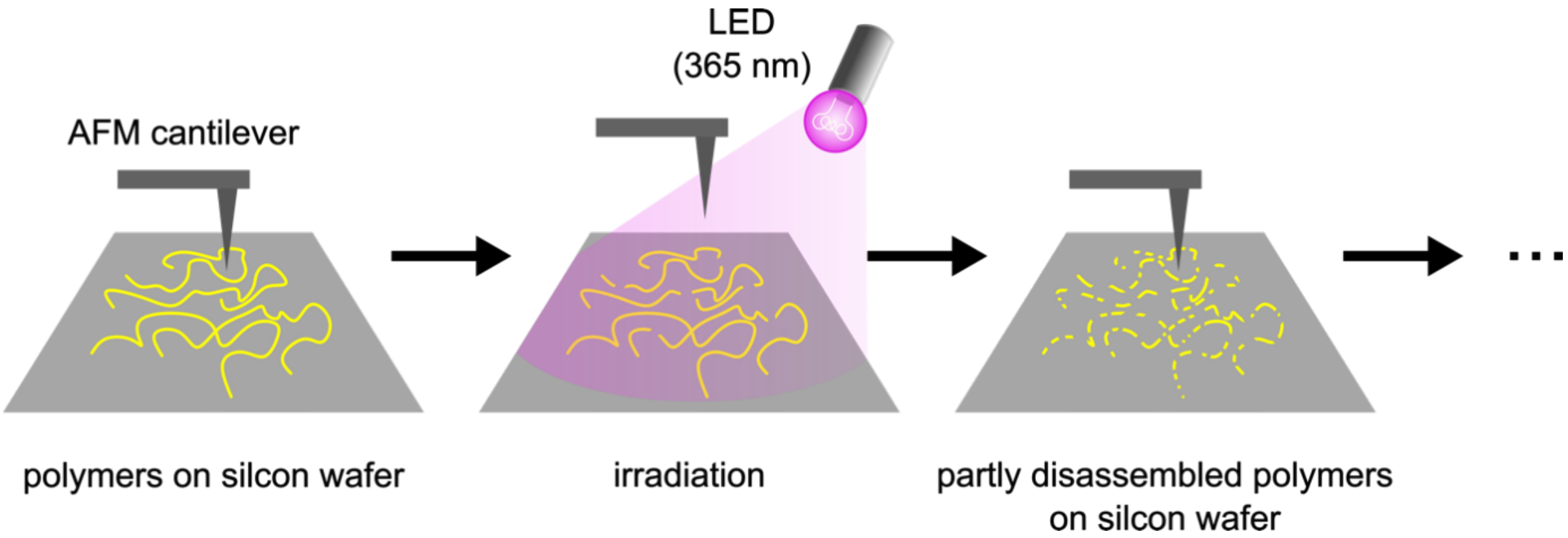

Figure S27. Schematic illustration of how the AFM with in-situ light irradiation was performed.

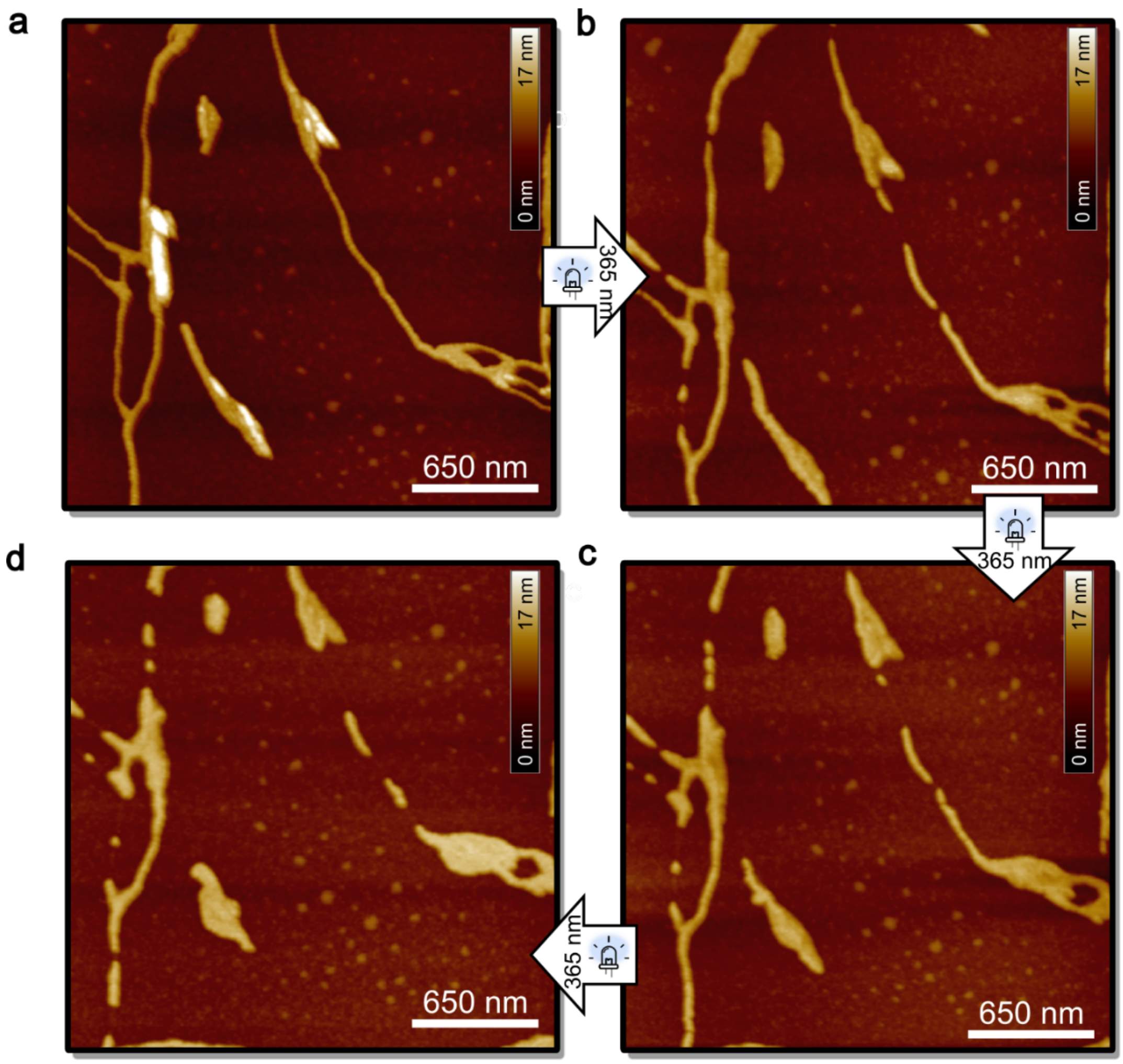

Figure S28. a-d, AFM height image of *E*-**1** spin coated onto silicon substrate from a 35 µM solution in H_2O/MeCN (4:1, *v/v*), **a,** taken before irradiation. **b**, **c**, and **d,** zoomed height image after irradiation for different time spans (3, 6, and 9 minutes respectively) showing polymer break and the clustering of the monomers into bundles. The polymers break in random positions along the fibres and lose their helicity.

5.2.4. AFM Control Experiment

To assess whether repeated AFM imaging induced structural changes to the supramolecular polymers, a representative region of the sample was scanned consecutively under identical imaging conditions prior to photoirradiation. Imaging was performed in tapping mode using a cantilever with a low spring constant. The proportional and integral gains were adjusted to maintain stable feedback while minimising tip–sample forces, and a scan rate of 0.7 Hz was employed to reduce lateral forces during imaging.

No detectable changes in the morphology of the aggregates were observed following repeated scanning (Figure S29), indicating that the imaging conditions did not induce fragmentation or deformation of the sample. These control measurements support that the structural changes observed during the in-situ photoirradiation experiments arise from light-induced disassembly rather than repeated scanning or tip-induced artefacts.

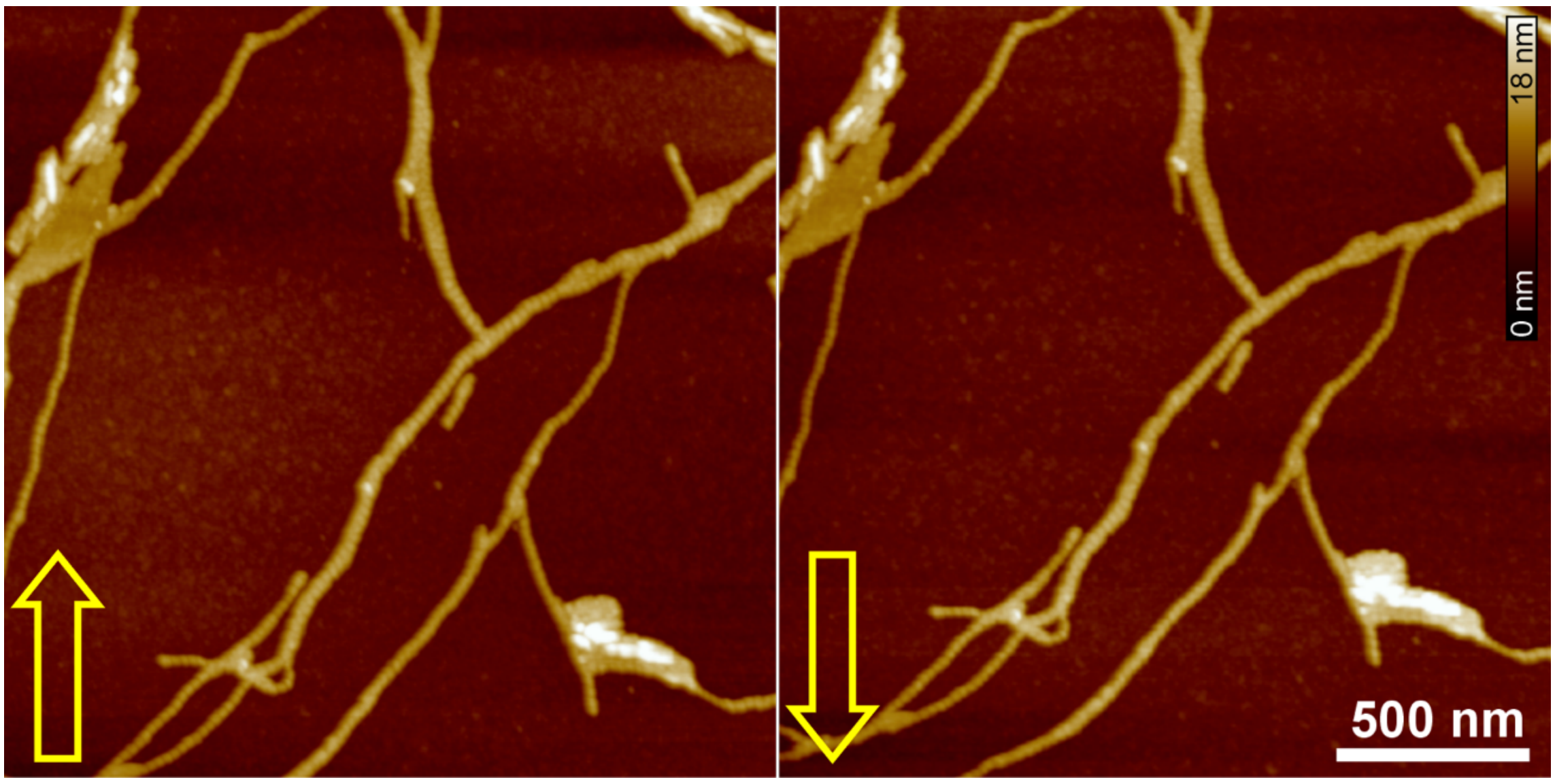

Figure S29. Consecutive AFM height images of the same region of a spin-coated film of **2** (35 μM in H_2O/MeCN (4:1, *v/v*)) acquired under identical imaging conditions prior to photoirradiation. No changes in aggregate morphology were observed between scans. Yellow arrows indicate the scanning direction.

6. PSS-modulated Aggregation

6.1 Crossing the Nucleation Threshold with Light

As *E*-**1** follows a nucleation-elongation (cooperative) mechanism for supramolecular polymerization, a minimum concentration of active species is required to initiate nucleation. Once this threshold is reached, further increase in the concentration of active species leads to growth of aggregates from nucleus.

Previous PSS studies in DMSO showed that irradiation at 365 nm results in quantitative conversion of *E*-**1** to *Z*-**1**, while irradiation with LEDs spanning 340 - 530 nm generates a range of intermediate PSSs with different *E*/*Z* ratios. These PSSs therefore contain varying proportions of active and inactive monomers, providing a means to modulate the aggregation behaviour and give an additional control over the supramolecular polymerization process using light.

To verify the existence of this light-induced control over polymerization, UV/vis absorption spectra of a 12.5 μM sample of *E*-**1** in H_2O/MeCN (4:1, *v/v*) at different PSSs were recorded. In these spectra, features corresponding to the monomer emerge upon changing the LED irradiation wavelength from 340 nm to 530 nm. Once the active monomer concentration exceeds the nucleation threshold (at irradiation wavelengths ≥ 385 nm), an aggregate absorption band appears. The degree of aggregation then increases non-linearly with longer wavelengths of light irradiation (Figure S30a).

To confirm that the hypsochromically shifted absorption band corresponds to the aggregate, temperature-dependent UV/vis measurements were performed on the same sample (12.5 μM, H_2O/MeCN (4:1, *v/v*)). At 20 °C (293 K), the spectrum recorded showed the aggregate (**2**) feature, while heating to 60 °C (333 K) resulted in the appearance of the feature corresponding to the monomer (*E*-**1**), consistent with the peaks observed in PSS-mediated aggregation studies.

Corresponding DLS measurements of 12.5 μM sample in H_2O/MeCN (4:1, *v/v*) under same irradiation sequence (Figure S30b) reveals the formation of larger species (at irradiation wavelength > 405 nm). Upon moving from shorter to longer wavelength of irradiation, the particle size increased progressively, confirming the existence of long fibres with increase in the concentration of *E*-**1** (Figure S30b**)**. This was consistent with

the UV/vis studies.

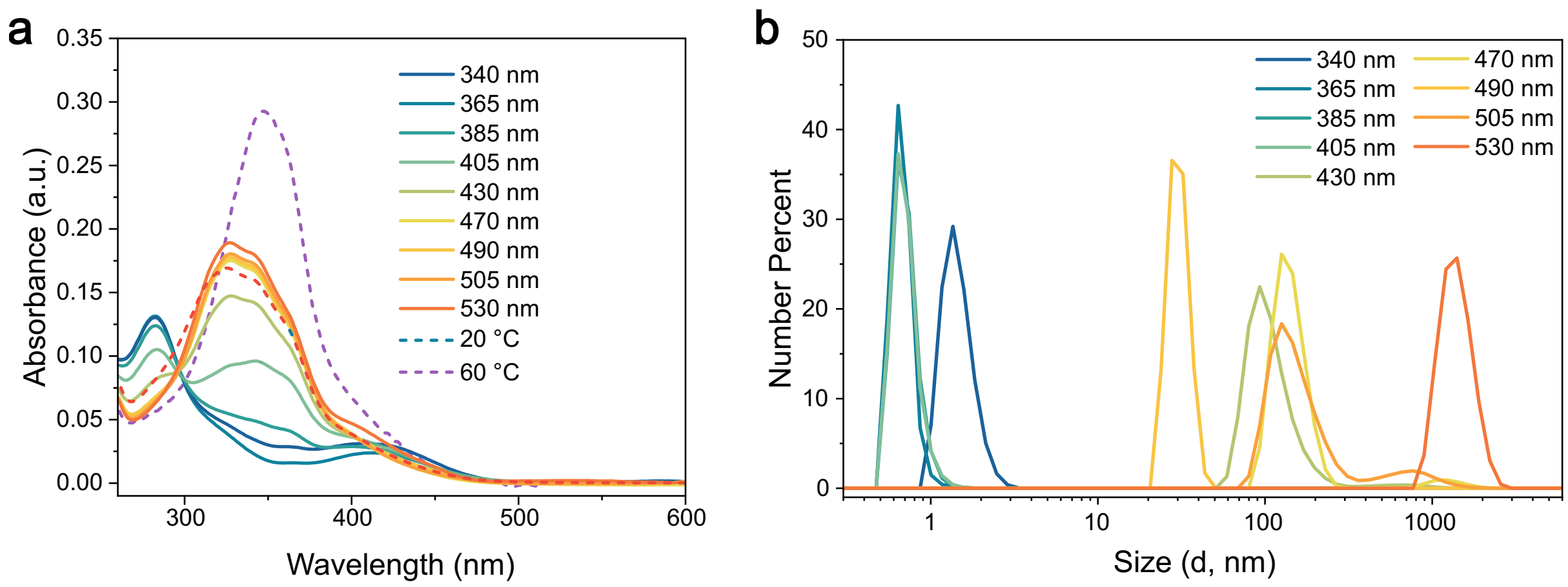

Figure S30. **a**, UV/vis absorption spectra of a 12.5 μM solution of *E*-**1** in H_2O/MeCN (4:1, *v/v*) upon irradiation with LEDs of different wavelengths (to a PSS). The dashed lines correspond to the spectra of *E*-**1** at 60 °C (333 K) and in the aggregated state at 20 °C (293 K). **b**, Changes in the DLS size distribution, expressed as number percent, of a 12.5 μM solution of *E*-**1** in H_2O/MeCN (4:1, *v/v*) after irradiation with LEDs of different wavelengths. Each irradiation was performed for 5 minutes under constant stirring prior to each PSS measurement.

Table S8. The measured count rate of the same sample (Figure S30b, from DLS) at different PSSs.

Irradiation wavelength (nm)	Count rate (kcps)
340	46.1
365	37.4
385	120.6
405	95.5
430	150.8
470	292.2
490	325.8
505	113.6
530	228.5

6.2 Resistance to Fatigue During Assembly and Disassembly

While the resistance of monomer **1** to fatigue during photoisomerism was demonstrated in Section 3.4 in DMSO, we further studied the fatigue resistance during the assembly/disassembly process in H_2O/MeCN (4:1, *v/v*) at 20 °C (293 K). Taking a 25 µM solution of monomer **1** in said solvent system, the photoswitching fatigue resistance and the assembly/disassembly was analysed.

For 10 cycles of converting between the 365 nm and 530 nm PSSs, no signs of photodegradation were observed (Figure S31a). For cycle numbers 1, 5 and 10, the DLS plots were obtained for the 365 nm and 530 nm PSSs (Figure S31b). The system maintained the ability to assemble and disassemble over these cycles. The corresponding count rates from the DLS measurements are further shown in Table S9, highlighting the presence and absence of scattering species at the different PSSs.

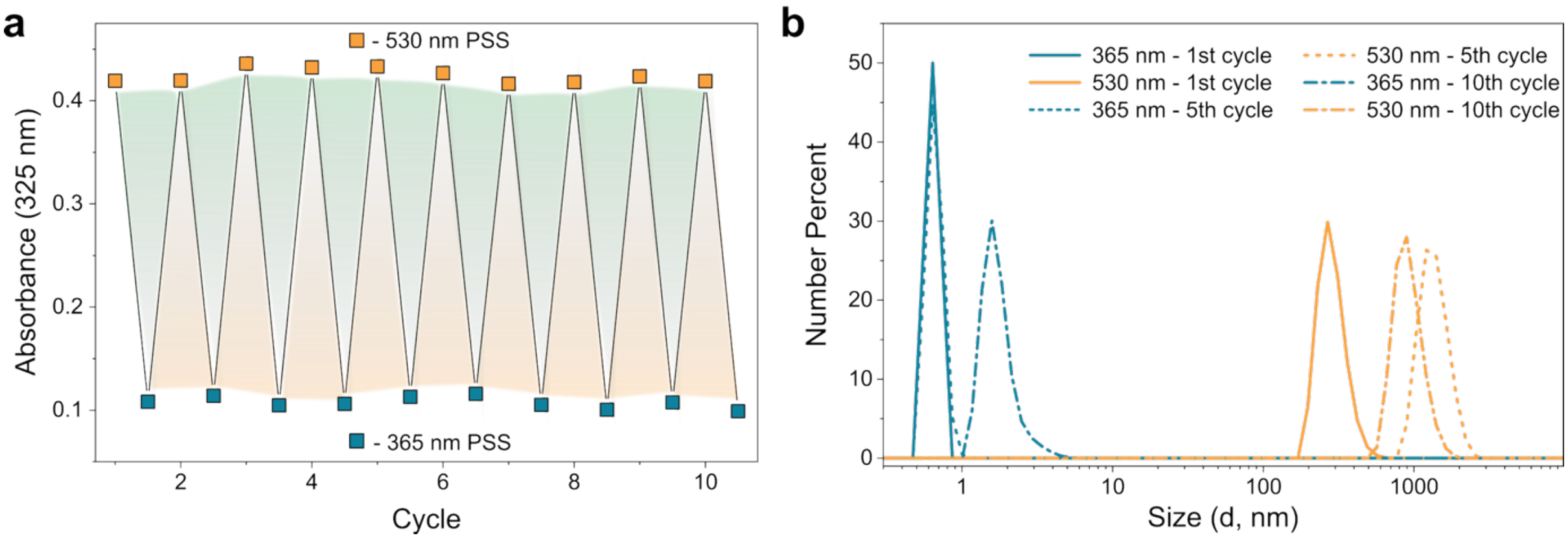

Figure S31. a, Fatigue resistance of **1** measured using UV/vis spectroscopy (25 µM in H_2O/MeCN, 4:1, *v/v*, 293 K) upon repeated photoisomerism with alternating 365 nm and 530 nm irradiation. No signs of degradation were observed over 10 switching cycles. **b**, Changes in the DLS size distribution, expressed as number percent, of a 25 µM solution of *E*-**1** in H_2O/MeCN (4:1, *v/v*) for cycles 1, 5 and 10.

Table S9. Change in the count rate of a 25 µM sample of **1** in H_2O/MeCN (4:1, *v/v*) at 20 °C (293 K) with 365 nm and 530 nm LED.

Cycle number	Irradiation wavelength (nm)	Count rate (kcps)
1	365 nm	140
	530 nm	1957
5	365 nm	55
	530 nm	986
10	365 nm	169
	530 nm	799

7. References

[1] A. Volker, J. D. Steen, S. Crespi, "A Fiber-optic Spectroscopic Setup for Isomerization Quantum Yield Determination" *Beilstein J. Org. Chem.* **2024**, *20*, 1684–1692.

[2] F. Xu, L. Pfeifer, S. Crespi, F. K. Leung, M. C. A. Stuart, S. J. Wezenberg, B. L. Feringa, "From Photoinduced Supramolecular Polymerization to Responsive Organogels" *J. Am. Chem. Soc.* **2021**, *143*, 5990−5997.

[3] F. Xu, S. Crespi, G. Pacella, Y. Fu, M. C. A. Stuart, Q. Zhang, G. Portale, B. L. Feringa, "Dynamic Control of a Multistate Chiral Supramolecular Polymer in Water" *J. Am. Chem. Soc.* **2022**, *144*, 6019−6027.

[4] C. M. A. Leenders, L. Albertazzi, T. Mes, M. M. E. Koenigs, A. R. A. Palmans, E. W. Meijer, "Supramolecular Polymerization in Water Harnessing both Hydrophobic Effects and Hydrogen Bond Formation" *Chem. Commun.* **2013**, *2*, 1963–1965.

[5] J. L. Greenfield, M. A. Gerkman, R. S. L. Gibson, G. G. D. Han, M. J. Fuchter, "Efficient Electrocatalytic Switching of Azoheteroarenes in the Condensed Phases" *J. Am. Chem. Soc.* **2021**,*143*, 15250−15257.

[6] J. Wu, J. L. Greenfield, "Photoswitchable Imines Drive Dynamic Covalent Systems to Nonequilibrium Steady States" *J. Am. Chem. Soc.* **2024**, *146*, 20720−20727.

[7] P. Van Der Schoot, A. P. H. J. Schenning, E. W. Meijer, "How to Distinguish Isodesmic from Cooperative Supramolecular Polymerisation" *Chem. Eur. J.* **2010**, 362–367.

[8] G. Fernández, M. Stolte, V. Stepanenko, F. Würthner, "Cooperative Supramolecular Polymerization: Comparison of Different Models Applied on the Self-Assembly of Bis(merocyanine) Dyes" *Chem. Eur. J.* **2013**, *19*, 206–217.

[9] R. F. Goldstein, L. Stryer, "Analytical Approximations, Numerical Examples, and Experimental Strategy" *Biophys. J.* **1986**, *50*, 583–599.

[10] E. Fischer, "Calculation of Photostationary States in Systems A→B when only A is Known" *J. Phys. Chem.* **1967**, *71*, 3704–3706.
